# Evaluating Early‐Stage Oncology Clinical Trial Designs in the Era of Project Optimus: A Scoping Review

**DOI:** 10.1002/pst.70131

**Published:** 2026-11-05

**Authors:** Marie‐Karelle Riviere, Anaïs Andrillon, Pavel Mozgunov, Emma Gerard, Moreno Ursino

**Affiliations:** ^1^ Department of Statistical Methodology SARYGA Tournus France; ^2^ INSERM, Inria, Université Paris Cité UMR 1346, HeKA Paris France; ^3^ ECSTRRA Team, INSERM U1342 IRSL Université Paris Cité Paris France; ^4^ MRC Biostatistics Unit University of Cambridge Cambridge UK; ^5^ Evidence Generation and Decision Sciences SANOFI Gentilly France

**Keywords:** adaptive design, dose optimization, dose‐finding, drug development, maximum tolerated dose, optimal dose

## Abstract

In 2021, the FDA initiated Project Optimus to reform dose optimization and selection in early‐phase oncology, highlighting that doses and schedules of new therapies are often inadequately characterized and that traditional dose‐finding approaches should be revised. The project also encourages early and comprehensive exploration of multiple doses during clinical development and advocates for a more patient‐centered approach incorporating not only toxicity but also the individual's response, biomarkers, pharmacokinetics/pharmacodynamics, or patient‐reported outcomes to inform dosage decisions. We conducted a scoping review of the statistical and methodological literature on early‐phase oncology trial designs in light of Project Optimus. The review aims to determine which key elements of the Optimus guidelines are incorporated into existing early‐phase trial design methodologies and to assess remaining methodological gaps. It also aims to address the following question: What progress has been achieved so far, and what improvements can be made in light of the directives from Project Optimus? The review was organized according to the PRISMA‐ScR guidelines. A total of 160 journals were finally retained, with 722 papers considered for the scoping review, published between January 1990 and September 2025. The database containing the reviewed papers is made available to the reader.

## 1 Introduction

Early‐phase clinical trials in oncology have traditionally focused on determining the maximum tolerated dose (MTD) of a new therapeutic agent [1]. This approach emerged from a historical focus on cytotoxic chemotherapy, where the primary objective of early‐phase trials was to escalate doses systematically until intolerable toxicities, defined as dose limiting toxicities (DLTs), were observed in a predetermined percentage of patients. The MTD was then transferred to subsequent phases to evaluate efficacy [2]. The traditional 3 + 3 dose‐escalation design, along with the adaptive model‐based and model‐assisted designs, became the gold standard in this context [3, 4, 5]. However, in recent years, the development of cancer drugs has undergone a paradigm shift. The rise of targeted therapies, immunotherapies, and personalized medicine has transformed the objectives of early‐phase trials. These novel agents may not rely on the principle that “more is better” [6]. In this context, the identification of an optimal dose (OD) that optimizes the risk–benefit trade–off of the drug or provides the desired therapeutic effect while minimizing toxicity has been further advocated. Project Optimus, an innovative initiative launched by the Oncology Center of Excellence of the US Food and Drug Administration [7], aims at reforming traditional approaches to dose optimization and selection in the development of cancer therapies. In 2024, in a guidance for industry [8], the project encourages incorporating individual responses, biomarkers, pharmacokinetics (PK), and pharmacodynamics (PD) to better inform dosage decisions and support early exploration of multiple doses. This shift requires a collaborative effort between pharmaceutical companies, regulatory agencies, and the broader scientific community to adopt new trial designs, invest in biomarker research, and embrace innovative statistical models for data analysis. In response to the Project Optimus initiative, many authors have recently published perspective articles and reviews discussing its implications for early‐phase oncology drug development. Samineni et al. [9] conducted a survey to evaluate current industry practices for oncology dose selection. Bhamidipati et al. [10] reviewed industry‐sponsored Phase I clinical trial protocols published between 2021 and 2024 to analyze dose‐finding designs and dose‐optimization strategies. Gao et al. [11] and Thall et al. [12] highlighted key challenges associated with improved dose optimization in oncology drug development. Dejardin et al. [13] and Liu et al. [14] discussed statistical design options for dose optimization in oncology clinical trials from an industry perspective and illustrated their application through applied trial examples. Lu et al. [15] and Andrillon et al. [16] proposed phase I/II designs integrating PK to optimize the dosing regimen. Finally, Zhang et al. [17], Yuan et al. [18], and Zang et al. [19] reviewed selected Phase I–II dose‐optimization designs aimed at identifying an optimal biological dose (OBD) rather than the maximum tolerated dose. Although these contributions highlight key aspects of Project Optimus, a comprehensive synthesis covering its full scope is still missing. This scoping review aims to provide a structured and objective mapping of the existing methodological literature on the design of early‐phase oncology clinical trials in the context of Project Optimus. Our objective is to assess how the key principles of Project Optimus have been considered in the methodological literature and to identify the gaps that remain in existing dose‐finding designs: what progress has been achieved so far in dose‐finding, and what further methodological developments are needed in light of the Optimus recommendations? This work does not review clinical trials, but rather focuses on methodological literature and statistical tools relevant to dose‐finding and dose‐optimization designs. It is deliberately framed as a scoping review and adopts a neutral and objective perspective. It does not aim to rank methodologies or to recommend any particular design, since the appropriateness of a given approach depends on multiple factors, including the clinical setting, trial objectives, operational constraints, and available endpoints. By mapping the existing methods, we aim to assess whether the current methodological tools are sufficient to address the challenges raised by Project Optimus and to highlight areas where additional methodological development is required.

## 2 Project Optimus

Based on the recommendations and considerations outlined in the Project Optimus documents [20, 21], we identified four overarching principles: (i) dose optimization should integrate safety and tolerability, including lower‐grade and late‐onset adverse events, antitumour activity, biomarkers, and dose– and exposure–response relationships; (ii) optimization may extend beyond the dose level itself, taking into account factors such as dosing schedules, treatment combinations, and heterogeneity across patient populations; (iii) whenever appropriate, two or more candidate doses should be formally compared, preferably within randomized trials; and (iv) dose optimisation should be addressed early in clinical development through interdisciplinary interactions and timely discussions with regulatory authorities. These key principles are consistent with the FDA guidance [8], which translates them into five more operational areas. First, clinical pharmacokinetics, pharmacodynamics, and pharmacogenomics should be assessed throughout development to characterize dose– and exposure–response relationships and identify clinically relevant sources of variability, including patient heterogeneity that can be addressed by integrating relevant covariates to identify differences across subpopulations. These analyses should be considered along with safety, tolerability, activity, and efficacy to guide dosage selection. Second, trial designs comparing multiple dosages should be considered to support the selection of an optimized dosage, with randomized parallel‐arm comparisons recommended whenever appropriate, possibly complemented by backfill cohorts to strengthen activity and safety assessment and by adaptive stopping of dosage arms. Third, safety and tolerability should be evaluated comprehensively across dosages, including persistent lower‐grade toxicities and late‐onset toxicities, dose interruptions, reductions, discontinuations, and patient‐reported outcomes (PROs). Tolerability may further be improved by adjusting the dosing schedule, for instance through intra‐patient dose escalation. Fourth, drug‐formulation considerations should ensure that the selected dosages can be practically evaluated and administered, for example through the availability of suitable dose strengths. Fifth, the appropriateness of the selected dosage should be reconsidered for subsequent indications and uses, particularly when differences in disease setting, patient population, treatment context, or concomitant therapies may affect the benefit–risk profile. Quantitative approaches, such as mechanistic (e.g., PBPK and QSP models) and semi‐mechanistic modeling, can also be used to support dosage selection.

In this review, we focus on the methodological and trial‐design implications of these recommendations, that is, on the key messages except the fourth, and on the related FDA guidance points, as they can be directly examined through the methodological characteristics of the identified designs. Discussions with regulatory agencies and practical considerations related to changes in the route of administration fall outside the scope of this work.

## 3 Methodology of the Scoping Review

Since we focus on methodological literature and statistical tools, we performed a two‐stage search. In the first stage, we identified statistical journals relevant to this field and added known medical journals that publish methodological papers. The selection of journals used in this review is described in Supplementary Material S1. Then, once the journals had been selected, we conducted a classical scoping review to extract relevant articles indexed in PubMed and Scopus published between January 1990 and September 2025. We considered as eligible all articles describing dose‐finding designs applicable to oncology drug development, and reporting statistical methodology. Application papers, which apply existing designs without introducing new methodology, were excluded. Dose‐ranging methods were also excluded, as the objective was to focus primarily on methodological designs used in the earliest stages of oncology drug development, particularly first‐in‐human studies including dose‐escalation settings. Additional details on the paper selection are provided in Supplementary Material S1. A protocol was developed prior to the review but was not registered or published; it is available in Supplementary Material S2, along with documented amendments. Before initiating the data extraction process, we defined a set of key features and corresponding items to assess each of the Optimus dimensions presented in Section 2. The items of interest are presented in Table 1 and were then systematically used to extract the relevant information from each reviewed article. Despite the systematic and structured approach used for endpoint extraction, the classification of Optimus‐related features may be subject to residual misclassification.

**TABLE 1:** List of features used to extract information from articles.

Endpoint	Categories	Comment
Main purpose	New design/Calibration/Theory/ Comparison/Review/Guidelines/Benchmark/ Programming tool/Commentary	“New design”: novel dose‐finding design or a methodological extension of an existing method, that is, an escalation procedure evaluated as a design in its own right, including when its novelty consists in adding one component (backfill, randomization, efficacy or activity endpoint, late onset endpoints, borrowing of historical information, etc.) to an existing design. “Calibration”: specification of design components (e.g., model parameters, prior distributions, stopping rules, dose allocation rules, cohort or sample size) within a design that otherwise remains unchanged. “Theory”: theoretical properties of existing designs (e.g., consistency, coherence, or convergence). “Comparison”: studies evaluating the operating characteristics of existing methods usually through simulations. “Review”: synthesis of the existing methodological literature. “Guidelines”: methodological or practical recommendations for the design and conduct of dose‐finding trials. “Benchmark”: frameworks for evaluating the performance of dose‐finding designs. “Programming tool”: presentation of the development of new software, an application, or an R package. “Commentary”: interpretation or critical discussion of an existing publication.
Toxicity endpoint	Binary/Ordinal/Continuous/ Time‐to‐event/Quasi‐binary/Quasi‐ binary/continuous/No	Type of toxicity endpoint. Combined categories (e.g., “Binary and continuous”) were used for designs handling two outcome types jointly. “Time‐to‐event” was used when the design explicitly models the distribution of the time to the event; designs that keep a binary endpoint weighted by the observed follow‐up time, such as the TITE family, were coded “Binary”.
Multivariate toxicity	Yes/No	“Yes” when the design accounts for several distinct types of toxicity, or when several toxicity grades are aggregated into a single score. “No” when toxicity grades are used as such, without aggregation; the information is then carried by the “Toxicity endpoint” item, coded “Ordinal”.
Late toxicities	Yes/No	Handling of dose‐limiting toxicities occurring outside of a short‐term evaluation window, or accounting for toxicity information at multiple treatment cycles for the dose selection.
PRO	Yes/No	Considering patient reported outcome for dose recommendation.
Efficacy endpoint	Binary/Ordinal/Continuous/ Time‐to‐event/Binary and continuous/Quasi‐binary/continuous/ Longitudinal continuous/No	Type of efficacy endpoint or surrogate of efficacy when the estimation of the recommended dose accounts for efficacy; otherwise “No”.
Multivariate efficacy	Yes/No	Incorporates multivariate patient efficacy information.
Late efficacies	Yes/No	Handling of efficacy occurring outside of a short‐term evaluation window.
Activity endpoint	Binary/Ordinal/Continuous/ Time‐to‐event/No	Type of activity endpoint when the estimation of the optimal dose is based on a biological activity endpoint or immunological marker; otherwise “No”. Pharmacokinetic quantities are captured by the “PK/PD Modeling” item.
Multivariate activity	Yes/No	Incorporates multivariate patient activity information.
PK/PD modeling	Yes/No	Incorporates pharmacokinetic and/or pharmacodynamic information for the dose selection.
Preclinical/Historical	Yes/No	Incorporates preclinical or historical information. This item describes the proposed design: if historical data are used only to calibrate a simulation study, the design was not listed here. A Bayesian design alone is not sufficient (even if it inherently allows prior information to be incorporated); incorporation of external data must be explicitly stated in the article.
Final decision criteria	MTD: Safety only/OBD: Efficacy‐driven/OBD: Utility/NA	When only toxicity outcomes were used to guide dose selection, designs were classified as “Safety‐only”, corresponding to MTD‐oriented approaches. Otherwise, for OBD oriented designs, when both toxicity and efficacy/activity were used for the decision, we further categorized them according to their decision rule: “Efficacy‐driven” approaches in which dose selection is primarily driven by the efficacy/activity endpoint under toxicity constraints, in most designs through efficacy maximization, or “Utility” approaches based on an explicit trade‐off between several criteria, typically balancing toxicity and efficacy. “NA” was used for Guidelines, Benchmark or Comparison papers.
Randomization	Yes/No/vs. SoC	Randomization of patients performed between several dosages. It differs from standard patient allocation based on deterministic rules. “vs. SoC” was used when randomization is performed against a standard‐of‐care arm rather than between dosages.
Comments randomization	During/After escalation	Patients can be either randomized from the start of the trial during dose escalation, or after a dose‐escalation stage to identify acceptable dosages, or both.
Schedules	Yes/No	In addition to the dose level itself, can account for different schedules in the trial.
Heterogeneity	Yes/No	Allowing the inclusion of covariates in the modeling or determining a subgroup‐specific recommended dose.
Design type	Algorithm‐based/Model‐based/ Model‐assisted or Curve‐free/Other/NA	Articles were classified into one of the three main categories or as approaches combining multiple design classes (e.g., Model‐based and Model‐assisted). “Other” includes designs that do not fall into any of the three main categories. “NA” is applicable for Commentary, Benchmark or Programming tool papers.
Comments design type	Free text	Applicable only to “Other” design types.
Available tool	Yes/No/Upon request/NA	Tools available to perform the design. “Yes” was used when the article points to material obtainable without contacting the authors. “Upon request” when the material is announced as available from the authors on request; an announcement in the future tense was coded “No”. “NA” is applicable for Commentary, Review, Comparison, Theory or Guidelines papers.
Comment tool	Code/Rpackage/App/ Upon request/NA	Type of tool available when “Yes” was selected for “Available tool”.

## 4 Results

From the SCOPUS search, a list of 335 journals was extracted in total (cf. Table 1 from Supplementary Material S1). After independent review by the authors, 154 journals were retained (see “TRUE” in the second column of Table 1 of Supplementary Material S1). In addition to the above, based on the authors' knowledge of the literature on dose‐finding designs in oncology, some methods were known to have been published in medical journals. Therefore, six clinical journals known for publishing statistical methods were included in the list of relevant journals (see Table 2 of Supplementary Material S1). In total, 160 journals were considered for this review (cf. Figure 1). From the 160 statistical and medical journals identified, 1006 records were retrieved. Following independent screening, duplicates, out‐of‐scope records (e.g., Phase II studies), and applications to clinical trials using existing designs were excluded, resulting in 691 articles included in the review. In addition, 31 records identified through reference lists of included articles or through the authors' knowledge, and originating from medical or pharmacokinetics journals not covered in the main search, were assessed for eligibility and included. Overall, this process resulted in 722 articles considered to be within the scope of the review (cf. Figure 1). The complete list of included articles is provided in Supplementary Material S1. The database containing the 722 reviewed articles is available here: Supplementary Material S3.

**FIGURE 1:**
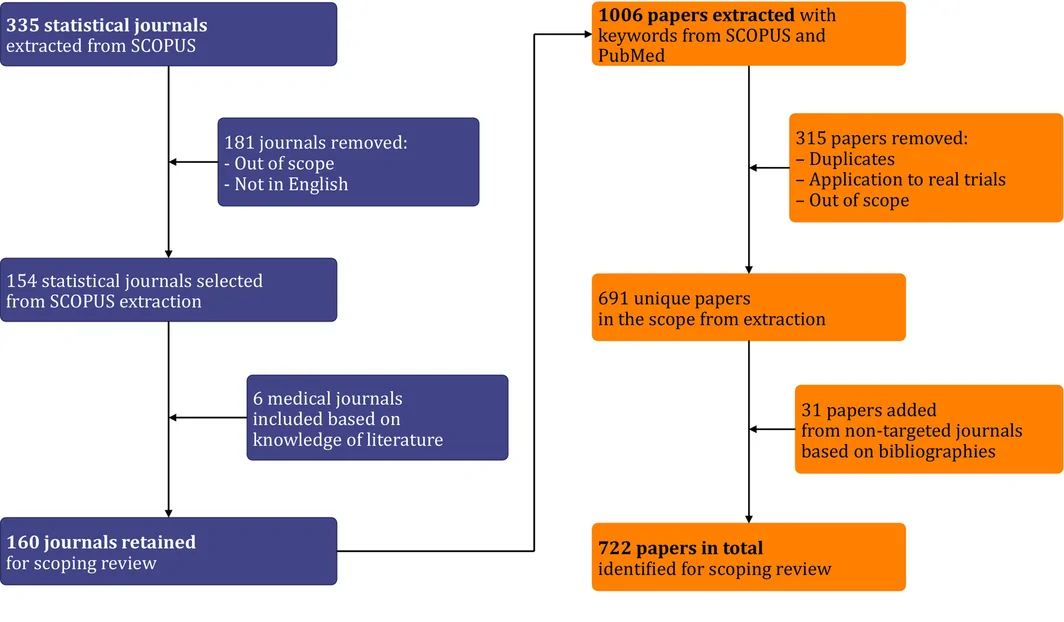
Journal and article identification, screening, and selection workflow (literature search conducted from January 1990 to September 2025).

### 4.1 Methodology Contribution Over the Years

Figure 2 shows papers classified by their primary purpose (see Table 1 for a detailed description of the different categories of paper purpose). “New design” dominates at around 68% of the total, highlighting how a significant majority of published work focuses on proposing or refining methodological frameworks. Subsequent categories, such as “Calibration” (7.5%), “Theory” (5.1%), and “Benchmark” (1.1%), underscore the broader methodology that goes beyond the dose‐finding design proposal and addresses the optimization of existing methods. “Comparison” (7.3%), “Review” (3.5%), “Guidelines” (2.9%), and “Commentary” (2.5%) papers also reflect the research community's efforts to synthesize knowledge and evaluate methodological approaches.

**FIGURE 2:**
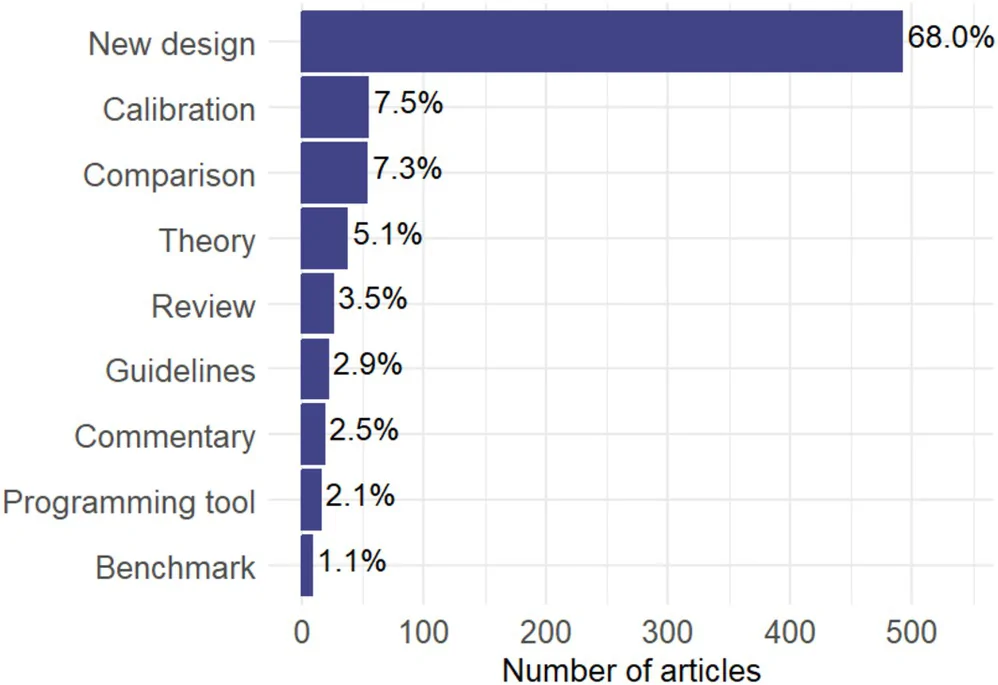
Main purpose of the published articles.

Figure 3 provides a comprehensive overview of the evolution in published papers over the past few decades. It shows both the annual number of publications and the cumulative total, highlighting the steady increase in research activity on dose‐finding designs. The yearly publication rate was relatively modest during the 1990s, with only a few articles emerging each year. However, since the early 2000s, the number of publications has begun to rise, reaching over 20 per year during the 2010s. Since 2017, more than 40 papers have been published annually. Monotherapy appears to have initially dominated the landscape in the 1990s, likely reflecting the then‐prevalent approach of dose escalation for single investigational agents. Monotherapy papers refer to methods in which only one experimental drug is escalated, whereas papers addressing more than one agent refer to combination methods in which multiple agents may be escalated. Over time, combination therapy began receiving increasing attention, particularly in the 2010s, when it became more evident that complex biological pathways often require multi‐agent regimens. In particular, the cumulative total number of papers shows a steeper increase in recent years, indicating growth in both monotherapy‐ and combination‐based designs. Figure 3 also illustrates the evolution of design classes over time. Initially, algorithm‐based designs were developed, in which dose escalation is empirically determined, including various versions of the 3 + 3 algorithm [22, 23, 24, 25, 26]. Algorithm‐based methods represent less than 10% of new design publications (cf. Figure 8B, for the distribution of design classes in dose‐finding methodologies). Subsequently, model‐based approaches were introduced, explicitly specifying assumptions on the dose–toxicity relationship (and/or dose–efficacy relationship) to enable information sharing across dose levels. These include different versions of the continual reassessment method (CRM) [27, 28, 29, 30, 31, 32]. Model‐based designs clearly dominate the methodological literature, accounting for more than 60% of all new design publications. Around 2010, model‐assisted or curve‐free approaches emerged and have since been the source of many publications in dose‐finding, and from 2017 represent nearly 20% of new designs proposed. This category includes interval‐based designs such as the toxicity probability interval design [33], the Bayesian Optimal Interval (BOIN) design [34], and the Keyboard design [35]. The remaining designs shown in Figure 8B, correspond to hybrid designs combining features from at least three of the above categories (3.7%) and to a heterogeneous “Other” category (4.9%) grouping approaches that do not fit within the algorithm‐based, model‐based, or model‐assisted classification. This includes semiparametric methods [36, 37], Bayesian optimization (Gaussian process‐based) designs [38, 39], multi‐armed bandit [40, 41, 42] and reinforcement learning designs [43, 44, 45], and Bayesian stochastic approximation designs [46].

**FIGURE 3:**
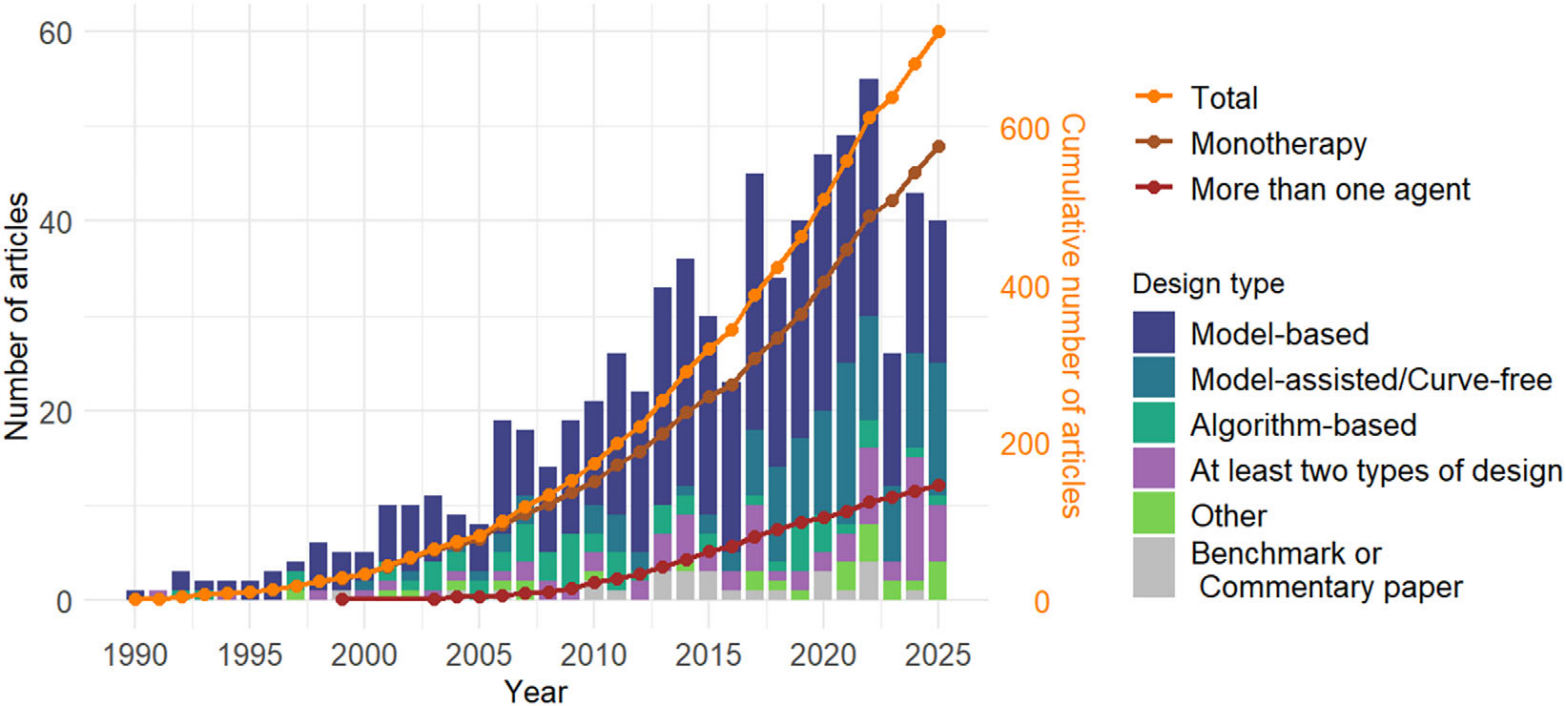
Number of papers published over the years (from January 1990 to September 2025) according to design type. The primary y‐axis (on the left) denotes the number of papers published each year, while the secondary y‐axis (on the right) tracks the cumulative number of articles over the entire period.

### 4.2 Key Topics of the Optimus Project

Figure 4 provides an overview of published papers proposing new designs or extensions of existing methodologies, categorized according to the features extracted from Project Optimus. In total, 491 such methods were identified. For each feature, the number of papers addressing that feature is shown in the horizontal bars on the right‐hand side of the plot. The vertical bars at the top represent the number of papers corresponding to each combination of features, while the colored dots indicate whether a given feature is covered by those papers. For instance, we identified 105 methods dealing with toxicity only (brown bar), and 52 focusing exclusively on toxicity and efficacy endpoints, without considering any other design features (first green bar). More broadly, 199 methods use both toxicity and efficacy for dose recommendation, possibly alongside other features. Most papers address two to three key topics, whereas only a small number simultaneously cover a broader range of features. Fifty‐one papers are devoted exclusively to late‐onset toxicities, 49 papers proposed designs for combinations based on toxicity endpoints only, and 25 papers handle toxicity while taking into account possible heterogeneity in dose determination through inclusion of covariates in the modeling or consideration of subgroups. The most frequent three‐feature intersection (dark blue bars) is toxicity plus efficacy and late‐onset evaluation, found in 20 methods, followed by toxicity–efficacy and randomization (17 papers), toxicity–efficacy and combination designs (11 papers) or toxicity–efficacy and heterogeneity (10 papers). To summarize, several key features emphasized by Project Optimus are already well covered in the methodological literature. For instance, 199 papers proposed incorporating efficacy endpoints (in addition to a toxicity endpoint) into dose selection and 31 considered activity endpoints, reflecting the First and Second areas of the FDA guidance, which recommend optimizing dosage beyond toxicity alone. We identified 86 methodologies incorporating randomization, either during or after dose escalation, which may support more robust comparisons across dose levels and improve dose optimization, in line with the Second area, on comparing multiple dosages whenever appropriate. Methods for handling late‐onset toxicities are also well developed, with 132 publications addressing this issue, consistent with the Third area, on comprehensively evaluating safety and tolerability. Still within the scope of safety assessment, 89 methods considered more granular toxicity information than a simple binary DLT endpoint, including ordinal, continuous, or quasi‐continuous outcomes (cf. Section 4.3 for more details). In addition, 31 methods have been published to optimize not only the amount of the drug but also the dosing schedule. Finally, 66 methods explicitly take into account heterogeneity in patient populations in dose selection, as recommended in the First and Fifth areas of the FDA guidance. However, other aspects of the guidance remain comparatively less explored. In particular, only 23 methods incorporated PK information into the dose‐finding process to characterize dose– and exposure–response relationships, despite this being a key component of the First area. Similarly, the integration of PROs into dose‐decision processes, addressed in the Third area, remains limited, with only 4 identified methodologies. Figure 5 provides a temporal overview of the key design features highlighted by the Project Optimus. Most of these features were already being explored in the literature well before the FDA officially launched the Optimus initiative. As early as 2005, topics such as the use of efficacy endpoints for dose selection, the consideration of late‐onset outcomes, and designs for combination agents began to emerge in the literature. Randomization in dose‐finding, heterogeneity, and evaluation of dosing schedules have been studied for more than 10 years. However, the integration of PROs, external data sources, and PK/PD information into the dose‐finding process has only recently gained attention.

**FIGURE 4:**
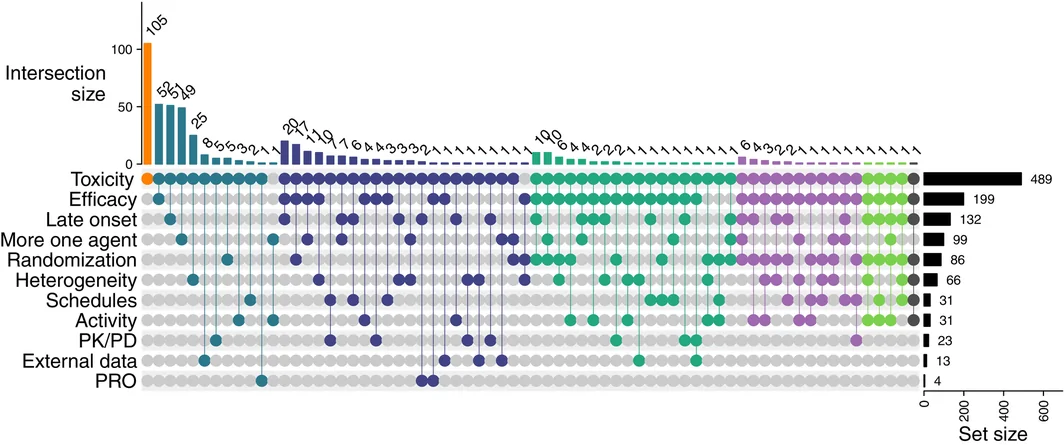
Intersection of Optimus key design features in early‐stage oncology trials papers proposing new dose‐finding designs. On the left, each key topic highlighted by the Optimus project is listed. The vertical bars at the top represent the number of papers corresponding to each combination of topics, while the colored dots indicate whether a given topic is covered by those papers. The horizontal bars on the right show the total number of papers addressing at least each individual topic.

**FIGURE 5:**
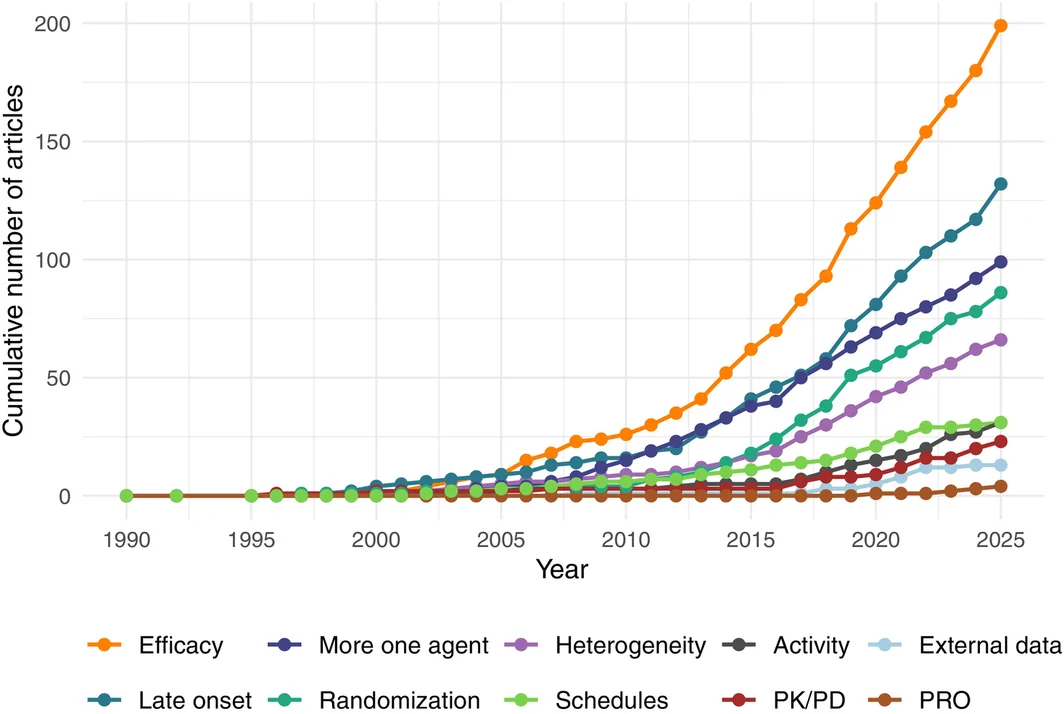
Optimus features over time, for “New design” papers.

### 4.3 Assessing Safety Only

Conventional Phase I trials traditionally prioritize toxicity to determine the recommended dose and are usually based on a binary toxicity endpoint, which is often defined as a grade 3 or higher AE that occurs within the first treatment cycle according to the Common Terminology Criteria for Adverse Events. As illustrated in Figure 7, toxicity is modeled as a binary outcome in over 80% of the reviewed articles. However, more granular toxicity endpoints can provide a more comprehensive assessment of safety. Adverse events categorized into grades can be used through ordinal outcomes [47, 48, 49, 50]. Adverse events subdivided into organ or symptom categories can be summarized using severity scores, including total toxicity burden (TTB), a weighted sum of toxicity grades, and total toxicity profile (TTP), defined as the Euclidean norm of toxicity weights [51, 52, 53, 54, 55]. Toxicity counts can also be considered [56]. The choice of toxicity endpoint should balance clinical interpretability and statistical relevance, as each option has its own advantages and limitations. In line with this perspective, Project Optimus encourages the inclusion of low‐grade symptomatic toxicities in the safety evaluation to better capture tolerability profiles of targeted agents.

Beyond selecting an appropriate toxicity endpoint, the duration of the toxicity assessment window is a major component of safety assessment. Accounting for late‐onset toxicities is particularly important with targeted therapies and immunotherapies, which are administered over longer periods compared to cytotoxic agents. In such settings, toxicity may occur after the first cycle of treatment, making traditional early toxicity assessment inadequate. Several Phase I designs have thus been proposed for delayed toxicity outcomes. A first approach is to allow for continuous accrual and real‐time dose assignment for new patients when previous patients' outcomes are pending in order to shorten trial duration. In this case, the toxicity outcome is considered as binary, and two strategies are commonly used: (i) weighting the observation with incomplete follow‐up time with a ‘partial credit’ [30, 57, 58] or (ii) considering unobserved toxicity as missing data and applying appropriate missing data methods [59, 60]. Overall, 115 papers proposing new designs that account for late‐onset toxicities were identified in the review (cf. Figure 6), 74 of which used a binary toxicity endpoint. Another approach is to model toxicity as a time‐to‐event endpoint that accounts for the timing of the toxicities in the dose selection process [61, 62, 63, 64]; 22 such papers were identified. Several methods have also been proposed to model toxicity over multiple treatment cycles, either through cumulative [61, 65, 66, 67] or conditional probabilities [68]. A total of 34 papers proposing new designs for this purpose were identified.

**FIGURE 6:**
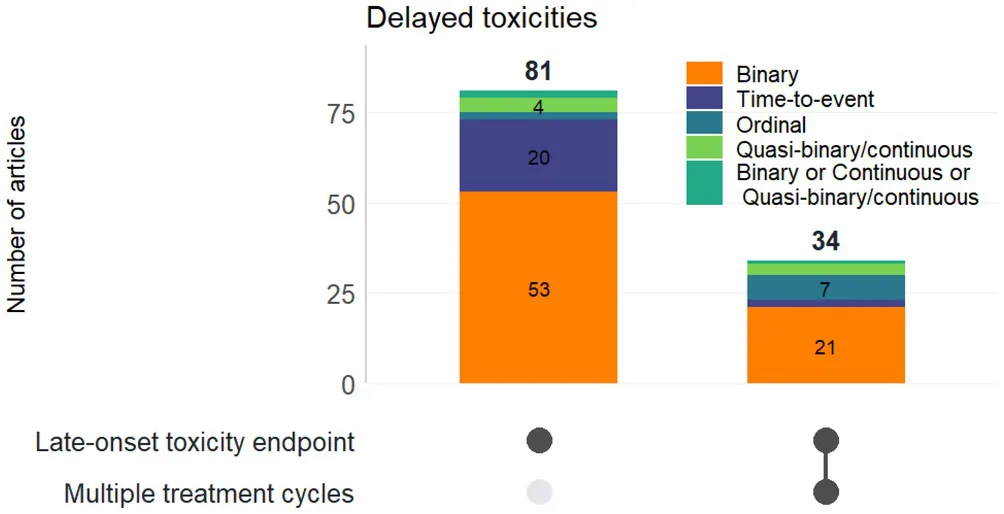
Types of toxicity endpoints in papers proposing new designs handling delayed‐onset toxicities.

### 4.4 Integrating Patient Reported Outcomes (PRO)

A key aspect of Project Optimus is its focus on safety and tolerability, notably through the integration of PROs in dose‐finding clinical trials. The inclusion of PROs in early‐phase studies has been particularly encouraged with the development of validated instruments, such as the PRO version of the Common Terminology Criteria for Adverse Events (PRO‐CTCAE) [69]. This approach offers a promising way to ensure that tolerability assessments reflect not only the clinician's perspective but also the patient's experience. Project Optimus particularly encourages the use of PROs to guide dose optimization in settings where subjective toxicities are anticipated. However, the literature on the use of PROs for dose determination designs remains very limited. Our review identified only four methods proposing PRO‐based dose‐finding approaches for MTD determination [70, 71, 72, 73], with one applied paper discussing the methodological use of such a method in a real clinical trial setting [74], and a guideline paper mentioning the need for PROs [13].

### 4.5 Combining Several Agents

Clinical trials investigating combinations of therapeutic agents have garnered renewed attention in oncology, as a promising approach for improving cancer treatment outcomes. However, the identification of an effective dose combination is difficult due to the limited sample sizes commonly used in early‐phase clinical trials. Indeed, in addition to the number of agents being considered, a major challenge lies in ordering the dose‐limiting toxicity probabilities across the dose levels when the dose of one drug is escalated while the dose of another is de‐escalated. This multi‐dimensional problem introduces additional complexity that goes beyond the scope of a single‐agent escalation design, and dose‐finding methods specifically tailored for drug combinations need to be considered. Phase I clinical trial designs for combinations of therapeutic agents have been extensively developed. In our review we identified 146 papers, including 99 papers proposing new dose‐finding designs specifically for combination therapies. Among these 99 new designs, approximately 65% were developed to identify the MTD, whereas 35% aimed to identify the optimal biological dose (OBD). These approaches include up‐and‐down designs [75, 76], model‐based designs [77, 78, 79, 80, 81, 82, 83, 84, 85, 86] or more recently model‐assisted designs [87, 88].

### 4.6 Incorporating Efficacy/Activity for Dose Decision

The primary goal of early‐phase trials is to identify a dose that balances safety with therapeutic potential, ideally translating into improved overall survival in cancer patients. Consequently, such trials may incorporate preliminary efficacy signals or surrogate markers to guide dose selection. One of the key recommendations from Project Optimus is to include efficacy data when selecting the recommended dose. Out of 491 papers proposing a new design, 199 (41%) considered both toxicity and efficacy endpoints in the dose recommendation. In this section, efficacy corresponds to early efficacy or a surrogate of efficacy. When two efficacy outcomes are considered in the dose‐finding process, we refer to efficacy and activity.

In early‐phase trials, efficacy is often modeled as a binary outcome, representing more than 70% of the articles reviewed (cf. Figure 7), typically involving short‐term binary endpoints, such as the objective response rate or the dichotomization of more complex outcomes. Otherwise, 11.1% of the methods consider efficacy as a time‐to‐event outcome, 6% as an ordinal outcome and another 6% as a continuous variable. The remaining methods considered less frequently used outcome types, including quasi‐binary and quasi‐continuous endpoints, as well as longitudinal continuous outcomes. When using a time‐to‐event outcome, it is most commonly progression‐free survival (PFS) [89, 90, 91, 92, 93]. Ordinal efficacy outcomes typically rely on disease‐specific response criteria, such as RECIST for solid tumors or hematologic response criteria (e.g., Cheson or iwCLL classifications for hematologic malignancies), including categories such as progressive disease, stable disease, partial response, or complete response [94, 95, 96, 97]. Continuous efficacy outcomes, on the other hand, are often based on quantitative biomarkers [98, 99, 100]. Regardless of the type of outcome, some designs remain intentionally general, without specifying the exact efficacy endpoint considered, highlighting the flexibility or abstraction in model formulation at the methodological stage. As for the toxicity endpoint, the duration of the efficacy assessment window is a major component of efficacy assessment and designs for handling delayed responses have been proposed in the literature. In the review, 51 methods were identified that propose new designs addressing late efficacy endpoints. Some of them are extensions of the TITE‐CRM to bivariate outcomes [101], others model‐based approaches using survival endpoints [91, 102], efficacy trade‐off designs [103, 104], or model‐assisted designs [105, 106] for delayed efficacy and toxicity outcomes. We identified a total of 200 new designs using toxicity information together with either efficacy (or a surrogate of efficacy) or activity information for dose recommendation, including OBD selection. As shown in Figure 8, the majority of newly proposed designs were MTD‐oriented and relied exclusively on toxicity data for dose selection (59.3%). However, since around 2010, there has been growing interest in OBD‐oriented methodologies that integrate efficacy information alongside toxicity outcomes. These approaches accounted for 40.7% of all new designs identified in the review. In general, two main decision‐making approaches can be distinguished in Phase I/II designs when accumulated efficacy and toxicity outcomes are used to adaptively determine doses for subsequent patients and to identify the OBD. In the first approach, the goal is usually to identify the dose that maximizes the probability of efficacy while maintaining acceptable safety [91, 101, 102, 107, 108, 109, 110, 111, 112, 113]. We refer to these as efficacy‐driven designs. We found 91 efficacy‐driven designs in the review. In some specific cases, when toxicity and efficacy are modeled as time‐to‐event outcomes, the optimal dose could be the one that maximizes the ratio of the areas under the survival curves of toxicity and efficacy up to the prespecified follow‐up time, among doses satisfying safety conditions [102]. The second approach aims to identify the most ‘desirable dose’, balancing efficacy and toxicity, and is referred to as a utility‐based design. To quantify dose desirability, designs rely either on efficacy/toxicity contours [103, 104, 114, 115] or on utility functions to assess the risk–benefit trade‐off [89, 96, 116, 117, 118, 119, 120, 121, 122, 123]. We identified 109 utility based designs in the review. Different utility functions can be used to quantify the benefit risk of dose levels. Utility‐based approaches can be defined through a combination of the estimated probabilities of toxicity and efficacy, where weights reflect the relative importance assigned to each outcome in the benefit–risk trade‐off [116, 118]. Another approach defines a utility function by eliciting physician‐defined desirability scores for discrete toxicity efficacy outcome pairs, using reference anchors corresponding to the most and least desirable scenarios (e.g., maximum efficacy with minimal toxicity versus minimal efficacy with high toxicity) [119]. The utility can also be computed as a weighted average of the utilities associated with the possible toxicity–efficacy outcome combinations (e.g., toxicity and efficacy, toxicity without efficacy, no toxicity with efficacy, and neither toxicity nor efficacy), where the weights correspond to the joint outcome probabilities [120]. A simpler approach defines a “most successful dose” (MSD) by combining toxicity and efficacy into a joint probability of success, typically defined as the probability of efficacy in the absence of toxicity, and selects the dose that maximizes this success probability [124, 125, 126]. As mentioned above, tumor response is commonly used as the primary efficacy endpoint in many Phase I–II clinical trials. However, to more thoroughly evaluate therapeutic effectiveness, both efficacy and activity can be included in the dose‐finding process. In our review, we identified 31 such methods. Activity may reflect immune response [127, 128], biomarker changes [62, 119, 129], or pharmacodynamic (PD) effects [130]. When available, PD biomarkers can serve as indicators of activity that are specific to certain cancer types, for example, protein‐derived tumor markers such as prostate‐specific antigen in prostate cancer or M protein in multiple myeloma.

**FIGURE 7:**
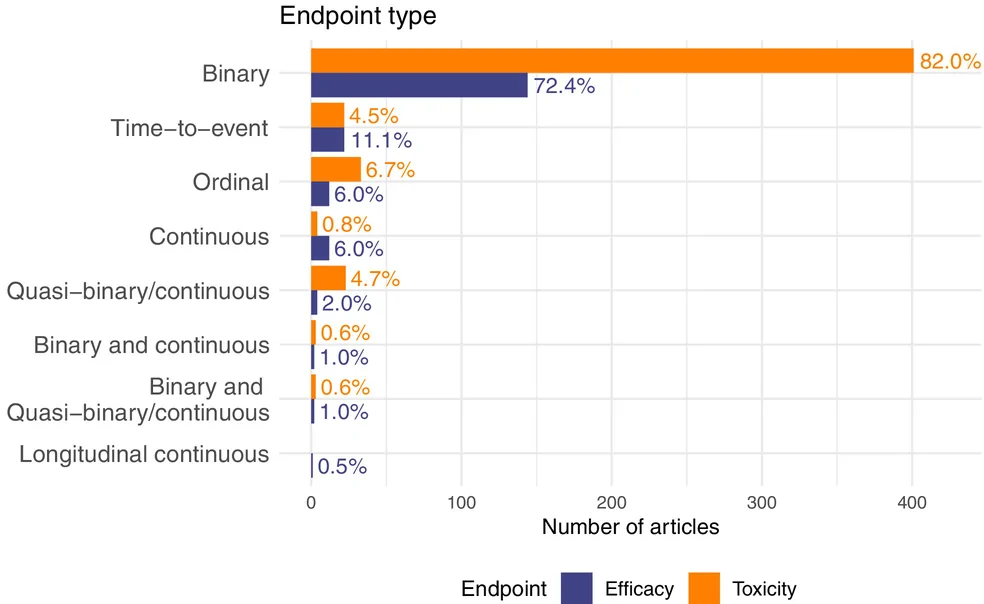
Type of endpoints in papers proposing new designs. A total of 489 papers proposed a new method and considered toxicity as an endpoint; among these, 199 also included efficacy endpoints.

**FIGURE 8:**
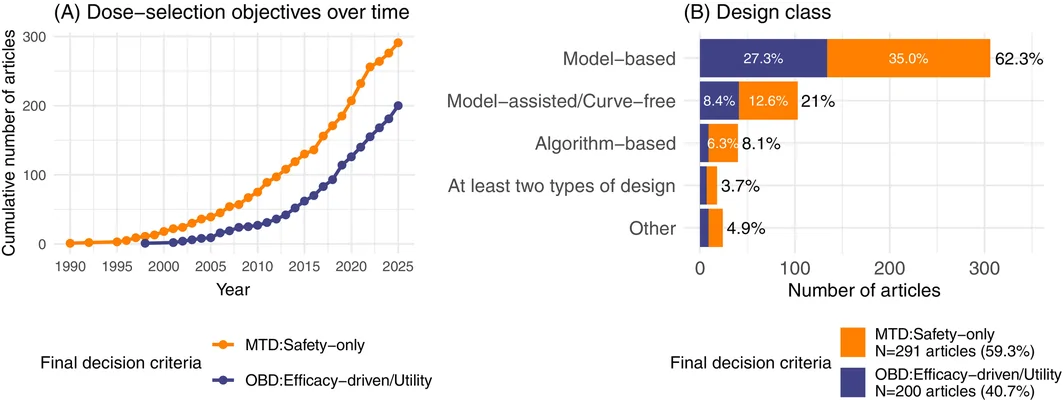
(A) Cumulative number of new methods published over time, categorized by final dose‐selection objective (MTD‐oriented vs. OBD‐oriented). (B) Distribution of MTD‐oriented and OBD‐oriented designs across design classes among new‐design publications identified in the scoping review (*N* = 491).

### 4.7 Accounting for Patient Heterogeneity

The Optimus project encourages consideration of patient heterogeneity in dose‐finding. In our review, we identified 66 articles proposing new designs that explicitly accounted for patient heterogeneity during the dose‐finding process [95, 131]. Some approaches involve the use of biomarkers as indicators of treatment heterogeneity in oncology [95, 129]. Two types of biomarkers can be distinguished based on their trajectories. The first type includes tumor‐derived biomarkers, which reflect the dynamics of the tumor. These biomarkers typically decrease in response to effective treatment, but may subsequently increase again when the tumor develops resistance and progresses. The second type comprises treatment‐related biomarkers, which reflect the direct pharmacodynamic or pharmacokinetic action of therapy, and often show an initial increase after treatment administration, followed by a decrease as resistance emerges and disease progression occurs. Such biomarkers can help characterize dose‐activity or dose–response relationships and complement toxicity data to guide dose selection.

Some designs go further by aiming to identify subgroup‐specific optimal doses [132]. Rather than recommending a single dose for all patients, these designs allow for personalized dosing based on differences in tumor type, disease subtype, or disease stage, as well as patient characteristics such as biomarker expression, age, or sex. Treatment setting, including prior or concomitant therapies, and variability in susceptibility to toxicity among patient groups [133, 134, 135] can also be taken into account. Often, the subgroups are specified before the trial starts rather than estimated.

### 4.8 Integrating PK/PD Information

Although traditional dose‐finding trials focus on modeling the endpoint of interest (toxicity, efficacy, etc.) as a function of the dose, doses are typically chosen after a careful PK or PK/PD analysis. Indeed, sponsors can use pre‐clinical data to define target saturation points and exposures to narrow the range of doses for further clinical evaluation. In addition, these analyses are often required by regulatory agencies during the submission process. The idea of including PK measures of exposure, such as the area under the curve (AUC), at least as covariates in toxicity models originated in the 1990s [136]. Another approach consists of modeling the dose–exposure and the exposure–endpoint relationships and then combining them to obtain the population‐level dose–endpoint relationship [137, 138, 139, 140]. Extensions to the time‐to‐event setting have also been proposed [141, 142].

### 4.9 Determining Dose Schedule

Designs have also been developed to determine the optimal number of treatment cycles along with the dose administered within each cycle. The number of treatment cycles, together with the predefined method of administration of the study medication within each cycle, can be referred to as the schedule [143, 144]. More broadly, the schedule corresponds to the method and timing of dose administration, including the recommended interval between doses and the overall duration of treatment, as outlined in the FDA Project Optimus guidance [145]. In a dose–schedule finding trial, the objective is to identify an optimal combination of the amount of drug administered and the administration schedule that is both tolerable and sufficiently active to justify further evaluation of antitumour efficacy. Project Optimus emphasizes the importance of optimizing the dosage, considering not only the quantity of the drug administered but also the schedule. In the review, we identified 39 methods addressing schedule optimization. Some rely exclusively on safety data [140, 146, 147, 148], whereas many others are Phase I–II designs that incorporate both toxicity and efficacy information [149, 150, 151, 152, 153].

### 4.10 Leveraging External Data

The use of external data, typically incorporated into the prior distribution within a Bayesian framework, is infrequently addressed in the literature, being considered in less than 3% of the papers included in this scoping review. Various sources of external information may be leveraged, such as preclinical data [154, 155, 156], historical data from previous clinical trials [157, 158, 159], or data from adult populations to inform pediatric studies [160, 161]. Nevertheless, despite the limited “explicit” use of external data, many dose‐finding designs in the reviewed literature rely on Bayesian methodologies, in which prior distributions can, in principle, be calibrated to incorporate such historical information, even if this aspect is not formally detailed or reported in the publications.

### 4.11 Implementing Randomization Strategies

One key point of the 2023 FDA draft guidance is the need for comprehensive dose exploration early in development, including a formal comparison of two or more doses, ideally through randomization, to support dose selection. This randomization stage does not necessarily need to be powered for efficacy, but should be sufficiently sized to collect meaningful information at the doses of interest. Randomization can be performed *after escalation*, typically occurring in two‐stage Phase I/II designs: the first stage involves standard dose escalation to identify a set of acceptable doses in terms of safety or optimal candidate doses, while the second stage focuses on dose optimization through randomization. This optimization stage may use adaptive randomization based on efficacy among doses deemed acceptable in terms of toxicity, or fixed randomization across a restricted set of candidate doses. The candidate set of doses for optimization is typically the MTD identified in the first stage and one or two lower doses that demonstrate promising early efficacy or favorable PK/PD characteristics. Examples of two‐stage Phase I/II designs where randomization is performed after escalation include [108, 130, 162]. Two‐stage designs align with traditional phased development and allow different populations across stages, but they risk discarding the true optimal dose due to imprecise early estimates of toxicity and efficacy. Alternatively, randomization can be performed *during the escalation* phase, usually through an efficacy‐integrated Phase I/II design, in which both toxicity and efficacy data are used simultaneously to guide dose escalation and de‐escalation throughout the trial. The dose‐toxicity and dose–efficacy model estimates are continuously updated based on interim data to evaluate the benefit–risk trade‐off and make dose transition decisions. The next cohorts of patients are then allocated to admissible doses either by adaptive randomization based on efficacy [110], or by assignment to the dose with the highest estimated benefit–risk trade‐off. Efficacy‐integrated designs continuously update the optimal dose, enabling more efficient learning and allocation to promising doses, addressing the exploration–exploitation trade‐off [163]. However, these designs require timely observation of efficacy and toxicity. In our review, we globally identified 97 methods that incorporated randomization: 39 *during escalation*, 49 *after escalation*, and 9 applied to both. The Discussion section provides further commentary on these different approaches.

### 4.12 Software Availability

To assess the practical usability of published methods, we identified articles that provided implementation tools, such as code, R packages, applications, or software. Making innovative designs available through user‐friendly software is a key step toward broader adoption.

Figure 9 illustrates the evolution over time in the declared availability of such tools in articles proposing a new design, programming tool, benchmark or calibration method. Although many publications still do not provide access to any implementation material, the proportion of articles offering code, an R package, an application, or making them available upon request has increased in recent years—from approximately 30% in 2010 to around 50% currently. In the review, we also identified 15 papers specifically dedicated to the development of programming tools, such as R packages [164, 165, 166, 167, 168, 169, 170], applications [171, 172, 173], or code [174, 175, 176] to facilitate the implementation of dose‐finding. Although encouraging progress has been made in providing usable tools and improving reproducibility practices, such efforts are still not systematic.

**FIGURE 9:**
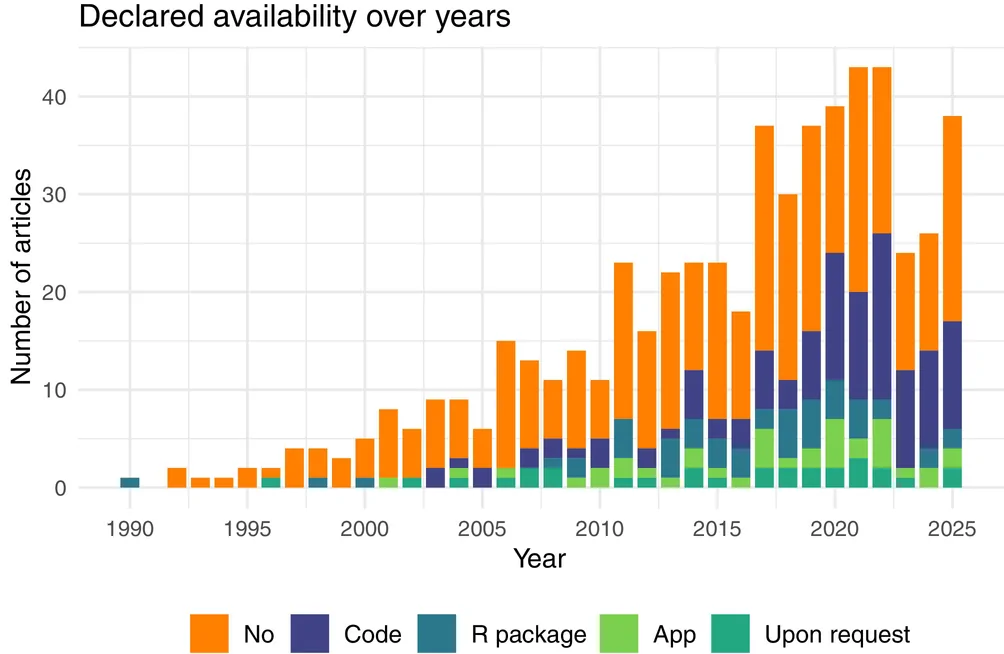
Tools available over the years, applied to ‘New design’, ‘Benchmark’, ‘Calibration’, and ‘Programming tool’ papers, and not applied to ‘Commentary’, ‘Review’, ‘Comparison’, ‘Theory’, or ‘Guidelines’ papers.

## 5 Limitations

This review has several limitations that should be acknowledged. First, the identified literature was predominantly drawn from statistical journals (96%), with only a small proportion of publications originating from clinical or medical journals (4%). This distribution reflects the methodological focus of the review, which targeted statistical designs for dose finding and dose optimization. It may, however, under‐represent areas in which methodological recommendations are currently emerging primarily within the clinical literature before being formalized into statistical frameworks. This is particularly relevant for patient‐reported outcomes (PROs), whose integration into early‐phase oncology trials is still at an early stage and may therefore be more frequently discussed in clinical journals than in the methodological literature captured here. As a result, our findings should be interpreted as a mapping of the published *methodological* landscape, which does not necessarily reflect the full extent of current clinical practice.

Second, the scope of this review was deliberately restricted to early‐phase dose‐escalation and dose‐optimization designs. Dose‐ranging studies (understood here as randomized, fixed‐dose parallel‐arm trials conducted independently of any prior dose‐escalation stage) were not explicitly included in the search strategy. This choice was motivated by the focus on first‐in‐human and early development settings; the dose‐optimization designs identified in this review, using randomization during or after escalation, remain incorporated into an initial dose‐escalation study and are therefore considered distinct from such dose‐ranging trials. However, it is worth noting that dose‐ranging studies are directly relevant to the broader dose‐optimization framework promoted by Project Optimus and may constitute an important complement to the methodological landscape described here.

Third, while the review aimed to provide a comprehensive and objective mapping of the methodological literature, the categorization of publications into predefined categories necessarily involves a degree of subjectivity. Some methodological approaches may be difficult to classify unambiguously, and the boundaries between categories, such as between utility‐based and efficacy‐driven designs, may not always be clearly defined in the original publications. Finally, although the review covers publications up to September 2025, the field is rapidly evolving, and new methodological contributions continue to emerge. In particular, methodological developments directly motivated by Project Optimus are still ongoing, and the present review may not fully capture the most recent advances in the field.

## 6 Discussion and Conclusion

The FDA's Project Optimus represents a major shift in the paradigm of dose recommendation in oncology drug development, seeking to reform dose optimization studies so that dose selection is supported by robust and comprehensive evidence. Dose optimization, as promoted by Project Optimus, should be viewed as a cumulative learning process rather than as a single decision made at one specific stage of development. Evidence may be progressively generated across multiple studies or integrated trial phases, including dose‐escalation cohorts, randomized dose‐expansion cohorts, PK/PD and exposure–response analyses, and sequential learning approaches. Although our review focused on individual published methods, we assessed whether these methods already incorporate some of the key principles of this broader dose‐optimization process. Several methodological proposals identified in our review use multi‐stage, adaptive, or seamless designs, illustrating how information collected over time can support progressive dose selection and refinement. In general, our review highlights that there is a rich methodological literature available to address many of the challenges raised by Project Optimus.

Despite the extensive development of model‐based methods in the statistical literature, their use in clinical practice remains limited. The traditional 3 + 3 design has historically been widely used, largely due to its simplicity, despite well‐documented shortcomings [1, 177, 178]. Although many alternative model‐based designs have been proposed, their adoption has been modest. In a review of 84 phase I trials of molecularly targeted agents conducted between 2000 and 2010, rule‐based designs were used in 91% of studies, whereas model‐based approaches accounted for only 8%; the standard 3 + 3 design alone was used in nearly half of all trials (49%) [179]. A subsequent review of oncology phase I combination trials conducted between 2011 and 2013 reported that traditional or modified 3 + 3 designs were used in 88% of studies [180]. More recently, a retrospective analysis of oncology phase I trials conducted between 2020 and 2022 found that rule‐based designs still represented 81.2% of dose‐escalation methods, with the traditional 3 + 3 design remaining dominant at 73.9%, while model‐assisted and model‐based approaches accounted for only 4.6% and 6.4% of trials, respectively [181]. A review of trial protocols published between 2017 and 2023 found that model‐based and model‐assisted designs were used in only 19% of studies [182]. More recent data collected following the launch of Project Optimus (2021–2024) indicate that the 3 + 3 and BOIN designs were the most commonly applied, used in 40% and 32% of protocols, respectively, with a declining use of 3 + 3 and increasing adoption of BOIN [10]. One possible reason for the limited adoption of some complex methodologies is the lack of available software code or user‐friendly applications. Many published designs are not accompanied by practical implementation tools, which may contribute to their limited use in practice. Many innovative designs have been evaluated primarily through simulation studies, and their comparative advantage over standard approaches has rarely been demonstrated under real‐world trial conditions, which may limit the confidence of clinical teams and sponsors in adopting them. Adoption may further be constrained by unfamiliarity of clinical investigators and data safety monitoring boards with innovative methodology. In addition, some methodologies that are theoretically appealing may face important operational challenges, such as the unavailability of data at the time of dose‐escalation decisions (e.g., delayed efficacy outcomes, PK measurements, or PRO data), which can limit their feasibility in real‐world trial settings. Ensuring that such data are collected, cleaned, and made accessible before each decision point requires substantial upfront organizational planning and close coordination across statisticians, data managers, and clinical teams.

The literature extensively covers efficacy‐informed dose determination, late‐onset outcomes, and combination therapies. While Project Optimus has renewed interest in dose optimization, the core principles, that is balancing risks and benefits, are long established and reflected in numerous Phase I/II designs [89, 90, 91, 92, 93, 96, 101, 102, 103, 104, 107, 108, 109, 110, 111, 112, 113, 114, 115, 116, 117, 118, 119, 120, 121, 122, 123]. As recommended in the FDA guidance, dose optimization should account for potential differences in required dosing across patients due to heterogeneity in tumor biology, patient characteristics, and treatment setting. In this review, we identified a substantial number of methods that explicitly incorporate patient heterogeneity into the dose‐finding process. However, important methodological gaps remain. Despite FDA encouragement, few frameworks exist for using PROs in dose optimization, and clear guidance on translating PRO data into dose recommendations is still lacking. Key challenges include defining clinically meaningful patient‐reported toxicity thresholds, analyzing small and heterogeneous subgroups, ensuring the availability of PRO data in real time during dose escalation, and addressing the subjective nature of patient‐reported information, which may introduce variability and affect consistency across patients. In addition, PRO collection may increase the time commitment required from patients in a trial and place additional demands on clinical teams. Although several recent methodological contributions have been proposed following the launch of the Project Optimus initiative, including approaches integrating PROs into dose‐finding designs [183, 184], the integration of PROs into formal dose‐finding methodologies remains a recent and still under‐explored research area. Similarly, although the FDA emphasizes that clinical PK/PD should be assessed throughout development to characterize dose– and exposure–response relationships, PK‐based dose‐finding designs remain limited. This may be partly due to practical constraints. PK measurements are often not available in real time during dose escalation, and ensuring the availability and timely analysis of PK samples at each new cohort remains a challenge. Moreover, population PK modeling requires a sufficient amount of PK data before it can be developed, which is rarely the case in the earliest cohorts. At early stages of drug development, the relationship between exposure, toxicity, and efficacy is often uncertain, which limits the added value of incorporating PK information into dose decision‐making. Finally, the inclusion of PK information for dose optimization requires close coordination between pharmacokinetic, clinical, and statistical teams, which can further increase operational complexity. Several extensions of this work could be considered. First, a dedicated systematic review of clinical trial protocols and published early‐phase oncology studies would allow a rigorous and quantitative assessment of which methodological approaches identified in the literature are actually adopted in practice, how their uptake has evolved over time, and whether the landscape has meaningfully shifted since the launch of Project Optimus. Such a review would complement the present work by bridging the gap between methodological development and real‐world implementation. Second, a position paper in which experts provide informed recommendations on the most appropriate early‐phase oncology trial designs, drawing on the findings of this scoping review, would represent a valuable extension of this work. Unlike the present review, such a paper would go beyond a neutral mapping of the literature to critically appraise the strengths and limitations of existing approaches, assess their practical feasibility and operational constraints, and offer contextualized guidance for their implementation across different clinical settings and trial objectives. Methodological innovation alone is insufficient to change current dose‐optimization practice; new designs must move beyond conceptual development to practical implementation. Progress also depends on clear guidelines, practical recommendations, and applied examples. Project Optimus has renewed interest in bridging this gap by encouraging the translation of innovative methods into practice, with guidance papers playing a key supporting role [74, 146, 185, 186, 187, 188, 189]. Easy‐to‐use tools are also a key component of the successful adoption of innovative designs, yet this remains an area requiring further development.

The FDA guidance provides a strong framework for improving dose‐optimization strategies, but translating its recommendations into practice remains challenging for sponsors. Regulatory familiarity and perceived acceptability are likely to influence design choice, and more practical guidance on which statistical methods may be appropriate for a given dose‐finding objective could therefore facilitate the uptake of these recommendations. The FDA fit‐for‐purpose designation of the BOIN design for maximum tolerated dose identification in 2021 [190] illustrates this point: it preceded the increasing uptake of this design reported in recent protocol reviews [10], although this trend cannot be disentangled from other factors, such as the availability of user‐friendly software. Similarly, early design‐specific dialog with regulators, facilitated by initiatives such as the FDA Model‐Informed Drug Development and Complex Innovative Trial Design Paired Meeting Programs, may help reduce the perceived regulatory risk associated with less familiar designs. Further work should also examine how the principles of Project Optimus have been reflected in regulatory assessment in recent years, in particular how the dose‐optimization strategies and supporting evidence submitted by sponsors have been evaluated, and how departures from the guidance recommendations have been discussed and resolved. Such an analysis would provide valuable insight into regulatory expectations and into the practical implementation of dose‐optimization principles. More broadly, this review shows that a rich and rapidly growing methodological toolbox is already available to address many of the challenges raised by Project Optimus. Continued collaboration between methodologists, sponsors and regulators should help bring these methods into routine practice and, ultimately, contribute to ensuring that patients receive dosages with the most favorable benefit–risk profile.

## Author Contributions

**Marie‐Karelle Riviere, Anaïs Andrillon, and Emma Gerard:** content expertise, conceptualization, methodology, data curation, writing, review, and editing of the final manuscript. **Moreno Ursino:** content expertise, conceptualization, methodology, data review, writing, review, and editing of the final manuscript. **Pavel Mozgunov:** content expertise, methodology, data review, writing, review, and editing of the final manuscript.

## Funding

The authors have nothing to report.

## Conflicts of Interest

The authors declare no conflicts of interest.

## Supporting information


**Supplementary Materials S1:** Methodology of the scoping review. List of the journals extracted and retained for the article search.
**Table 1:** List of statistical journals extracted from SCOPUS with subject area “Decision Sciences – Statistics, Probability and Uncertainty” and “Mathematics – Statistics and Probability”.
**Table 2:** List of additional medical journals publishing statistical methods added to the list of statistical journals.

**Supplementary Materials S2:** Scoping review protocols.

**Supplementary Materials S3:** List of articles with extracted features.

## Data Availability

Data sharing not applicable to this article as no datasets were generated or analyzed during the current study.
