# Supplementary material for: Evaluating Early‐Stage Oncology Clinical Trial Designs in the Era of Project Optimus: A Scoping Review
Source: Pharm Stat. 2026 Nov 5;25(6):e70131. doi: 10.1002/pst.70131 (PMC13740141; doi:10.1002/pst.70131)
Supplement: Supplementary file 1 [file PST-25-0-s001.pdf]

Supplementary materials S2 of "Evaluating early-stage oncology clinical trial designs in the era of project Optimus: a scoping review"

Scoping review protocols:

- Version 1
- Version 2
- Version 3

A scoping review of early phase clinical trials in the context of the Optimus Project for dose optimization in Oncology

Table of contents

1. Title ***2***
2. Registration ***2***
3. Authors ***2***
4. Contributions ***2***
5. Versions/Amendments ***2***
6. Support Sources/Sponsors ***2***
7. Rationale ***2***
8. Objectives ***3***
9. Eligibility criteria ***3***
10. Information sources ***3***
11. Search strategy ***3***
12. Study records ***4***
a. Data management ***4***
b. Selection process ***4***
c. Data charting process ***4***
13. Data items ***5***
14. Critical appraisal of individual sources of evidence ***6***
15. Synthesis of results ***6***

1. Title

Evaluating early-stage oncology clinical trial designs in the era of project Optimus: a scoping review

2. Registration

Not necessary for scoping review.

3. Authors

In alphabetic order:

- Anaïs Andrillon (AA), Saryga, Inserm ECSTRRA, anais.andrillon@saryga.com
- Emma Gerard (EG), Sanofi, emma.gerard@sanofi.com
- Pavel Mozgunov (PM), Cambridge University, pavel.mozgunov.ext@saryga.com
- Marie-Karelle Riviere (MKR), Saryga, marie-karelle.riviere@saryga.com
- Moreno Ursino (MU), Inserm-Inria HeKA, moreno.ursino@inserm.fr

4. Contributions

EG, MKR and AA: content expertise, conceptualization, methodology, data curation, writing, review and editing of final manuscript. MU: content expertise, conceptualization, methodology, data review, writing, review and editing of final manuscript. PM: content expertise, methodology, data review, writing, review and editing of final manuscript.

5. Versions/Amendments

**Version**	**Author(s)**	**Date**	**Comments**
V1	MU, EG, MKR, AA, PM	02/06/2023	Starting protocol

6. Support Sources/Sponsors

No sponsor.

7. Rationale

In recent years, oncology drug development has significantly shifted focus due to advances in targeted therapies, immunotherapies, and personalized medicine. Unlike traditional chemotherapy, these newer treatments interact with specific molecular targets or boost the immune system, leading to different dose-response relationships. Higher doses don't always mean better outcomes, especially when the effects can be nonlinear or non-monotonous. As a result, finding an "optimal dose" that balances effectiveness and safety has become increasingly important.
Launched in 2021 by the FDA's Oncology Center of Excellence, Project Optimus is an initiative aimed at modernizing how cancer drug doses are determined (FDA. 2021; Shah et al. 2021). Its main goal is to improve the process of identifying drug doses that are both effective and safe for patients, moving away from

outdated methods of dose selection. Project Optimus promotes a more refined approach to dose selection by considering individual patient responses, biomarkers, and pharmacokinetic and pharmacodynamic data. A central goal of the initiative is to encourage early and thorough evaluation of multiple dose levels during clinical trials. To support this, sponsors are urged to use randomized comparisons to reduce bias and better understand the relationships between dose, drug exposure, and treatment outcomes.
However, new trial designs continue to emerge each year from the research community, many of which aim to incorporate additional types of data, often in the form of novel endpoints, into early-phase studies. This growing complexity underscores the value of a scoping review to map the existing literature and identify areas that are well-studied versus those that remain underexplored in relation to the goals of the Project Optimus framework.

8. Objectives

The aim of this scoping review is to examine the literature on oncology trial design for early-phase clinical trials (i.e., Phase I, Phase II, and Phase I/II) in the context of the new Optimus project, and to answer the question: What has already been done, and what could be improved in light of the Optimus recommendations?

9. Eligibility criteria

For the journals:

- Statistical and mathematical journals published in English that accept submissions on biostatistical topics.
- Clinical journals published in English hat include or accept articles addressing biostatistical aspects.

For the articles:

- Studies published between January 1990 and September 2023 will be considered.
- All study designs are eligible, including quantitative, qualitative, and mixed-methods approaches.
- Full-text articles published in English.
- Only studies published in the pre-selected list of statistical and clinical journals will be included.
- Articles describing statistical methods, rather than applications only, and including a methodological investigation.

10. Information sources

PubMed search (Medline, Ovid) and SCOPUS (Elsevier).

11. Search strategy

First, a list of statistical journals to review will be established. For this purpose, from SCOPUS in "Source Type": the item "Journals" will be selected, and then in subject area: "Decision Sciences – Statistics, Probability and Uncertainty" and "Mathematics – Statistics and Probability" will be selected. In addition to the above, known clinical journals publishing statistical methods will be added to the list.

Second, a PubMed search (Medline, Ovid) and SCOPUS (Elsevier) search will be performed. The date of the last search will be approximately on September, 2023. Keywords used for the research are presented in the table below:

Phase 1 OR Phase I OR phase I-II OR phase I/II OR phase 1-2 OR phase 1/2 OR Dose finding OR Dose-finding	AND	Oncology OR Cancer OR Maximum tolerated dose Toxicity OR Dose escalation OR Dose-escalation

A combination of Medical Subject Headings (MeSH) terms and title, abstract, and keywords will be used to develop the initial Medline search, which will be validated against a known set of relevant studies. The Medline search will then be adapted to SCOPUS. In addition, records identified through manual screening of the reference lists of included articles and through the authors' knowledge, originating from medical or pharmacokinetics journals not covered by the main search, will be assessed for eligibility and included.

Pubmed search:
((“dose finding”[Title/Abstract]) OR (“dose-finding”[Title/Abstract]) OR (“phase I”[Title/Abstract]) OR (“phase 1”[Title/Abstract]) OR (“phase I/II”[Title/Abstract]) OR (“phase I-II”[Title/Abstract]) OR (“phase 1/2”[Title/Abstract]) OR (“phase 1-2”[Title/Abstract]))
AND
((“maximum tolerated dose”[Title/Abstract]) OR (“cancer”[Title/Abstract]) OR (“oncology”[Title/Abstract]) OR (“toxicity”[Title/Abstract]) OR (“dose escalation”[Title/Abstract]) OR (“dose-escalation”[Title/Abstract]))

Scopus search:
(TITLE-ABS-KEY ("Phase 1") OR TITLE-ABS-KEY ("Phase I") OR TITLE-ABS-KEY ("Phase I-II") OR TITLE-ABS-KEY ("Phase I/II") OR TITLE-ABS-KEY ("Phase 1-2") OR TITLE-ABS-KEY ("Phase 1/2") OR TITLE-ABS-KEY ("Dose finding") OR TITLE-ABS-KEY ("Dose-finding")) AND (TITLE-ABS-KEY ("Oncology") OR TITLE-ABS-KEY ("Cancer") OR TITLE-ABS-KEY ("Maximum tolerated dose") OR TITLE-ABS-KEY("Toxicity") OR TITLE-ABS-KEY ("Dose escalation") OR TITLE-ABS-KEY ("Dose-escalation"))

12. Study records

a. Data management

Data will be exported on Excel sheets. Cleaning and duplicates checking will be performed by AA and MKR.

b. Selection process

First step:
Three of the authors (MKR, EG, MU) will review the journals to retain them or not in the future search for statistical papers on dose-finding methods in oncology. If not all of the three authors agreed on a journal, after discussions a common decision will be taken.

Second step:
Four of the authors (MKR, EG, MU, AA, PM) will independently and randomly review the titles and abstracts of the articles retrieved. Each record will be assessed by at least two reviewers. In cases of disagreement, the reviewers will discuss the article to reach a consensus.

c. Data charting process

All authors (MU, EG, MKR, AA, PM) will pilot test the data extraction form on a few randomly selected papers to assess agreement and feasibility. Subsequently, the full list of articles will be divided among the five reviewers. In cases of uncertainty or disagreement, the paper will be discussed during the authors' meetings (held approximately twice a month) until consensus is reached.

13. Data items

Endpoint	Categories	Comment
Main purpose	Commentary / Review / New design / Benchmark / Comparison / Calibration / Theory / Programming tool / Guidelines	Main purpose of the paper.
Therapy	Monotherapy / Combination / Both	Developed for monotherapy, combination or both. Monotherapy means only one agent is being escalated, compared to combination where the dose levels of both agents are moving.
Toxicity endpoint	Binary / Ordinal / Continuous / Time-to-event / Quasi-binary / No	Type of toxicity endpoint. “No” in the exceptional case where toxicity is not used.
Multivariate toxicity	Yes / No	Incorporate multivariate patient toxicity information such as multiple grades.
Late toxicities	Yes / No	Handling DLTs occurring outside of a short-term evaluation window or accounting for toxicity information at multiple treatment cycles for the dose recommendation.
PRO	Yes / No	Considering patient reported outcome for dose recommendation.
Efficacy endpoint	Binary / Ordinal / Continuous / Time-to-event / Binary and continuous / No	Type of efficacy endpoint when the estimation of the recommended dose accounts for efficacy, otherwise “No”.
Multivariate efficacy	Yes / No	Incorporate multivariate patient efficacy information.
Late efficacies	Yes / No	Handling efficacy occurring outside of a short-term evaluation window.
Activity endpoint	Binary / Ordinal / Continuous / Time-to-event / No	Type of activity endpoint when the estimation of the optimal dose is based on biologic activity endpoint, otherwise “No”.
Multivariate activity	Yes / No	Incorporate multivariate patient activity information.
PK / PD Modelling	Yes / No	Incorporate pharmacokinetic and pharmacodynamic information for the dose recommendation.

Preclinical / Historical	Yes / No	Incorporate preclinical or historical information.
Decision criteria	Safety only / Efficacy-driven / Utility / NA	When only toxicity was used for dose recommendation, "Safety only" was selected. Otherwise, when either efficacy or activity endpoints were used, the recommended dose could be the dose with the highest utility ("Utility") or highest efficacy under toxicity constraints ("Efficacy-driven"). "NA" was used for Guidelines, Benchmark or Comparison papers.
Randomization	Yes / No	Randomization of patients performed between several dosages.
Comments randomization	During / After escalation	Patients can be randomized either during dose escalation or after a dose-escalation stage to identify acceptable dosages.
Schedules	Yes / No	Can account for different schedules in addition to dose level.
Heterogeneity	Yes / No	Allowing inclusion of covariates in the modeling or determining subgroup-specific recommended dose.
Design Type	Algorithm-based / Model-based / Model-assisted or Curve-free / Other / NA	Class of a design. "NA" applicable for Commentary, Benchmark or Programming tool papers.
Comments design type	Free text	Applicable only for "Other" design types.
Available tool	Yes / No / Upon request / NA	Tools available to perform the design. "NA" applicable for Commentary, Review, Comparison, Theory or Guidelines papers.
Comment tool	Code / R package / App / Upon request / NA	Type of tool available when "Yes" was selected for "Available tool".

14. Critical appraisal of individual sources of evidence

Not applicable.

15. Synthesis of results

The extracted data will be presented in table format and/or plots and we will provide a narrative summary of the studies by years, type and feature.

Bibliography

FDA. Project Optimus: reforming the dose optimization and dose selection paradigm in oncology. https://www.fda.gov/about-fda/oncology-center-excellence/project-optimus (2021). Accessed: 2023-02-01.

Shah, M., Rahman, A., Theoret, M. R., & Pazdur, R. (2021). The Drug-Dosing Conundrum in Oncology - When Less Is More. The New England journal of medicine, 385(16), 1445–1447. https://doi.org/10.1056/NEJMp2109826

A scoping review of early phase clinical trials in the context of the Optimus Project for dose optimization in Oncology

Table of contents

1. *Title* ... 2
2. *Registration* ... 2
3. *Authors* ... 2
4. *Contributions* ... 2
5. *Versions/Amendments* ... 2
6. *Support Sources/Sponsors* ... 2
7. *Rationale* ... 2
8. *Objectives* ... 3
9. *Eligibility criteria* ... 3
10. *Information sources* ... 3
11. *Search strategy* ... 3
12. *Study records* ... 7
a. *Data management* ... 7
b. *Selection process* ... 7
c. *Data charting process* ... 7
13. *Data items* ... 7
14. *Critical appraisal of individual sources of evidence* ... 9
15. *Synthesis of results* ... 9

1. Title

Evaluating early-stage oncology clinical trial designs in the era of project Optimus: a scoping review

2. Registration

Not necessary for scoping review.

3. Authors

In alphabetic order:

- Anaïs Andrillon (AA), Saryga, Inserm ECSTRRA, anais.andrillon@saryga.com
- Emma Gerard (EG), Sanofi, emma.gerard@sanofi.com
- Pavel Mozgunov (PM), Cambridge University, pavel.mozgunov.ext@saryga.com
- Marie-Karelle Riviere (MKR), Saryga, marie-karelle.riviere@saryga.com
- Moreno Ursino (MU), Inserm-Inria HeKA, moreno.ursino@inserm.fr

4. Contributions

EG, MKR and AA: content expertise, conceptualization, methodology, data curation, writing, review and editing of final manuscript. MU: content expertise, conceptualization, methodology, data review, writing, review and editing of final manuscript. PM: content expertise, methodology, data review, writing, review and editing of final manuscript.

5. Versions/Amendments

**Version**	**Author(s)**	**Date**	**Comments**
V1	MU, EG, MKR, AA, PM	02/06/2023	Starting protocol
V2	MU	13/09/2023	Including information on the selected journals on the search strategies

6. Support Sources/Sponsors

No sponsor.

7. Rationale

In recent years, oncology drug development has significantly shifted focus due to advances in targeted therapies, immunotherapies, and personalized medicine. Unlike traditional chemotherapy, these newer treatments interact with specific molecular targets or boost the immune system, leading to different dose-response relationships. Higher doses don't always mean better outcomes, especially when the effects can be nonlinear or non-monotonous. As a result, finding an "optimal dose" that balances effectiveness and safety has become increasingly important.

Launched in 2021 by the FDA's Oncology Center of Excellence, Project Optimus is an initiative aimed at modernizing how cancer drug doses are determined (FDA. 2021; Shah et al. 2021). Its main goal is to improve the process of identifying drug doses that are both effective and safe for patients, moving away from outdated methods of dose selection. Project Optimus promotes a more refined approach to dose selection by considering individual patient responses, biomarkers, and pharmacokinetic and pharmacodynamic data. A central goal of the initiative is to encourage early and thorough evaluation of multiple dose levels during clinical trials. To support this, sponsors are urged to use randomized comparisons to reduce bias and better understand the relationships between dose, drug exposure, and treatment outcomes.
However, new trial designs continue to emerge each year from the research community, many of which aim to incorporate additional types of data, often in the form of novel endpoints, into early-phase studies. This growing complexity underscores the value of a scoping review to map the existing literature and identify areas that are well-studied versus those that remain underexplored in relation to the goals of the Project Optimus framework.

8. Objectives

The aim of this scoping review is to examine the literature on oncology trial design for early-phase clinical trials (i.e., Phase I, Phase II, and Phase I/II) in the context of the new Optimus project, and to answer the question: What has already been done, and what could be improved in light of the Optimus recommendations?

9. Eligibility criteria

For the journals:

- Statistical and mathematical journals published in English that accept submissions on biostatistical topics.
- Clinical journals published in English hat include or accept articles addressing biostatistical aspects.

For the articles:

- Studies published between January 1990 and September 2023 will be considered.
- All study designs are eligible, including quantitative, qualitative, and mixed-methods approaches.
- Full-text articles published in English.
- Only studies published in the pre-selected list of statistical and clinical journals will be included.
- Articles describing statistical methods, rather than applications only, and including a methodological investigation.

10. Information sources

PubMed search (Medline, Ovid) and SCOPUS (Elsevier).

11. Search strategy

First, a list of statistical journals to review will be established. For this purpose, from SCOPUS in "Source Type": the item "Journals" will be selected, and then in subject area: "Decision Sciences – Statistics, Probability and Uncertainty" and "Mathematics – Statistics and Probability" will be selected. In addition to the above, known clinical journals publishing statistical methods will be added to the list.

Second, a PubMed search (Medline, Ovid) and SCOPUS (Elsevier) search will be performed. The date of the last search will be approximately on September, 2023. Keywords used for the research are presented in the table below:

Phase 1 OR Phase I OR phase I-II OR phase I/II OR phase 1-2 OR phase 1/2 OR Dose finding OR Dose-finding	AND	Oncology OR Cancer OR Maximum tolerated dose Toxicity OR Dose escalation OR Dose-escalation

A combination of Medical Subject Headings (MeSH) terms and title, abstract, and keywords will be used to develop the initial Medline search, which will be validated against a known set of relevant studies. The Medline search will then be adapted to SCOPUS. In addition, records identified through manual screening of the reference lists of included articles and through the authors' knowledge, originating from medical or pharmacokinetics journals not covered by the main search, will be assessed for eligibility and included.

In the following, the detailed searches after journals selections.
Pubmed search:
(("Adv Theory Simul"[Journal]) OR ("Adv Appl Probab"[Journal]) OR ("Adv Data Anal Classif"[Journal]) OR ("Am Stat"[Journal]) OR ("Ann Appl Probab"[Journal]) OR ("Ann Appl Stat"[Journal]) OR ("Ann Math Artif Intell"[Journal]) OR ("Ann Probab"[Journal]) OR ("Ann Stat"[Journal]) OR ("Ann Inst Stat Math"[Journal]) OR ("Annu Rev Stat Appl"[Journal]) OR ("Adv Stat Anal"[Journal]) OR ("Bayesian Anal"[Journal]) OR ("Bernoulli (Andover)"[Journal]) OR ("Biom J"[Journal]) OR ("Biometrics"[Journal]) OR ("Biometrika"[Journal]) OR ("Biostatistics"[Journal]) OR ("Can J Stat"[Journal]) OR ("Chil J Stat"[Journal]) OR ("Commun Stat Appl Methods"[Journal]) OR ("Commun Math Stat"[Journal]) OR ("Commun Stat Simul Comput"[Journal]) OR ("Commun Stat Theory Methods"[Journal]) OR ("Commun Stat Case Stud Data Anal Appl"[Journal]) OR ("Comput Stat"[Journal]) OR ("Comput Stat Data Anal"[Journal]) OR ("Electron J Probab"[Journal]) OR ("Electron J Stat"[Journal]) OR ("ESAIM Probab Stat"[Journal]) OR ("Front Appl Math Stat"[Journal]) OR ("J Math Stat"[Journal]) OR ("Inf inference"[Journal]) OR ("Int J Uncertain Quantif"[Journal]) OR ("Int J Biostat"[Journal]) OR ("Int J Math Comput Sci"[Journal]) OR ("Int J Stat Med Res"[Journal]) OR ("Int Stat Rev"[Journal]) OR ("Jpn J Stat Data Sci"[Journal]) OR ("J Appl Probab"[Journal]) OR ("J Appl Stat"[Journal]) OR ("J Biopharm Stat"[Journal]) OR ("J Mach Learn Res"[Journal]) OR ("J Mod Appl Stat Methods"[Journal]) OR ("J Multivar Anal"[Journal]) OR ("J Nonparametr Stat"[Journal]) OR ("J Off Stat"[Journal]) OR ("J Stat Comput Simul"[Journal]) OR ("J Stat Distrib Appl"[Journal]) OR ("J Stat Plan Inference"[Journal]) OR ("J Stat Softw"[Journal]) OR ("J Stat Theory Appl"[Journal]) OR ("J Stat Theory Pract"[Journal]) OR ("J Surv Stat Methodol"[Journal]) OR ("J Am Stat Assoc"[Journal]) OR ("JIRSS"[Journal]) OR ("J Korean Stat Soc"[Journal]) OR ("J Theor Probab"[Journal]) OR ("Lett Biomath"[Journal]) OR ("Math Biosci"[Journal]) OR ("Metrika"[Journal]) OR ("Metron"[Journal]) OR ("Model Assist Stat Appl"[Journal]) OR ("Monte Carlo Methods Appl"[Journal]) OR ("Pharm Stat"[Journal]) OR ("Revstat Stat J"[Journal]) OR ("Sankhya Ser A"[Journal]) OR ("Sankhya B (2008)"[Journal]) OR ("Sankhya"[Journal]) OR ("Scand Stat Theory Appl"[Journal]) OR ("Seq Anal"[Journal]) OR ("J Comput Math"[Journal]) OR ("Sort (Barc)"[Journal]) OR ("S Afr Stat J"[Journal]) OR ("Stat Neerl"[Journal]) OR ("Stat Sin"[Journal]) OR ("Stat Methods Med Res"[Journal]) OR ("Stat Modelling"[Journal]) OR ("Stat Pap (Berl)"[Journal]) OR ("J Stat Sci Appl"[Journal]) OR ("Stat Theory Relat Fields"[Journal]) OR ("Stat Comput"[Journal]) OR ("Stat Interface"[Journal]) OR ("Stat Probab Lett"[Journal]) OR ("Stat Biopharm Res"[Journal]) OR ("Stat Biosci"[Journal]) OR ("Stat Med"[Journal]) OR ("R J"[Journal]) OR ("Stata J"[Journal]) OR ("Wiley Interdiscip Rev Comput Stat"[Journal]))
AND
((“dose finding”[Title/Abstract]) OR (“dose-finding”[Title/Abstract]) OR (“phase I”[Title/Abstract]) OR (“phase 1”[Title/Abstract]) OR (“phase I/II”[Title/Abstract]) OR (“phase I-II”[Title/Abstract]) OR (“phase 1/2”[Title/Abstract]) OR (“phase 1-2”[Title/Abstract]))
AND
((“maximum tolerated dose”[Title/Abstract]) OR (“cancer”[Title/Abstract]) OR (“oncology”[Title/Abstract]) OR (“toxicity”[Title/Abstract]) OR (“dose escalation”[Title/Abstract]) OR (“dose -escalation”[Title/Abstract]))

(("Clin Trials"[Journal]) OR ("BMC Med Res Methodol"[Journal]) OR ("Contemp Clin Trials"[Journal]) OR ("Contemp Clin Trials Commun"[Journal]) OR ("Comput Methods Programs Biomed"[Journal]) OR ("Control Clin Trials"[Journal]))
AND (("dose finding"[Title/Abstract]) OR ("dose-finding"[Title/Abstract]) OR ("phase I"[Title/Abstract]) OR ("phase 1"[Title/Abstract]) OR ("phase I/II"[Title/Abstract]) OR ("phase I-II"[Title/Abstract]) OR ("phase 1/2"[Title/Abstract]) OR ("phase 1-2"[Title/Abstract]))
AND (("maximum tolerated dose"[Title/Abstract]) OR ("cancer"[Title/Abstract]) OR ("oncology"[Title/Abstract]) OR ("toxicity"[Title/Abstract]) OR ("dose escalation"[Title/Abstract]) OR ("dose-escalation"[Title/Abstract]))

Scopus search:
(TITLE-ABS-KEY ("Phase 1") OR TITLE-ABS-KEY ("Phase I") OR TITLE-ABS-KEY ("Phase I-II") OR TITLE-ABS-KEY ("Phase I/II") OR TITLE-ABS-KEY ("Phase 1-2") OR TITLE-ABS-KEY ("Phase 1/2") OR TITLE-ABS-KEY ("Dose finding") OR TITLE-ABS-KEY ("Dose-finding")) AND (TITLE-ABS-KEY ("Oncology") OR TITLE-ABS-KEY ("Cancer") OR TITLE-ABS-KEY ("Maximum tolerated dose") OR TITLE-ABS-KEY("Toxicity") OR TITLE-ABS-KEY ("Dose escalation") OR TITLE-ABS-KEY ("Dose-escalation")) AND (SRCTITLE('Advanced Mathematical Models and Applications') OR SRCTITLE('Advanced Theory and Simulations') OR SRCTITLE('Advances in Applied Probability') OR SRCTITLE('Advances in Data Analysis and Classification') OR SRCTITLE('Advances in Decision Sciences') OR SRCTITLE('Alea') OR SRCTITLE('American Statistician') OR SRCTITLE('Annales de l'institut Henri Poincare B Probability and Statistics') OR SRCTITLE('Annals of Applied Probability') OR SRCTITLE('Annals of Applied Statistics') OR SRCTITLE('Annals of Data Science') OR SRCTITLE('Annals of Mathematics') OR SRCTITLE('Annals of Probability') OR SRCTITLE('Annals of Statistics') OR SRCTITLE('Annals of the Institute of Statistical Mathematics') OR SRCTITLE('Annual Review of Statistics and Its Application') OR SRCTITLE('AStA Advances in Statistical Analysis') OR SRCTITLE('Australian and New Zealand Journal of Statistics') OR SRCTITLE('Australian Journal of Statistics') OR SRCTITLE('Austrian Journal of Statistics') OR SRCTITLE('Bayesian Analysis') OR SRCTITLE('Bernoulli') OR SRCTITLE('Bioinformatics') OR SRCTITLE('Biometrical Journal') OR SRCTITLE('Biometrics') OR SRCTITLE('Biometrika') OR SRCTITLE('Biostatistics') OR SRCTITLE('Brazilian Journal of Probability and Statistics') OR SRCTITLE('Calcutta Statistical Association Bulletin') OR SRCTITLE('Canadian Journal of Statistics') OR SRCTITLE('Chilean Journal of Statistics') OR SRCTITLE('Communications for Statistical Applications and Methods') OR SRCTITLE('Communications in Mathematics and Statistics') OR SRCTITLE('Communications in Statistics') OR SRCTITLE('Communications in Statistics - Simulation and Computation') OR SRCTITLE('Communications in Statistics - Theory and Methods') OR SRCTITLE('Communications in Statistics Case Studies Data Analysis and Applications') OR SRCTITLE('Communications in Statistics Part B: Simulation and Computation') OR SRCTITLE('Communications in Statistics. Part C: Sequential Analysis') OR SRCTITLE('Communications on Stochastic Analysis') OR SRCTITLE('Computational Statistics') OR SRCTITLE('Computational Statistics & Data Analysis') OR SRCTITLE('Electronic Communications in Probability') OR SRCTITLE('Electronic Journal of Applied Statistical Analysis') OR SRCTITLE('Electronic Journal of Probability') OR SRCTITLE('Electronic Journal of Statistics') OR SRCTITLE('ESAIM - Probability and Statistics') OR SRCTITLE('European Journal of Pure and Applied Mathematics') OR SRCTITLE('Frontiers in Applied Mathematics and Statistics') OR SRCTITLE('Hacettepe Journal of Mathematics and Statistics') OR SRCTITLE('Information and Inference') OR SRCTITLE('International Journal for Uncertainty Quantification') OR SRCTITLE('International Journal of Biostatistics') OR SRCTITLE('International Journal of Mathematics and Computer Science') OR SRCTITLE('International Journal of Statistics in Medical Research') OR SRCTITLE('International Statistical Review') OR SRCTITLE('Japanese Journal of Statistics and Data Science') OR SRCTITLE('Jordan Journal of Mathematics and Statistics') OR SRCTITLE('Journal of Applied Mathematics and Decision Sciences') OR SRCTITLE('Journal of Applied Mathematics and Simulation') OR SRCTITLE('Journal of Applied Probability') OR SRCTITLE('Journal of Applied Probability and Statistics') OR SRCTITLE('Journal of Applied Statistical Science') OR SRCTITLE('Journal of Applied Statistics') OR SRCTITLE('Journal of Biopharmaceutical Statistics') OR SRCTITLE('Journal of Biostatistics and Epidemiology') OR SRCTITLE('Journal of Machine Learning Research') OR SRCTITLE('Journal of Mathematical Sciences') OR SRCTITLE('Journal of Mathematics and Statistics') OR SRCTITLE('Journal of Mixed Methods Research') OR SRCTITLE('Journal of Modern Applied Statistical Methods') OR SRCTITLE('Journal of Multivariate Analysis') OR SRCTITLE('Journal of Nonparametric

Statistics') OR SRCTITLE('Journal of Official Statistics') OR SRCTITLE('Journal of Probability and Statistics') OR SRCTITLE('Journal of Reliability and Statistical Studies') OR SRCTITLE('Journal of Statistical Computation and Simulation') OR SRCTITLE('Journal of Statistical Distributions and Applications') OR SRCTITLE('Journal of Statistical Planning and Inference') OR SRCTITLE('Journal of Statistical Software') OR SRCTITLE('Journal of Statistical Theory and Applications') OR SRCTITLE('Journal of Statistical Theory and Practice') OR SRCTITLE('Journal of Statistics Applications and Probability') OR SRCTITLE('Journal of Survey Statistics and Methodology') OR SRCTITLE('Journal of the American Statistical Association') OR SRCTITLE('Journal of the Iranian Statistical Society') OR SRCTITLE('Journal of the Italian Statistical Society') OR SRCTITLE('Journal of the Korean Statistical Society') OR SRCTITLE('Journal of the Royal Statistical Society Series D: The Statistician') OR SRCTITLE('Journal of the Royal Statistical Society. Series A: Statistics in Society') OR SRCTITLE('Journal of the Royal Statistical Society. Series B: Methodological') OR SRCTITLE('Journal of the Royal Statistical Society. Series B: Statistical Methodology') OR SRCTITLE('Journal of the Royal Statistical Society. Series C: Applied Statistics') OR SRCTITLE('Journal of Theoretical Probability') OR SRCTITLE('Letters in Biomathematics') OR SRCTITLE('Mathematical Biosciences') OR SRCTITLE('Mathematical Methods of Statistics') OR SRCTITLE('Mathematical Sciences') OR SRCTITLE('Mathematical Statistician and Engineering Applications') OR SRCTITLE('Mathematics and Statistics') OR SRCTITLE('Methods, Data, Analyses') OR SRCTITLE('Metrika') OR SRCTITLE('Metron') OR SRCTITLE('Model Assisted Statistics and Applications') OR SRCTITLE('Monte Carlo Methods and Applications') OR SRCTITLE('New Zealand Statistician') OR SRCTITLE('Pakistan Journal of Statistics') OR SRCTITLE('Pakistan Journal of Statistics and Operation Research') OR SRCTITLE('Pharmaceutical Statistics') OR SRCTITLE('Probability and Mathematical Statistics') OR SRCTITLE('Publications of the American Statistical Association') OR SRCTITLE('Quarterly Publications of the American Statistical Association') OR SRCTITLE('R Journal') OR SRCTITLE('Researches in Mathematics') OR SRCTITLE('Revista Brasileira de Biometria') OR SRCTITLE('Revstat Statistical Journal') OR SRCTITLE('Sankhya A') OR SRCTITLE('Sankhya B') OR SRCTITLE('Sankhya: The Indian Journal of Statistics') OR SRCTITLE('Sao Paulo Journal of Mathematical Sciences') OR SRCTITLE('Scandinavian Journal of Statistics') OR SRCTITLE('Sequential Analysis') OR SRCTITLE('SMAI Journal of Computational Mathematics') OR SRCTITLE('SORT') OR SRCTITLE('South African Statistical Journal') OR SRCTITLE('Stat') OR SRCTITLE('Statistica Applicata') OR SRCTITLE('Statistica e Applicazioni') OR SRCTITLE('Statistica Neerlandica') OR SRCTITLE('Statistica Sinica') OR SRCTITLE('Statistical Methodology') OR SRCTITLE('Statistical Methods and Applications') OR SRCTITLE('Statistical Methods in Medical Research') OR SRCTITLE('Statistical Modelling') OR SRCTITLE('Statistical Papers') OR SRCTITLE('Statistical Science') OR SRCTITLE('Statistical Theory and Related Fields') OR SRCTITLE('Statistics') OR SRCTITLE('Statistics and Computing') OR SRCTITLE('Statistics and its Interface') OR SRCTITLE('Statistics and Probability Letters') OR SRCTITLE('Statistics in Biopharmaceutical Research') OR SRCTITLE('Statistics in Biosciences') OR SRCTITLE('Statistics in Medicine') OR SRCTITLE('Statistics, Optimization and Information Computing') OR SRCTITLE('Statistika') OR SRCTITLE('Test') OR SRCTITLE('Thailand Statistician') OR SRCTITLE('The R Journal') OR SRCTITLE('The Stata Journal') OR SRCTITLE('Theory of Probability and its Applications') OR SRCTITLE('Theory of Probability and Mathematical Statistics') OR SRCTITLE('Turkish World Mathematical Society Journal of Applied and Engineering Mathematics') OR SRCTITLE('Wiley Interdisciplinary Reviews: Computational Statistics'))

(TITLE-ABS-KEY ("Phase 1") OR TITLE-ABS-KEY ("Phase I") OR TITLE-ABS-KEY ("Phase I-II") OR TITLE-ABS-KEY ("Phase I/II") OR TITLE-ABS-KEY ("Phase 1-2") OR TITLE-ABS-KEY ("Phase 1/2") OR TITLE-ABS-KEY ("Dose finding") OR TITLE-ABS-KEY ("Dose-finding")) AND (TITLE-ABS-KEY ("Oncology") OR TITLE-ABS-KEY ("Cancer") OR TITLE-ABS-KEY ("Maximum tolerated dose") OR TITLE-ABS-KEY ("Toxicity") OR TITLE-ABS-KEY ("Dose escalation") OR TITLE-ABS-KEY ("Dose-escalation")) AND (SRCTITLE ('bmc AND medical AND research AND methodology') OR SRCTITLE ('contemporary AND clinical AND trials AND communications') OR SRCTITLE ('computer AND methods AND programs AND in AND biomedicine') OR SRCTITLE ('controlled AND clinical AND trials') OR SRCTITLE ('clinical AND trials') OR SRCTITLE ('contemporary AND clinical AND trials')) AND (LIMIT-TO (EXACTSRCTITLE , "BMC Medical Research Methodology") OR LIMIT-TO (EXACTSRCTITLE , "Clinical Trials") OR LIMIT-TO (EXACTSRCTITLE , "Computer Methods And Programs In Biomedicine") OR

LIMIT-TO (EXACTSRCTITLE , "Contemporary Clinical Trials") OR LIMIT-TO (EXACTSRCTITLE , "Contemporary Clinical Trials Communications") OR LIMIT-TO (EXACTSRCTITLE , "Controlled Clinical Trials"))

12. Study records

a. Data management

Data will be exported on Excel sheets. Cleaning and duplicates checking will be performed by AA and MKR.

b. Selection process

First step:
Three of the authors (MKR, EG, MU) will review the journals to retain them or not in the future search for statistical papers on dose-finding methods in oncology. If not all of the three authors agreed on a journal, after discussions a common decision will be taken.

Second step:
Four of the authors (MKR, EG, MU, AA, PM) will independently and randomly review the titles and abstracts of the articles retrieved. Each record will be assessed by at least two reviewers. In cases of disagreement, the reviewers will discuss the article to reach a consensus.

c. Data charting process

All authors (MU, EG, MKR, AA, PM) will pilot test the data extraction form on a few randomly selected papers to assess agreement and feasibility. Subsequently, the full list of articles will be divided among the five reviewers. In cases of uncertainty or disagreement, the paper will be discussed during the authors' meetings (held approximately twice a month) until consensus is reached.

13. Data items

Endpoint	Categories	Comment
Main purpose	Commentary / Review / New design / Benchmark / Comparison / Calibration / Theory / Programming tool / Guidelines	Main purpose of the paper.
Therapy	Monotherapy / Combination / Both	Developed for monotherapy, combination or both. Monotherapy means only one agent is being escalated, compared to combination where the dose levels of both agents are moving.
Toxicity endpoint	Binary / Ordinal / Continuous / Time-to-event / Quasi-binary / No	Type of toxicity endpoint. "No" in the exceptional case where toxicity is not used.
Multivariate toxicity	Yes / No	Incorporate multivariate patient toxicity information such as multiple grades.

Late toxicities	Yes / No	Handling DLTs occurring outside of a short-term evaluation window or accounting for toxicity information at multiple treatment cycles for the dose recommendation.
PRO	Yes / No	Considering patient reported outcome for dose recommendation.
Efficacy endpoint	Binary / Ordinal / Continuous / Time-to-event / Binary and continuous / No	Type of efficacy endpoint when the estimation of the recommended dose accounts for efficacy, otherwise “No”.
Multivariate efficacy	Yes / No	Incorporate multivariate patient efficacy information.
Late efficacies	Yes / No	Handling efficacy occurring outside of a short-term evaluation window.
Activity endpoint	Binary / Ordinal / Continuous / Time-to-event / No	Type of activity endpoint when the estimation of the optimal dose is based on biologic activity endpoint, otherwise “No”.
Multivariate activity	Yes / No	Incorporate multivariate patient activity information.
PK / PD Modelling	Yes / No	Incorporate pharmacokinetic and pharmacodynamic information for the dose recommendation.
Preclinical / Historical	Yes / No	Incorporate preclinical or historical information.
Decision criteria	Safety only / Efficacy-driven / Utility / NA	When only toxicity was used for dose recommendation, “Safety only” was selected. Otherwise, when either efficacy or activity endpoints were used, the recommended dose could be the dose with the highest utility (“Utility”) or highest efficacy under toxicity constraints (“Efficacy-driven”). “NA” was used for Guidelines, Benchmark or Comparison papers.
Randomization	Yes / No	Randomization of patients performed between several dosages.
Comments randomization	During / After escalation	Patients can be randomized either during dose escalation or after a dose-escalation stage to identify acceptable dosages.

Schedules	Yes / No	Can account for different schedules in addition to dose level.
Heterogeneity	Yes / No	Allowing inclusion of covariates in the modeling or determining subgroup-specific recommended dose.
Design Type	Algorithm-based / Model-based / Model-assisted or Curve-free / Other / NA	Class of a design. “NA” applicable for Commentary, Benchmark or Programming tool papers.
Comments design type	Free text	Applicable only for “Other” design types.
Available tool	Yes / No / Upon request / NA	Tools available to perform the design. “NA” applicable for Commentary, Review, Comparison, Theory or Guidelines papers.
Comment tool	Code / R package / App / Upon request / NA	Type of tool available when “Yes” was selected for “Available tool”.

14. Critical appraisal of individual sources of evidence

Not applicable.

15. Synthesis of results

The extracted data will be presented in table format and/or plots and we will provide a narrative summary of the studies by years, type and feature.

Bibliography

FDA. Project Optimus: reforming the dose optimization and dose selection paradigm in oncology. https://www.fda.gov/about-fda/oncology-center-excellence/project-optimus (2021). Accessed: 2023-02-01.

Shah, M., Rahman, A., Theoret, M. R., & Pazdur, R. (2021). The Drug-Dosing Conundrum in Oncology - When Less Is More. The New England journal of medicine, 385(16), 1445–1447. https://doi.org/10.1056/NEJMp2109826

A scoping review of early phase clinical trials in the context of the Optimus Project for dose optimization in Oncology

Table of contents

1. Title... 2
2. Registration 2
3. Authors 2
4. Contributions 2
5. Versions/Amendments 2
6. Support Sources/Sponsors 2
7. Rationale 2
8. Objectives 3
9. Eligibility criteria 3
10. Information sources.. 3
11. Search strategy 3
12. Study records.. 7
a. Data management 7
b. Selection process.. 7
c. Data charting process 7
13. Data items... 7
14. Critical appraisal of individual sources of evidence 9
15. Synthesis of results 9

This template is based on a systematic review protocol template originally developed by Helena VonVille, MLS, MPH.

1. Title

Evaluating early-stage oncology clinical trial designs in the era of project Optimus: a scoping review

2. Registration

Not necessary for scoping review.

3. Authors

In alphabetic order:

- Anaïs Andrillon (AA), Saryga, Inserm ECSTRRA, anais.andrillon@saryga.com
- Emma Gerard (EG), Sanofi, emma.gerard@sanofi.com
- Pavel Mozgunov (PM), Cambridge University, pavel.mozgunov.ext@saryga.com
- Marie-Karelle Riviere (MKR), Saryga, marie-karelle.riviere@saryga.com
- Moreno Ursino (MU), Inserm-Inria HeKA, moreno.ursino@inserm.fr

4. Contributions

EG, MKR and AA: content expertise, conceptualization, methodology, data curation, writing, review and editing of final manuscript. MU: content expertise, conceptualization, methodology, data review, writing, review and editing of final manuscript. PM: content expertise, methodology, data review, writing, review and editing of final manuscript.

5. Versions/Amendments

Version	Author(s)	Date	Comments
V1	MU, EG, MKR, AA, PM	02/06/2023	Starting protocol
V2	MU	13/09/2023	Including information on the selected journals on the search strategies
V3	MU	15/10/2025	Extension of the search period (in bold in the text)

6. Support Sources/Sponsors

No sponsor.

7. Rationale

In recent years, oncology drug development has significantly shifted focus due to advances in targeted therapies, immunotherapies, and personalized medicine. Unlike traditional chemotherapy, these newer treatments interact with specific molecular targets or boost the immune system, leading to different dose-response relationships. Higher doses don't always mean better outcomes, especially when the effects can be

nonlinear or non-monotonous. As a result, finding an “optimal dose” that balances effectiveness and safety has become increasingly important.
Launched in 2021 by the FDA’s Oncology Center of Excellence, Project Optimus is an initiative aimed at modernizing how cancer drug doses are determined (FDA. 2021; Shah et al. 2021). Its main goal is to improve the process of identifying drug doses that are both effective and safe for patients, moving away from outdated methods of dose selection. Project Optimus promotes a more refined approach to dose selection by considering individual patient responses, biomarkers, and pharmacokinetic and pharmacodynamic data. A central goal of the initiative is to encourage early and thorough evaluation of multiple dose levels during clinical trials. To support this, sponsors are urged to use randomized comparisons to reduce bias and better understand the relationships between dose, drug exposure, and treatment outcomes.
However, new trial designs continue to emerge each year from the research community, many of which aim to incorporate additional types of data, often in the form of novel endpoints, into early-phase studies. This growing complexity underscores the value of a scoping review to map the existing literature and identify areas that are well-studied versus those that remain underexplored in relation to the goals of the Project Optimus framework.

8. Objectives

The aim of this scoping review is to examine the literature on oncology trial design for early-phase clinical trials (i.e., Phase I, Phase II, and Phase I/II) in the context of the new Optimus project, and to answer the question: What has already been done, and what could be improved in light of the Optimus recommendations?

9. Eligibility criteria

For the journals:

- Statistical and mathematical journals published in English that accept submissions on biostatistical topics.
- Clinical journals published in English hat include or accept articles addressing biostatistical aspects.

For the articles:

- Studies published between January 1990 and **September 2025** will be considered.
- All study designs are eligible, including quantitative, qualitative, and mixed-methods approaches.
- Full-text articles published in English.
- Only studies published in the pre-selected list of statistical and clinical journals will be included.
- Articles describing statistical methods, rather than applications only, and including a methodological investigation.

10. Information sources

PubMed search (Medline, Ovid) and SCOPUS (Elsevier).

11. Search strategy

First, a list of statistical journals to review will be established. For this purpose, from SCOPUS in “Source Type”: the item “Journals” will be selected, and then in subject area: “Decision Sciences – Statistics, Probability and Uncertainty” and “Mathematics – Statistics and Probability” will be selected. In addition to the above, known clinical journals publishing statistical methods will be added to the list.

Second, a PubMed search (Medline, Ovid) and SCOPUS (Elsevier) search will be performed. The date of the last search will be approximately on September, 2023. Keywords used for the research are presented in the table below:

Phase 1 OR Phase I OR phase I-II OR phase I/II OR phase 1-2 OR phase 1/2 OR Dose finding OR Dose-finding	AND	Oncology OR Cancer OR Maximum tolerated dose Toxicity OR Dose escalation OR Dose-escalation

A combination of Medical Subject Headings (MeSH) terms and title, abstract, and keywords will be used to develop the initial Medline search, which will be validated against a known set of relevant studies. The Medline search will then be adapted to SCOPUS. In addition, records identified through manual screening of the reference lists of included articles and through the authors' knowledge, originating from medical or pharmacokinetics journals not covered by the main search, will be assessed for eligibility and included.

In the following, the detailed searches after journals selections.
Pubmed search:
(("Adv Theory Simul"[Journal]) OR ("Adv Appl Probab"[Journal]) OR ("Adv Data Anal Classif"[Journal]) OR ("Am Stat"[Journal]) OR ("Ann Appl Probab"[Journal]) OR ("Ann Appl Stat"[Journal]) OR ("Ann Math Artif Intell"[Journal]) OR ("Ann Probab"[Journal]) OR ("Ann Stat"[Journal]) OR ("Ann Inst Stat Math"[Journal]) OR ("Annu Rev Stat Appl"[Journal]) OR ("Adv Stat Anal"[Journal]) OR ("Bayesian Anal"[Journal]) OR ("Bernoulli (Andover)"[Journal]) OR ("Biom J"[Journal]) OR ("Biometrics"[Journal]) OR ("Biometrika"[Journal]) OR ("Biostatistics"[Journal]) OR ("Can J Stat"[Journal]) OR ("Chil J Stat"[Journal]) OR ("Commun Stat Appl Methods"[Journal]) OR ("Commun Math Stat"[Journal]) OR ("Commun Stat Simul Comput"[Journal]) OR ("Commun Stat Theory Methods"[Journal]) OR ("Commun Stat Case Stud Data Anal Appl"[Journal]) OR ("Comput Stat"[Journal]) OR ("Comput Stat Data Anal"[Journal]) OR ("Electron J Probab"[Journal]) OR ("Electron J Stat"[Journal]) OR ("ESAIM Probab Stat"[Journal]) OR ("Front Appl Math Stat"[Journal]) OR ("J Math Stat"[Journal]) OR ("Inf inference"[Journal]) OR ("Int J Uncertain Quantif"[Journal]) OR ("Int J Biostat"[Journal]) OR ("Int J Math Comput Sci"[Journal]) OR ("Int J Stat Med Res"[Journal]) OR ("Int Stat Rev"[Journal]) OR ("Jpn J Stat Data Sci"[Journal]) OR ("J Appl Probab"[Journal]) OR ("J Appl Stat"[Journal]) OR ("J Biopharm Stat"[Journal]) OR ("J Mach Learn Res"[Journal]) OR ("J Mod Appl Stat Methods"[Journal]) OR ("J Multivar Anal"[Journal]) OR ("J Nonparametr Stat"[Journal]) OR ("J Off Stat"[Journal]) OR ("J Stat Comput Simul"[Journal]) OR ("J Stat Distrib Appl"[Journal]) OR ("J Stat Plan Inference"[Journal]) OR ("J Stat Softw"[Journal]) OR ("J Stat Theory Appl"[Journal]) OR ("J Stat Theory Pract"[Journal]) OR ("J Surv Stat Methodol"[Journal]) OR ("J Am Stat Assoc"[Journal]) OR ("JIRSS"[Journal]) OR ("J Korean Stat Soc"[Journal]) OR ("J Theor Probab"[Journal]) OR ("Lett Biomath"[Journal]) OR ("Math Biosci"[Journal]) OR ("Metrika"[Journal]) OR ("Metron"[Journal]) OR ("Model Assist Stat Appl"[Journal]) OR ("Monte Carlo Methods Appl"[Journal]) OR ("Pharm Stat"[Journal]) OR ("Revstat Stat J"[Journal]) OR ("Sankhya Ser A"[Journal]) OR ("Sankhya B (2008)"[Journal]) OR ("Sankhya"[Journal]) OR ("Scand Stat Theory Appl"[Journal]) OR ("Seq Anal"[Journal]) OR ("J Comput Math"[Journal]) OR ("Sort (Barc)"[Journal]) OR ("S Afr Stat J"[Journal]) OR ("Stat Neerl"[Journal]) OR ("Stat Sin"[Journal]) OR ("Stat Methods Med Res"[Journal]) OR ("Stat Modelling"[Journal]) OR ("Stat Pap (Berl)"[Journal]) OR ("J Stat Sci Appl"[Journal]) OR ("Stat Theory Relat Fields"[Journal]) OR ("Stat Comput"[Journal]) OR ("Stat Interface"[Journal]) OR ("Stat Probab Lett"[Journal]) OR ("Stat Biopharm Res"[Journal]) OR ("Stat Biosci"[Journal]) OR ("Stat Med"[Journal]) OR ("R J"[Journal]) OR ("Stata J"[Journal]) OR ("Wiley Interdiscip Rev Comput Stat"[Journal]))
AND
((“dose finding”[Title/Abstract]) OR (“dose-finding”[Title/Abstract]) OR (“phase I”[Title/Abstract]) OR (“phase 1”[Title/Abstract]) OR (“phase I/II”[Title/Abstract]) OR (“phase I-II”[Title/Abstract]) OR (“phase 1/2”[Title/Abstract]) OR (“phase 1-2”[Title/Abstract]))
AND

((“maximum tolerated dose”[Title/Abstract]) OR (“cancer”[Title/Abstract]) OR (“oncology”[Title/Abstract]) OR (“toxicity”[Title/Abstract]) OR (“dose escalation”[Title/Abstract]) OR (“dose -escalation”[Title/Abstract]))

(("Clin Trials"[Journal]) OR ("BMC Med Res Methodol"[Journal]) OR ("Contemp Clin Trials"[Journal]) OR ("Contemp Clin Trials Commun"[Journal]) OR ("Comput Methods Programs Biomed"[Journal]) OR ("Control Clin Trials"[Journal]))
AND (("dose finding"[Title/Abstract]) OR ("dose-finding"[Title/Abstract]) OR ("phase I"[Title/Abstract]) OR ("phase 1"[Title/Abstract]) OR ("phase I/II"[Title/Abstract]) OR ("phase I-II"[Title/Abstract]) OR ("phase 1/2"[Title/Abstract]) OR ("phase 1-2"[Title/Abstract]))
AND (("maximum tolerated dose"[Title/Abstract]) OR ("cancer"[Title/Abstract]) OR ("oncology"[Title/Abstract]) OR ("toxicity"[Title/Abstract]) OR ("dose escalation"[Title/Abstract]) OR ("dose-escalation"[Title/Abstract]))

Scopus search:
(TITLE-ABS-KEY ("Phase 1") OR TITLE-ABS-KEY ("Phase I") OR TITLE-ABS-KEY ("Phase I-II") OR TITLE-ABS-KEY ("Phase I/II") OR TITLE-ABS-KEY ("Phase 1-2") OR TITLE-ABS-KEY ("Phase 1/2") OR TITLE-ABS-KEY ("Dose finding") OR TITLE-ABS-KEY ("Dose-finding")) AND (TITLE-ABS-KEY ("Oncology") OR TITLE-ABS-KEY ("Cancer") OR TITLE-ABS-KEY ("Maximum tolerated dose") OR TITLE-ABS-KEY("Toxicity") OR TITLE-ABS-KEY ("Dose escalation") OR TITLE-ABS-KEY ("Dose-escalation")) AND (SRCTITLE('Advanced Mathematical Models and Applications') OR SRCTITLE('Advanced Theory and Simulations') OR SRCTITLE('Advances in Applied Probability') OR SRCTITLE('Advances in Data Analysis and Classification') OR SRCTITLE('Advances in Decision Sciences') OR SRCTITLE('Alea') OR SRCTITLE('American Statistician') OR SRCTITLE('Annales de l'institut Henri Poincare B Probability and Statistics') OR SRCTITLE('Annals of Applied Probability') OR SRCTITLE('Annals of Applied Statistics') OR SRCTITLE('Annals of Data Science') OR SRCTITLE('Annals of Mathematics') OR SRCTITLE('Annals of Probability') OR SRCTITLE('Annals of Statistics') OR SRCTITLE('Annals of the Institute of Statistical Mathematics') OR SRCTITLE('Annual Review of Statistics and Its Application') OR SRCTITLE('AStA Advances in Statistical Analysis') OR SRCTITLE('Australian and New Zealand Journal of Statistics') OR SRCTITLE('Australian Journal of Statistics') OR SRCTITLE('Austrian Journal of Statistics') OR SRCTITLE('Bayesian Analysis') OR SRCTITLE('Bernoulli') OR SRCTITLE('Bioinformatics') OR SRCTITLE('Biometrical Journal') OR SRCTITLE('Biometrics') OR SRCTITLE('Biometrika') OR SRCTITLE('Biostatistics') OR SRCTITLE('Brazilian Journal of Probability and Statistics') OR SRCTITLE('Calcutta Statistical Association Bulletin') OR SRCTITLE('Canadian Journal of Statistics') OR SRCTITLE('Chilean Journal of Statistics') OR SRCTITLE('Communications for Statistical Applications and Methods') OR SRCTITLE('Communications in Mathematics and Statistics') OR SRCTITLE('Communications in Statistics') OR SRCTITLE('Communications in Statistics - Simulation and Computation') OR SRCTITLE('Communications in Statistics - Theory and Methods') OR SRCTITLE('Communications in Statistics Case Studies Data Analysis and Applications') OR SRCTITLE('Communications in Statistics Part B: Simulation and Computation') OR SRCTITLE('Communications in Statistics. Part C: Sequential Analysis') OR SRCTITLE('Communications on Stochastic Analysis') OR SRCTITLE('Computational Statistics') OR SRCTITLE('Computational Statistics & Data Analysis') OR SRCTITLE('Electronic Communications in Probability') OR SRCTITLE('Electronic Journal of Applied Statistical Analysis') OR SRCTITLE('Electronic Journal of Probability') OR SRCTITLE('Electronic Journal of Statistics') OR SRCTITLE('ESAIM - Probability and Statistics') OR SRCTITLE('European Journal of Pure and Applied Mathematics') OR SRCTITLE('Frontiers in Applied Mathematics and Statistics') OR SRCTITLE('Hacettepe Journal of Mathematics and Statistics') OR SRCTITLE('Information and Inference') OR SRCTITLE('International Journal for Uncertainty Quantification') OR SRCTITLE('International Journal of Biostatistics') OR SRCTITLE('International Journal of Mathematics and Computer Science') OR SRCTITLE('International Journal of Statistics in Medical Research') OR SRCTITLE('International Statistical Review') OR SRCTITLE('Japanese Journal of Statistics and Data Science') OR SRCTITLE('Jordan Journal of Mathematics and Statistics') OR SRCTITLE('Journal of Applied Mathematics and Decision Sciences') OR SRCTITLE('Journal of Applied Mathematics and Simulation') OR SRCTITLE('Journal of Applied Probability') OR SRCTITLE('Journal of Applied Probability and Statistics') OR SRCTITLE('Journal of Applied Statistical Science') OR SRCTITLE('Journal of Applied Statistics') OR SRCTITLE('Journal of Biopharmaceutical Statistics') OR

SRCTITLE('Journal of Biostatistics and Epidemiology') OR SRCTITLE('Journal of Machine Learning Research') OR SRCTITLE('Journal of Mathematical Sciences') OR SRCTITLE('Journal of Mathematics and Statistics') OR SRCTITLE('Journal of Mixed Methods Research') OR SRCTITLE('Journal of Modern Applied Statistical Methods') OR SRCTITLE('Journal of Multivariate Analysis') OR SRCTITLE('Journal of Nonparametric Statistics') OR SRCTITLE('Journal of Official Statistics') OR SRCTITLE('Journal of Probability and Statistics') OR SRCTITLE('Journal of Reliability and Statistical Studies') OR SRCTITLE('Journal of Statistical Computation and Simulation') OR SRCTITLE('Journal of Statistical Distributions and Applications') OR SRCTITLE('Journal of Statistical Planning and Inference') OR SRCTITLE('Journal of Statistical Software') OR SRCTITLE('Journal of Statistical Theory and Applications') OR SRCTITLE('Journal of Statistical Theory and Practice') OR SRCTITLE('Journal of Statistics Applications and Probability') OR SRCTITLE('Journal of Survey Statistics and Methodology') OR SRCTITLE('Journal of the American Statistical Association') OR SRCTITLE('Journal of the Iranian Statistical Society') OR SRCTITLE('Journal of the Italian Statistical Society') OR SRCTITLE('Journal of the Korean Statistical Society') OR SRCTITLE('Journal of the Royal Statistical Society Series D: The Statistician') OR SRCTITLE('Journal of the Royal Statistical Society. Series A: Statistics in Society') OR SRCTITLE('Journal of the Royal Statistical Society. Series B: Methodological') OR SRCTITLE('Journal of the Royal Statistical Society. Series B: Statistical Methodology') OR SRCTITLE('Journal of the Royal Statistical Society. Series C: Applied Statistics') OR SRCTITLE('Journal of Theoretical Probability') OR SRCTITLE('Letters in Biomathematics') OR SRCTITLE('Mathematical Biosciences') OR SRCTITLE('Mathematical Methods of Statistics') OR SRCTITLE('Mathematical Sciences') OR SRCTITLE('Mathematical Statistician and Engineering Applications') OR SRCTITLE('Mathematics and Statistics') OR SRCTITLE('Methods, Data, Analyses') OR SRCTITLE('Metrika') OR SRCTITLE('Metron') OR SRCTITLE('Model Assisted Statistics and Applications') OR SRCTITLE('Monte Carlo Methods and Applications') OR SRCTITLE('New Zealand Statistician') OR SRCTITLE('Pakistan Journal of Statistics') OR SRCTITLE('Pakistan Journal of Statistics and Operation Research') OR SRCTITLE('Pharmaceutical Statistics') OR SRCTITLE('Probability and Mathematical Statistics') OR SRCTITLE('Publications of the American Statistical Association') OR SRCTITLE('Quarterly Publications of the American Statistical Association') OR SRCTITLE('R Journal') OR SRCTITLE('Researches in Mathematics') OR SRCTITLE('Revista Brasileira de Biometria') OR SRCTITLE('Revstat Statistical Journal') OR SRCTITLE('Sankhya A') OR SRCTITLE('Sankhya B') OR SRCTITLE('Sankhya: The Indian Journal of Statistics') OR SRCTITLE('Sao Paulo Journal of Mathematical Sciences') OR SRCTITLE('Scandinavian Journal of Statistics') OR SRCTITLE('Sequential Analysis') OR SRCTITLE('SMAI Journal of Computational Mathematics') OR SRCTITLE('SORT') OR SRCTITLE('South African Statistical Journal') OR SRCTITLE('Stat') OR SRCTITLE('Statistica Applicata') OR SRCTITLE('Statistica e Applicazioni') OR SRCTITLE('Statistica Neerlandica') OR SRCTITLE('Statistica Sinica') OR SRCTITLE('Statistical Methodology') OR SRCTITLE('Statistical Methods and Applications') OR SRCTITLE('Statistical Methods in Medical Research') OR SRCTITLE('Statistical Modelling') OR SRCTITLE('Statistical Papers') OR SRCTITLE('Statistical Science') OR SRCTITLE('Statistical Theory and Related Fields') OR SRCTITLE('Statistics') OR SRCTITLE('Statistics and Computing') OR SRCTITLE('Statistics and its Interface') OR SRCTITLE('Statistics and Probability Letters') OR SRCTITLE('Statistics in Biopharmaceutical Research') OR SRCTITLE('Statistics in Biosciences') OR SRCTITLE('Statistics in Medicine') OR SRCTITLE('Statistics, Optimization and Information Computing') OR SRCTITLE('Statistika') OR SRCTITLE('Test') OR SRCTITLE('Thailand Statistician') OR SRCTITLE('The R Journal') OR SRCTITLE('The Stata Journal') OR SRCTITLE('Theory of Probability and its Applications') OR SRCTITLE('Theory of Probability and Mathematical Statistics') OR SRCTITLE('Turkish World Mathematical Society Journal of Applied and Engineering Mathematics') OR SRCTITLE('Wiley Interdisciplinary Reviews: Computational Statistics'))

(TITLE-ABS-KEY ("Phase 1") OR TITLE-ABS-KEY ("Phase I") OR TITLE-ABS-KEY ("Phase I-II") OR TITLE-ABS-KEY ("Phase I/II") OR TITLE-ABS-KEY ("Phase 1-2") OR TITLE-ABS-KEY ("Phase 1/2") OR TITLE-ABS-KEY ("Dose finding") OR TITLE-ABS-KEY ("Dose-finding")) AND (TITLE-ABS-KEY ("Oncology") OR TITLE-ABS-KEY ("Cancer") OR TITLE-ABS-KEY ("Maximum tolerated dose") OR TITLE-ABS-KEY ("Toxicity") OR TITLE-ABS-KEY ("Dose escalation") OR TITLE-ABS-KEY ("Dose-escalation")) AND (SRCTITLE ('bmc AND medical AND research AND methodology') OR SRCTITLE ('contemporary AND clinical AND trials AND communications') OR SRCTITLE ('computer AND methods AND programs AND in AND biomedicine') OR SRCTITLE ('controlled AND clinical AND trials') OR SRCTITLE (

'clinical AND trials') OR SRCTITLE ('contemporary AND clinical AND trials')) AND (LIMIT-TO (EXACTSRCTITLE , "BMC Medical Research Methodology") OR LIMIT-TO (EXACTSRCTITLE , "Clinical Trials") OR LIMIT-TO (EXACTSRCTITLE , "Computer Methods And Programs In Biomedicine") OR LIMIT-TO (EXACTSRCTITLE , "Contemporary Clinical Trials") OR LIMIT-TO (EXACTSRCTITLE , "Contemporary Clinical Trials Communications") OR LIMIT-TO (EXACTSRCTITLE , "Controlled Clinical Trials"))

12. Study records

a. Data management

Data will be exported on Excel sheets. Cleaning and duplicates checking will be performed by AA and MKR.

b. Selection process

First step:
Three of the authors (MKR, EG, MU) will review the journals to retain them or not in the future search for statistical papers on dose-finding methods in oncology. If not all of the three authors agreed on a journal, after discussions a common decision will be taken.

Second step:
Four of the authors (MKR, EG, MU, AA, PM) will independently and randomly review the titles and abstracts of the articles retrieved. Each record will be assessed by at least two reviewers. In cases of disagreement, the reviewers will discuss the article to reach a consensus.

c. Data charting process

All authors (MU, EG, MKR, AA, PM) will pilot test the data extraction form on a few randomly selected papers to assess agreement and feasibility. Subsequently, the full list of articles will be divided among the five reviewers. In cases of uncertainty or disagreement, the paper will be discussed during the authors' meetings (held approximately twice a month) until consensus is reached.

13. Data items

Endpoint	Categories	Comment
Main purpose	Commentary / Review / New design / Benchmark / Comparison / Calibration / Theory / Programming tool / Guidelines	Main purpose of the paper.
Therapy	Monotherapy / Combination / Both	Developed for monotherapy, combination or both. Monotherapy means only one agent is being escalated, compared to combination where the dose levels of both agents are moving.
Toxicity endpoint	Binary / Ordinal / Continuous / Time-to-event / Quasi-binary / No	Type of toxicity endpoint. "No" in the exceptional case where toxicity is not used.

Multivariate toxicity	Yes / No	Incorporate multivariate patient toxicity information such as multiple grades.
Late toxicities	Yes / No	Handling DLTs occurring outside of a short-term evaluation window or accounting for toxicity information at multiple treatment cycles for the dose recommendation.
PRO	Yes / No	Considering patient reported outcome for dose recommendation.
Efficacy endpoint	Binary / Ordinal / Continuous / Time-to-event / Binary and continuous / No	Type of efficacy endpoint when the estimation of the recommended dose accounts for efficacy, otherwise “No”.
Multivariate efficacy	Yes / No	Incorporate multivariate patient efficacy information.
Late efficacies	Yes / No	Handling efficacy occurring outside of a short-term evaluation window.
Activity endpoint	Binary / Ordinal / Continuous / Time-to-event / No	Type of activity endpoint when the estimation of the optimal dose is based on biologic activity endpoint, otherwise “No”.
Multivariate activity	Yes / No	Incorporate multivariate patient activity information.
PK / PD Modelling	Yes / No	Incorporate pharmacokinetic and pharmacodynamic information for the dose recommendation.
Preclinical / Historical	Yes / No	Incorporate preclinical or historical information.
Decision criteria	Safety only / Efficacy-driven / Utility / NA	When only toxicity was used for dose recommendation, “Safety only” was selected. Otherwise, when either efficacy or activity endpoints were used, the recommended dose could be the dose with the highest utility (“Utility”) or highest efficacy under toxicity constraints (“Efficacy-driven”). “NA” was used for Guidelines, Benchmark or Comparison papers.
Randomization	Yes / No	Randomization of patients performed between several dosages.

Comments randomization	During / After escalation	Patients can be randomized either during dose escalation or after a dose-escalation stage to identify acceptable dosages.
Schedules	Yes / No	Can account for different schedules in addition to dose level.
Heterogeneity	Yes / No	Allowing inclusion of covariates in the modeling or determining subgroup-specific recommended dose.
Design Type	Algorithm-based / Model-based / Model-assisted or Curve-free / Other / NA	Class of a design. “NA” applicable for Commentary, Benchmark or Programming tool papers.
Comments design type	Free text	Applicable only for “Other” design types.
Available tool	Yes / No / Upon request / NA	Tools available to perform the design. “NA” applicable for Commentary, Review, Comparison, Theory or Guidelines papers.
Comment tool	Code / R package / App / Upon request / NA	Type of tool available when “Yes” was selected for “Available tool”.

14. Critical appraisal of individual sources of evidence

Not applicable.

15. Synthesis of results

The extracted data will be presented in table format and/or plots and we will provide a narrative summary of the studies by years, type and feature.

Bibliography

FDA. Project Optimus: reforming the dose optimization and dose selection paradigm in oncology. https://www.fda.gov/about-fda/oncology-center-excellence/project-optimus (2021). Accessed: 2023-02-01.

Shah, M., Rahman, A., Theoret, M. R., & Pazdur, R. (2021). The Drug-Dosing Conundrum in Oncology - When Less Is More. The New England journal of medicine, 385(16), 1445–1447. https://doi.org/10.1056/NEJMp2109826
