# Supplementary material for: Evaluating Early‐Stage Oncology Clinical Trial Designs in the Era of Project Optimus: A Scoping Review
Source: Pharm Stat. 2026 Nov 5;25(6):e70131. doi: 10.1002/pst.70131 (PMC13740141; doi:10.1002/pst.70131)
Supplement: Supplementary file 2 [file PST-25-0-s002.pdf]

Supplementary materials S1 of "Evaluating early-stage oncology clinical trial designs in the era of project Optimus: a scoping review"

By M-K Riviere, A Andrillon, P Mozgunov, E Gerard, and M Ursino

1 | METHODOLOGY OF THE SCOPING REVIEW

1.1 | Journals selection

A selection of targeted journals was established which aims to focus on statistical articles dealing with statistical designs for early phase oncology and related papers. For this purpose, from SCOPUS (Elsevier) in "Source Type" the "Journals" item was selected. In the subject area, we chose "Decision Sciences – Statistics, Probability and Uncertainty" and "Mathematics - Statistics and Probability". These two categories are broad and are the most likely to include journals that publish articles on statistical designs. From these categories, we extracted a list of journals. Three authors independently reviewed each journal to determine whether it should be included in the search for statistical papers on dose-finding methods in oncology. Selection was primarily based on the journal's scope; language was also a limiting criterion when publications were not in English. We intentionally covered a wide range of journals to avoid missing relevant articles, while excluding those clearly outside the scope of statistical methods for clinical trials. In addition, well-known clinical journals that publish statistical methodology were added to the list. In cases of disagreement among authors, a meeting was held to discuss and reach consensus.

1.2 | Papers selection

Search strategy and selection criteria

A PubMed search (Medline, Ovid) and a SCOPUS search were performed. All documents published between January 1990 and September 2025 were considered. Keywords used for the search were drawn from two lexical fields, the first related to Phase I studies and the second to oncology: i) "Phase 1" OR "Phase I" OR "phase I-II" OR "phase I/II" OR "phase 1-2" OR "phase 1/2" OR "Dose finding" OR "Dose-finding"; AND ii) "Oncology" OR "Cancer" OR "Maximum tolerated dose" OR "Toxicity" OR "Dose escalation" OR "Dose-escalation". The complete PubMed and Scopus queries are presented in the protocol in Supplementary Material S2.

We extracted a list of articles from both sources (statistical and medical journals from which duplicates and out-of-scope articles were removed. Each article was independently reviewed by at least two authors to decide whether it was eligible for inclusion in the scoping review. In addition, records identified through manual screening of the reference lists of included articles and through the authors' knowledge, originating from medical or pharmacokinetics journals not covered by the main search, were assessed for eligibility and included. We considered eligible: (i) articles published between January 1990 and September 2025; (ii) all designs, including quantitative, qualitative, and mixed-method approaches; (iii) full-text articles published in English; (iv) articles describing statistical methods, rather than applications only, or including a methodological investigation. In cases of disagreement, the selection of the articles was discussed with the remaining authors during scheduled monthly meetings to achieve consensus.

2 | SELECTED JOURNALS

TABLE 1 List of statistical journals extracted from SCOPUS with subject area "Decision Sciences – Statistics, Probability and Uncertainty" and "Mathematics – Statistics and Probability".

Journal Title	Selected
ACM Transactions on Economics and Computation	FALSE
ACRN Journal of Finance and Risk Perspectives	FALSE
Advanced Mathematical Models and Applications	TRUE
Advanced Theory and Simulations	TRUE
Advances in Applied Probability	TRUE
Advances in Data Analysis and Classification	TRUE
Advances in Decision Sciences	TRUE
Advances in Statistical Climatology, Meteorology and Oceanography	FALSE
Alea	TRUE
Allgemeines Statistisches Archiv	FALSE
American Statistician	TRUE
Annales de l'institut Henri Poincare (B) Probability and Statistics	TRUE
Annales de l'Institut Henri Poincare (D) Combinatorics, Physics and their Interactions	FALSE
Annals of Actuarial Science	FALSE
Annals of Applied Probability	TRUE
Annals of Applied Statistics	TRUE
Annals of Data Science	TRUE
Annals of Economics and Statistics	FALSE
Annals of Mathematics	TRUE
Annals of Probability	TRUE
Annals of Statistics	TRUE
Annals of the Institute of Statistical Mathematics	TRUE
Annual Review of Statistics and Its Application	TRUE
Applied Marketing Analytics	FALSE
AStA Advances in Statistical Analysis	TRUE
AStA Wirtschafts- und Sozialstatistisches Archiv	FALSE
Australian and New Zealand Journal of Statistics	TRUE
Australian Journal of Statistics	TRUE
Austrian Journal of Statistics	TRUE
Bayesian Analysis	TRUE
Bernoulli	TRUE
Bioinformatics	TRUE
Biometrical Journal	TRUE
Biometrics	TRUE
Biometrika	TRUE
Biostatistics	TRUE
BioSystems	FALSE
Blätter der DGFVM	FALSE
Boletin de Estadistica e Investigacion Operativa	FALSE
Brazilian Journal of Probability and Statistics	TRUE
British Actuarial Journal	FALSE
British Journal of Mathematical and Statistical Psychology	FALSE
British Journal of Statistical Psychology	FALSE
Bulletin of the Oxford University Institute of Economics & Statistics	FALSE

Journal Title	Selected
Calcutta Statistical Association Bulletin	TRUE
Canadian Journal of Statistics	TRUE
Central European Journal of Economic Modelling and Econometrics	FALSE
Chilean Journal of Statistics	TRUE
Combinatorics Probability and Computing	FALSE
Communications for Statistical Applications and Methods	TRUE
Communications in Analysis and Geometry	FALSE
Communications in Mathematics and Statistics	TRUE
Communications in Statistics	TRUE
Communications in Statistics - Simulation and Computation	TRUE
Communications in Statistics - Theory and Methods	TRUE
Communications in Statistics Case Studies Data Analysis and Applications	TRUE
Communications in Statistics Part B: Simulation and Computation	TRUE
Communications in Statistics. Part C: Sequential Analysis	TRUE
Communications on Stochastic Analysis	TRUE
Comparative Migration Studies	FALSE
Computational Management Science	FALSE
Computational Statistics	TRUE
Computational Statistics & Data Analysis	TRUE
Creativity	FALSE
Croatian Operational Research Review	FALSE
Data-Centric Engineering	FALSE
Decision	FALSE
Dependence Modeling	FALSE
Digital Signal Processing: A Review Journal	FALSE
Dynamic Games and Applications	FALSE
Econometrics and Statistics	FALSE
Economie et Statistique	FALSE
Electronic Communications in Probability	TRUE
Electronic Journal of Applied Statistical Analysis	TRUE
Electronic Journal of Probability	TRUE
Electronic Journal of Statistics	TRUE
Empirical Economics	FALSE
Environmental and Ecological Statistics	FALSE
Environmetrics	FALSE
ESAIM - Probability and Statistics	TRUE
ETS Research Report Series	FALSE
EURO Journal on Decision Processes	FALSE
European Actuarial Journal	FALSE
European Journal of Pure and Applied Mathematics	TRUE
Extremes	FALSE
Finance and Stochastics	FALSE
Foresight and STI Governance	FALSE
Forum of Mathematics, Pi	FALSE
Forum of Mathematics, Sigma	FALSE
Fractal and Fractional	FALSE
Frontiers in Applied Mathematics and Statistics	TRUE
Game Studies	FALSE
Games	FALSE
Global and Stochastic Analysis	FALSE

Journal Title	Selected
Hacettepe Journal of Mathematics and Statistics	TRUE
Infinite Dimensional Analysis, Quantum Probability and Related Topics	FALSE
Information and Inference	TRUE
Information Geometry	FALSE
Insurance: Mathematics and Economics	FALSE
International Game Theory Review	FALSE
International Journal for Uncertainty Quantification	TRUE
International Journal of Agricultural and Statistical Sciences	FALSE
International Journal of Applied Metaheuristic Computing	FALSE
International Journal of Biostatistics	TRUE
International Journal of Business Continuity and Risk Management	FALSE
International Journal of Business Intelligence and Data Mining	FALSE
International Journal of Business Intelligence Research	FALSE
International Journal of Ecological Economics and Statistics	FALSE
International Journal of Game Theory	FALSE
International Journal of Industrial Engineering and Production Research	FALSE
International Journal of Information Science and Management	FALSE
International Journal of Interactive Multimedia and Artificial Intelligence	FALSE
International Journal of Mathematics and Computer Science	TRUE
International Journal of Risk Assessment and Management	FALSE
International Journal of Statistics in Medical Research	TRUE
International Journal of Stochastic Analysis	FALSE
International Journal on Informatics Visualization	FALSE
International Statistical Review	TRUE
Investigacion Operacional	FALSE
Japanese Journal of Statistics and Data Science	TRUE
Jordan Journal of Mathematics and Statistics	TRUE
Journal of Agricultural, Biological, and Environmental Statistics	FALSE
Journal of Applied Analysis	FALSE
Journal of Applied Mathematics and Decision Sciences	TRUE
Journal of Applied Mathematics and Simulation	TRUE
Journal of Applied Mathematics and Stochastic Analysis	FALSE
Journal of Applied Probability	TRUE
Journal of Applied Probability and Statistics	TRUE
Journal of Applied Statistical Science	TRUE
Journal of Applied Statistics	TRUE
Journal of Applied Structural Equation Modeling	FALSE
Journal of Biopharmaceutical Statistics	TRUE
Journal of Biostatistics and Epidemiology	TRUE
Journal of Business	FALSE
Journal of Business and Economic Statistics	FALSE
Journal of Business Cycle Research	FALSE
Journal of Causal Inference	FALSE
Journal of Choice Modelling	FALSE
Journal of Classification	FALSE
Journal of Complexity	FALSE
Journal of Computational and Graphical Statistics	FALSE
Journal of Dynamics and Games	FALSE
Journal of Econometric Methods	FALSE
Journal of Finance and Data Science	FALSE

Journal Title	Selected
Journal of Forecasting	FALSE
Journal of Global Economic Analysis	FALSE
Journal of Grey System	FALSE
Journal of Informetrics	FALSE
Journal of Intelligent and Fuzzy Systems	FALSE
Journal of Machine Learning Research	TRUE
Journal of Management Analytics	FALSE
Journal of Marketing Analytics	FALSE
Journal of Mathematical Imaging and Vision	FALSE
Journal of Mathematical Sciences	TRUE
Journal of Mathematics and Statistics	TRUE
Journal of Mixed Methods Research	TRUE
Journal of Modern Applied Statistical Methods	TRUE
Journal of Multivariate Analysis	TRUE
Journal of Nonparametric Statistics	TRUE
Journal of Official Statistics	TRUE
Journal of Physics A: Mathematical and Theoretical	FALSE
Journal of Privacy and Confidentiality	FALSE
Journal of Probability and Statistics	TRUE
Journal of Reliability and Statistical Studies	TRUE
Journal of Risk Management in Financial Institutions	FALSE
Journal of Safety Science and Resilience	FALSE
Journal of Soviet Mathematics	FALSE
Journal of Statistical Computation and Simulation	TRUE
Journal of Statistical Distributions and Applications	TRUE
Journal of Statistical Mechanics: Theory and Experiment	FALSE
Journal of Statistical Planning and Inference	TRUE
Journal of Statistical Software	TRUE
Journal of Statistical Theory and Applications	TRUE
Journal of Statistical Theory and Practice	TRUE
Journal of Statistics and Data Science Education	FALSE
Journal of Statistics Applications and Probability	TRUE
Journal of Statistics Education	FALSE
Journal of Survey Statistics and Methodology	TRUE
Journal of Systems Science and Information	FALSE
Journal of Targeting, Measurement and Analysis for Marketing	FALSE
Journal of the American Statistical Association	TRUE
Journal of the Iranian Statistical Society	TRUE
Journal of the Italian Statistical Society	TRUE
Journal of the Korean Statistical Society	TRUE
Journal of the Operational Research Society	FALSE
Journal of the Royal Statistical Society Series D: The Statistician	TRUE
Journal of the Royal Statistical Society. Series A: Statistics in Society	TRUE
Journal of the Royal Statistical Society. Series B: Methodological	TRUE
Journal of the Royal Statistical Society. Series B: Statistical Methodology	TRUE
Journal of the Royal Statistical Society. Series C: Applied Statistics	TRUE
Journal of the Statistical and Social Inquiry Society of Ireland	FALSE
Journal of Theoretical Biology	FALSE
Journal of Theoretical Probability	TRUE
Journal of Time Series Analysis	FALSE

Journal Title	Selected
Journal of Transportation and Statistics	FALSE
Journal of Verification, Validation and Uncertainty Quantification	FALSE
Law, Probability and Risk	FALSE
Lecturas de Economia	FALSE
Letters in Biomathematics	TRUE
Markov Processes and Related Fields	FALSE
Mathematical Biosciences	TRUE
Mathematical Methods of Statistics	TRUE
Mathematical Physics Electronic Journal	FALSE
Mathematical Sciences	TRUE
Mathematical Social Sciences	FALSE
Mathematical Statistician and Engineering Applications	TRUE
Mathematics and Financial Economics	FALSE
Mathematics and Statistics	TRUE
Measurement	FALSE
Measurement: Journal of the International Measurement Confederation	FALSE
Methodology and Computing in Applied Probability	FALSE
Methods, Data, Analyses	TRUE
Metodoloski Zvezki	FALSE
Metrika	TRUE
Metron	TRUE
Model Assisted Statistics and Applications	TRUE
Modeling Earth Systems and Environment	FALSE
Modern Stochastics: Theory and Applications	FALSE
Monte Carlo Methods and Applications	TRUE
Multivariate Behavioral Research	FALSE
Networks and Heterogeneous Media	FALSE
New Zealand Statistician	TRUE
Nonautonomous Dynamical Systems	FALSE
North American Actuarial Journal	FALSE
Observational Studies	FALSE
OECD Journal: Journal of Business Cycle Measurement and Analysis	FALSE
Open Systems and Information Dynamics	FALSE
Operational Research	FALSE
Operations and Supply Chain Management	FALSE
Operations Research and Decisions	FALSE
Operations Research Perspectives	FALSE
Oxford Bulletin of Economics and Statistics	FALSE
Pakistan Journal of Statistics	TRUE
Pakistan Journal of Statistics and Operation Research	TRUE
Pharmaceutical Statistics	TRUE
Physica A: Statistical Mechanics and its Applications	FALSE
Physical Review E	FALSE
Probability and Mathematical Statistics	TRUE
Probability in the Engineering and Informational Sciences	FALSE
Probability Surveys	FALSE
Probability Theory and Related Fields	FALSE
Probability, Uncertainty and Quantitative Risk	FALSE
Psicologica	FALSE
Publications of the American Statistical Association	TRUE

Journal Title	Selected
Quality and Quantity	FALSE
Quarterly Publications of the American Statistical Association	TRUE
Queueing Systems	FALSE
R Journal	TRUE
Random Matrices: Theory and Application	FALSE
Random Operators and Stochastic Equations	FALSE
Regional Statistics	FALSE
Reliability: Theory and Applications	FALSE
Researches in Mathematics	TRUE
Revista Brasileira de Biometria	TRUE
Revista Colombiana de Estadistica	FALSE
Revstat Statistical Journal	TRUE
Risk and Decision Analysis	FALSE
Risk, Decision and Policy	FALSE
Sankhya A	TRUE
Sankhya B	TRUE
Sankhya: The Indian Journal of Statistics	TRUE
Sao Paulo Journal of Mathematical Sciences	TRUE
Scandinavian Actuarial Journal	FALSE
Scandinavian Journal of Statistics	TRUE
Scientific data	FALSE
Sequential Analysis	TRUE
Set-Valued and Variational Analysis	FALSE
SIAM-ASA Journal on Uncertainty Quantification	FALSE
Significance	FALSE
SMAI Journal of Computational Mathematics	TRUE
Socio-Economic Planning Sciences	FALSE
SORT	TRUE
South African Statistical Journal	TRUE
Spatial Economic Analysis	FALSE
Spatial Statistics	FALSE
Stat	TRUE
Statistica Applicata	TRUE
Statistica e Applicazioni	TRUE
Statistica Neerlandica	TRUE
Statistica Sinica	TRUE
Statistical Applications in Genetics and Molecular Biology	FALSE
Statistical Inference for Stochastic Processes	FALSE
Statistical Journal of the IAOS	FALSE
Statistical Methodology	TRUE
Statistical Methods and Applications	TRUE
Statistical Methods in Medical Research	TRUE
Statistical Modelling	TRUE
Statistical Papers	TRUE
Statistical Science	TRUE
Statistical Theory and Related Fields	TRUE
Statistics	TRUE
Statistics and Computing	TRUE
Statistics and its Interface	TRUE
Statistics and Probability Letters	TRUE

Journal Title	Selected
Statistics and Public Policy	FALSE
Statistics and Risk Modeling	FALSE
Statistics Education Research Journal	FALSE
Statistics in Biopharmaceutical Research	TRUE
Statistics in Biosciences	TRUE
Statistics in Medicine	TRUE
Statistics in Transition	FALSE
Statistics Surveys	FALSE
Statistics, Optimization and Information Computing	TRUE
Statistics, Politics and Policy	FALSE
Statistika	TRUE
Statistische Hefte	FALSE
Statisztikai Szemle	FALSE
Stochastic Analysis and Applications	FALSE
Stochastic Models	FALSE
Stochastic Processes and their Applications	FALSE
Stochastic Systems	FALSE
Stochastics	FALSE
Stochastics and Partial Differential Equations: Analysis and Computations	FALSE
Stochastics and Quality Control	FALSE
Survey Methodology	FALSE
Swiss Journal of Economics and Statistics	FALSE
Teaching Statistics	FALSE
Technometrics	FALSE
Teruleti Statisztika	FALSE
Test	TRUE
Thailand Statistician	TRUE
The R Journal	TRUE
The Stata Journal	TRUE
Theory in Biosciences	FALSE
Theory of Probability and its Applications	TRUE
Theory of Probability and Mathematical Statistics	TRUE
Theory of Stochastic Processes	FALSE
Trabajos de Estadistica	FALSE
Trabajos de Estadistica Y de Investigacion Operativa	FALSE
Trabajos de Investigacion Operativa	FALSE
Transactions on Data Privacy	FALSE
Transplant Research and Risk Management	FALSE
Turkish World Mathematical Society Journal of Applied and Engineering Mathematics	TRUE
Uncertain Supply Chain Management	FALSE
Utilitas Mathematica	FALSE
Vital and health statistics. Ser. 1: Programs and collection procedures	FALSE
Vital and health statistics. Series 10, Data from the National Health Survey	FALSE
Vital and health statistics. Series 13, Data from the National Health Survey	FALSE
Vital and health statistics. Series 2, Data evaluation and methods research	FALSE
Wiley Interdisciplinary Reviews: Computational Statistics	TRUE
WSEAS Transactions on Mathematics	FALSE
Zeitschrift für Wahrscheinlichkeitstheorie und Verwandte Gebiete	FALSE
Zeitschrift für Evaluation	FALSE
Zhurnal Belorusskogo Gosudarstvennogo Universiteta. Matematika. Informatika	FALSE

TABLE 2 List of additional medical journals publishing statistical methods added to the list of statistical journals.

**Journal Title**
BMC Medical Research Methodology
Clinical Trials
Computer Methods and Programs in Biomedicine
Contemporary Clinical Trials
Contemporary Clinical Trials Communications
Controlled Clinical Trials

3 | FULL LIST OF ARTICLES

The exhaustive list of articles retained is the following:

1. Iasonos A, Wheeler GM. "Improving the performance of Bayesian logistic regression model with overdose control in oncology dose-finding studies" by Hongtao Zhang, Alan Chiang, and Jixian Wang. *Statistics in Medicine* 2022; 41(27): 5494–5496.
2. Gao F, Trinkaus K, Miller JP. 17 Early Phase Clinical Trials: Phases I and II. *Handbook of statistics* 2007; 27: 513–545.
3. Yada S. A Bayesian adaptive design for addressing correlated late-onset outcomes in phase I/II randomized trials of drug combinations in oncology. *Communications in Statistics-Simulation and Computation* 2022; 51(6): 2963–2977.
4. Tighiouart M, Cook-Wiens G, Rogatko A. A Bayesian adaptive design for cancer phase I trials using a flexible range of doses. *Journal of biopharmaceutical statistics* 2018; 28(3): 562–574.
5. Tighiouart M, Li Q, Rogatko A. A Bayesian adaptive design for estimating the maximum tolerated dose curve using drug combinations in cancer phase I clinical trials. *Statistics in medicine* 2017; 36(2): 280–290.
6. Diniz MA, Kim S, Tighiouart M. A Bayesian adaptive design in cancer phase I trials using dose combinations in the presence of a baseline covariate. *Journal of probability and statistics* 2018; 2018(1): 8654173.
7. Kim SB, Gillen DL. A Bayesian adaptive dose-finding algorithm for balancing individual-and population-level ethics in phase I clinical trials. *Sequential Analysis* 2016; 35(4): 423–439.
8. Zhang Y, Cao S, Zhang C, Jin IH, Zang Y. A Bayesian adaptive phase I/II clinical trial design with late-onset competing risk outcomes. *Biometrics* 2021; 77(3): 796–808.
9. Cunanan KM, Koopmeiners JS. A Bayesian adaptive phase I–II trial design for optimizing the schedule of therapeutic cancer vaccines. *Statistics in medicine* 2017; 36(1): 43–53.
10. Whitehead J, Zhou Y, Hampson L, Ledent E, Pereira A. A Bayesian approach for dose-escalation in a phase I clinical trial incorporating pharmacodynamic endpoints. *Journal of Biopharmaceutical statistics* 2007; 17(6): 1117–1129.
11. Sinclair K, Whitehead A. A Bayesian approach to dose-finding studies for cancer therapies: incorporating later cycles of therapy. *Statistics in medicine* 2014; 33(15): 2665–2680.
12. Bekele BN, Shen Y. A Bayesian approach to jointly modeling toxicity and biomarker expression in a phase I/II dose-finding trial. *Biometrics* 2005; 61(2): 343–354.
13. Bailey S, Neuenschwander B, Laird G, Branson M. A Bayesian case study in oncology phase I combination dose-finding using logistic regression with covariates. *Journal of biopharmaceutical statistics* 2009; 19(3): 469–484.
14. Zheng H, Hampson LV. A Bayesian decision-theoretic approach to incorporate preclinical information into phase I oncology trials. *Biometrical Journal* 2020; 62(6): 1408–1427.

15. Wang C, Rosner GL, Roden RB. A Bayesian design for phase I cancer therapeutic vaccine trials. *Statistics in medicine* 2019; 38(7): 1170–1189.

16. Riviere MK, Yuan Y, Dubois F, Zohar S. A Bayesian dose finding design for clinical trials combining a cytotoxic agent with a molecularly targeted agent. *Journal of the Royal Statistical Society Series C: Applied Statistics* 2015; 64(1): 215–229.

17. Loke YC, Tan SB, Cai Y, Machin D. A Bayesian dose finding design for dual endpoint phase I trials. *Statistics in medicine* 2006; 25(1): 3–22.

18. Cai C, Yuan Y, Ji Y. A Bayesian dose finding design for oncology clinical trials of combinational biological agents. *Journal of the Royal Statistical Society Series C: Applied Statistics* 2014; 63(1): 159–173.

19. Riviere MK, Yuan Y, Dubois F, Zohar S. A Bayesian dose-finding design for drug combination clinical trials based on the logistic model. *Pharmaceutical statistics* 2014; 13(4): 247–257.

20. Liu S, Ning J. A Bayesian dose-finding design for drug combination trials with delayed toxicities. *Bayesian analysis* 2013; 8(3): 703.

21. Guo B, Yuan Y. A Bayesian dose-finding design for phase I/II clinical trials with nonignorable dropouts. *Statistics in Medicine* 2015; 34(10): 1721–1732.

22. Lyu J, Curran E, Ji Y. Bayesian adaptive design for finding the maximum tolerated sequence of doses in multicycle dose-finding clinical trials. *JCO Precision Oncology* 2018; 2: 1–19.

23. Whitehead J, Thygesen H, Whitehead A. A Bayesian dose-finding procedure for phase I clinical trials based only on the assumption of monotonicity. *Statistics in medicine* 2010; 29(17): 1808–1824.

24. Yada S, Hamada C. A Bayesian hierarchal modeling approach to shortening phase I/II trials of anticancer drug combinations. *Pharmaceutical Statistics* 2018; 17(6): 750–760.

25. Wheeler GM, Sweeting MJ, Mander AP. A Bayesian model-free approach to combination therapy phase I trials using censored time-to-toxicity data. *Journal of the Royal Statistical Society Series C: Applied Statistics* 2019; 68(2): 309–329.

26. Muliere P, Walker S. A Bayesian nonparametric approach to determining a maximum tolerated dose. *Journal of Statistical Planning and Inference* 1997; 61(2): 339–353.

27. Guo B, Zang Y. A Bayesian phase I/II biomarker-based design for identifying subgroup-specific optimal dose for immunotherapy. *Statistical methods in medical research* 2022; 31(6): 1104–1119.

28. Guo B, Zang Y, Yuan Y. A Bayesian phase I/II clinical trial design in the presence of informative dropouts. *Statistics and Its Interface* 2015; 8(2): 217–226.

29. Guo B, Garrett-Mayer E, Liu S. A Bayesian phase I/II design for cancer clinical trials combining an immunotherapeutic agent with a chemotherapeutic agent. *Journal of the Royal Statistical Society Series C: Applied Statistics* 2021; 70(5): 1210–1229.

30. Guo B, Zang Y, Lin LH, Zhang R. A Bayesian phase I/II design to determine subgroup-specific optimal dose for immunotherapy sequentially combined with radiotherapy. *Pharmaceutical statistics* 2023; 22(1): 143–161.

31. Liu S, Guo B, Yuan Y. A Bayesian phase I/II trial design for immunotherapy. *Journal of the American Statistical Association* 2018; 113(523): 1016–1027.

32. Jiménez JL, Kim S, Tighiouart M. A Bayesian seamless phase I–II trial design with two stages for cancer clinical trials with drug combinations. *Biometrical Journal* 2020; 62(5): 1300–1314.

33. Zheng W, Zhao Y, Lu Y, Miao H, Liu H. A Bayesian three-parameter logistic model for early-and late-onset DLTs in oncology phase I studies. *Journal of biopharmaceutical statistics* 2016; 26(2): 339–351.

34. Pantoja K, Lanke S, Munafo A, et al. Designing phase I oncology dose escalation using dose–exposure–toxicity models as a complementary approach to model-based dose–toxicity models. *CPT: Pharmacometrics & Systems Pharmacology* 2022; 11(10): 1371–1381.

35. Wanitjirattikal P, Shi C. A Bayesian zero-inflated binomial regression and its application in dose-finding study. *Journal of Biopharmaceutical Statistics* 2020; 30(2): 322–333.

36. Mozgunov P, Jaki T, Paoletti X. A benchmark for dose finding studies with continuous outcomes. *Biostatistics* 2020; 21(2): 189–201.

37. Mozgunov P, Paoletti X, Jaki T. A benchmark for dose-finding studies with unknown ordering. *Biostatistics* 2022; 23(3): 721–737.

38. Su Z. A class of designs for phase I cancer clinical trials combining Bayesian and likelihood approaches. *Journal of Applied Statistics* 2011; 38(7): 1493–1498.

39. Hirakawa A, Wages NA, Sato H, Matsui S. A comparative study of adaptive dose-finding designs for phase I oncology trials of combination therapies. *Statistics in medicine* 2015; 34(24): 3194–3213.

40. Zhou Y, Li R, Yan F, Lee JJ, Yuan Y. A comparative study of Bayesian optimal interval (BOIN) design with interval 3+3 (i3+3) design for phase I oncology dose-finding trials. *Statistics in biopharmaceutical research* 2021; 13(2): 147–155.

41. Paoletti X, Kramar A. A comparison of model choices for the continual reassessment method in phase I cancer trials. *Statistics in medicine* 2009; 28(24): 3012–3028.

42. Korn EL, Midthune D, Chen TT, Rubinstein LV, Christian MC, Simon RM. A comparison of two phase I trial designs. *Statistics in medicine* 1994; 13(18): 1799–1806.

43. Zhang W, Wang X, Muthukumarana S, Yang P. A continual reassessment method without undue risk of toxicity. *Communications in Statistics-Simulation and Computation* 2023; 52(4): 1179–1191.

44. Fan S, Lee BL, Lu Y. A curve-free Bayesian decision-theoretic design for phase Ia/Ib trials considering both safety and efficacy outcomes. *Statistics in biosciences* 2020; 12: 146–166.

45. Lee BL, Fan SK, Lu Y. A curve-free Bayesian decision-theoretic design for two-agent phase I trials. *Journal of biopharmaceutical statistics* 2017; 27(1): 34–43.

46. Gasparini M, Eisele J. A curve-free method for phase I clinical trials. *Biometrics* 2000; 56(2): 609–615.

47. Lee J, Thall PF, Ji Y, Müller P. A decision-theoretic phase I–II design for ordinal outcomes in two cycles. *Biostatistics* 2016; 17(2): 304–319.

48. Pan H, Yuan Y. A default method to specify skeletons for Bayesian model averaging continual reassessment method for phase I clinical trials. *Statistics in medicine* 2017; 36(2): 266–279.

49. Conaway MR. A design for phase I trials in completely or partially ordered groups. *Statistics in medicine* 2017; 36(15): 2323–2332.

50. Ursino M, Yuan Y, Alberti C, et al. A dose finding design for seizure reduction in neonates. *Journal of the Royal Statistical Society Series C: Applied Statistics* 2019; 68(2): 427–444.

51. Hirakawa A, Hamada C, Matsui S. A dose-finding approach based on shrunken predictive probability for combinations of two agents in phase I trials. *Statistics in medicine* 2013; 32(26): 4515–4525.

52. Kaneko S, Hirakawa A, Kakurai Y, Hamada C. A dose-finding approach for genomic patterns in phase I trials. *Journal of Biopharmaceutical Statistics* 2020; 30(5): 834–853.

53. Mozgunov P, Cro S, Lingford-Hughes A, Paterson LM, Jaki T. A dose-finding design for dual-agent trials with patient-specific doses for one agent with application to an opiate detoxification trial. *Pharmaceutical statistics* 2022; 21(2): 476–495.

54. Kakurai Y, Hirakawa A, Hamada C. A dose-finding method based on multiple dosing in two-agent combination phase I trials. *Journal of Biopharmaceutical Statistics* 2015; 25(5): 1065–1076.

55. Yan F, Mandrekar SJ, Yuan Y. Keyboard: a novel Bayesian toxicity probability interval design for phase I clinical trials. *Clinical Cancer Research* 2017; 23(15): 3994–4003.

56. Mozgunov P, Jaki T. A flexible design for advanced Phase I/II clinical trials with continuous efficacy endpoints. *Biometrical Journal* 2019; 61(6): 1477–1492.

57. Braun TM, Jia N. A generalized continual reassessment method for two-agent phase I trials. *Statistics in biopharmaceutical research* 2013; 5(2): 105–115.

58. Braun TM, Wang S. A hierarchical Bayesian design for phase I trials of novel combinations of cancer therapeutic agents. *Biometrics* 2010; 66(3): 805–812.

59. Chapple AG, Thall PF. A hybrid phase I-II/III clinical trial design allowing dose re-optimization in phase III. *Biometrics* 2019; 75(2): 371–381.

60. Yin G, Yuan Y. A latent contingency table approach to dose finding for combinations of two agents. *Biometrics* 2009; 65(3): 866–875.

61. Wason JM, Seaman SR. A latent variable model for improving inference in trials assessing the effect of dose on toxicity and composite efficacy endpoints. *Statistical methods in medical research* 2020; 29(1): 230–242.

62. Murphy JR, Hall DL. A logistic dose-ranging method for phase I clinical investigations trials. *Journal of Biopharmaceutical Statistics* 1997; 7(4): 635–647.

63. Yin J, Qin R, Ezzalfani M, Sargent DJ, Mandrekar SJ. A Bayesian dose-finding design incorporating toxicity data from multiple treatment cycles. *Statistics in medicine* 2017; 36(1): 67–80.

64. Bartroff J, Lai TL, Narasimhan B. A new approach to designing phase I-II cancer trials for cytotoxic chemotherapies. *Statistics in medicine* 2014; 33(16): 2718–2735.

65. Mu R, Xu J. A new Bayesian dose-finding design for drug combination trials. *Statistics in Biopharmaceutical Research* 2017; 9(4): 384–389.

66. Guo W, Wang SJ, Yang S, Lynn H, Ji Y. A Bayesian interval dose-finding design addressingOckham's razor: mTPI-2. *Contemporary clinical trials* 2017; 58: 23–33.

67. Ivanova A. A new dose-finding design for bivariate outcomes. *Biometrics* 2003; 59(4): 1001–1007.

68. Tremmel L. A new look at evaluating MTD designs in cancer research. 2009; 3: 371–379.

69. Ivanova A, Wang K. A non-parametric approach to the design and analysis of two-dimensional dose-finding trials. *Statistics in Medicine* 2004; 23(12): 1861–1870.

70. Razaee ZS, Cook-Wiens G, Tighiouart M. A nonparametric Bayesian method for dose finding in drug combinations cancer trials. *Statistics in medicine* 2022; 41(6): 1059–1080.

71. Tian T. A note on continual reassessment method. *Statistics & Probability Letters* 2016; 113: 94–102.

72. Azriel D. A note on the robustness of the continual reassessment method. *Statistics & Probability Letters* 2012; 82(5): 902–906.

73. Whitehead J, Thygesen H, Jaki T, et al. A novel Phase I/IIa design for early phase oncology studies and its application in the evaluation of MK-0752 in pancreatic cancer. *Statistics in medicine* 2012; 31(18): 1931–1943.

74. Huang X, Biswas S, Oki Y, Issa JP, Berry DA. A parallel Phase I/II clinical trial design for combination therapies. *Biometrics* 2007; 63(2): 429–436.

75. Günhan BK, Weber S, Friede T. A Bayesian time-to-event pharmacokinetic model for phase I dose-escalation trials with multiple schedules. *Statistics in Medicine* 2020; 39(27): 3986–4000.

76. Yang X, Ye K. A phase I dose-finding study based on polychotomous toxicity responses. *Statistics and Its Interface* 2012; 5(4): 451–461.

77. Wages NA, Read PW, Petroni GR. A phase I/II adaptive design for heterogeneous groups with application to a stereotactic body radiation therapy trial. *Pharmaceutical statistics* 2015; 14(4): 302–310.

78. Braun TM, Kang S, Taylor JM. A phase I/II trial design when response is unobserved in subjects with dose-limiting toxicity. *Statistical methods in medical research* 2016; 25(2): 659–673.

79. Lin R, Thall PF, Yuan Y. A phase I-II basket trial design to optimize dose-schedule regimes based on delayed outcomes. *Bayesian analysis* 2021; 16(1): 179.

80. Lee J, F. Thall P, Msaouel P. A phase I-II design based on periodic and continuous monitoring of disease status and the times to toxicity and death. *Statistics in medicine* 2020; 39(15): 2035–2050.

81. Zhang L, Yuan Y. A practical Bayesian design to identify the maximum tolerated dose contour for drug combination trials. *Statistics in medicine* 2016; 35(27): 4924–4936.

82. Asakawa T, Hamada C. A pragmatic dose-finding approach using short-term surrogate efficacy outcomes to evaluate binary efficacy and toxicity outcomes in phase I cancer clinical trials. *Pharmaceutical Statistics* 2013; 12(5): 315–327.

83. Mander AP, Sweeting MJ. A product of independent beta probabilities dose escalation design for dual-agent phase I trials. *Statistics in medicine* 2015; 34(8): 1261–1276.

84. Durham SD, Flournoy N, Rosenberger WF. A random walk rule for phase I clinical trials. *Biometrics* 1997: 745–760.

85. Dejardin D, Lesaffre E, Hamberg P, Verweij J. A randomized phase I Bayesian dose escalation design for the combination of anti-cancer drugs. *Pharmaceutical Statistics* 2014; 13(3): 196–207.

86. Liu S, Johnson VE. A robust Bayesian dose-finding design for phase I/II clinical trials. *Biostatistics* 2016; 17(2): 249–263.

87. Zheng H, Hampson LV, Wandel S. A robust Bayesian meta-analytic approach to incorporate animal data into phase I oncology trials. *Statistical methods in medical research* 2020; 29(1): 94–110.

88. Zang Y, Lee JJ. A robust two-stage design identifying the optimal biological dose for phase I/II clinical trials. *Statistics in medicine* 2017; 36(1): 27–42.

89. Hüsing J, Sauerwein W, Hideghéty K, Jöckel KH. A scheme for a dose-escalation study when the event is lagged. *Statistics in medicine* 2001; 20(22): 3323–3334.

90. Su X, Li Y, Müller P, Hsu CW, Pan H, Do KA. A semi-mechanistic dose-finding design in oncology using pharmacokinetic/pharmacodynamic modeling. *Pharmaceutical statistics* 2022; 21(6): 1149–1166.

91. Fan SK, Lu Y, Wang YG. A simple Bayesian decision-theoretic design for dose-finding trials. *Statistics in Medicine* 2012; 31(28): 3719–3730.

92. Cheung YK, Chappell R. A simple technique to evaluate model sensitivity in the continual reassessment method. *Biometrics* 2002; 58(3): 671–674.

93. Biard L, Cheng B, Manji GA, Lee SM. A simulation study of approaches for handling disease progression in dose-finding clinical trials. *Journal of biopharmaceutical statistics* 2021; 31(2): 156–167.

94. Morita S, Thall PF, Takeda K. A simulation study of methods for selecting subgroup-specific doses in phase 1 trials. *Pharmaceutical statistics* 2017; 16(2): 143–156.

95. Guo B, Li Y, Yuan Y. A dose–schedule finding design for phase I–II clinical trials. *Journal of the Royal Statistical Society Series C: Applied Statistics* 2016; 65(2): 259–272.

96. O'QUIGLEY J, Reiner E. A stopping rule for the continual reassessment method. *Biometrika* 1998; 85(3): 741–748.

97. Thall PF, Russell KE. A strategy for dose-finding and safety monitoring based on efficacy and adverse outcomes in phase I/II clinical trials. *Biometrics* 1998: 251–264.

98. Yang X, Ye K, Wang Y. A study of the probit model with latent variables in phase I clinical trials. *Journal of Statistical Computation and Simulation* 2015; 85(8): 1621–1633.

99. Mozgunov P, Gasparini M, Jaki T. A surface-free design for phase I dual-agent combination trials. *Statistical methods in medical research* 2020; 29(10): 3093–3109.

100. Ye K, Yang X, Ji Y, Wang M. A system for determining maximum tolerated dose in clinical trial. *Statistical Theory and Related Fields* 2021; 5(4): 288–302.

101. Pan H, Li C, Wang L, Jiang Z, Xia J. A Three-stage Bayesian Adaptive Phase I/II Design and Simulation Studies. *Communications in Statistics-Simulation and Computation* 2014; 43(2): 254–268.

102. Zhong W, Koopmeiners JS, Carlin BP. A trivariate continual reassessment method for phase I/II trials of toxicity, efficacy, and surrogate efficacy. *Statistics in medicine* 2012; 31(29): 3885–3895.

103. Lee BL, Fan SK. A two-dimensional search algorithm for dose-finding trials of two agents. *Journal of biopharmaceutical statistics* 2012; 22(4): 802–818.

104. Wang O, Faries DE. A two-stage dose selection strategy in phase I trials with wide dose ranges. *Journal of Biopharmaceutical Statistics* 2000; 10(3): 319–333.

105. Zhou Y, Lee JJ, Yuan Y. A utility-based Bayesian optimal interval (U-BOIN) phase I/II design to identify the optimal biological dose for targeted and immune therapies. *Statistics in Medicine* 2019; 38(28): S5299–S5316.

106. Guo B, Park Y, Liu S. A utility-based Bayesian phase I–II design for immunotherapy trials with progression-free survival end point. *Journal of the Royal Statistical Society Series C: Applied Statistics* 2019; 68(2): 411–425.

107. Thall PF, Jack Lee J, Tseng CH, Estey EH. Accrual strategies for phase I trials with delayed patient outcome. *Statistics in Medicine* 1999; 18(10): 1155–1169.

108. Liu R, Yuan Y, Sen S, et al. Accuracy and safety of novel designs for phase I drug-combination oncology trials. *Statistics in biopharmaceutical research* 2022; 14(3): 270–282.

109. McGree JM, Drovandi CC, Thompson M, et al. Adaptive Bayesian compound designs for dose finding studies. *Journal of Statistical Planning and Inference* 2012; 142(6): 1480–1492.

110. Wang M, Day R. Adaptive Bayesian design for phase I dose-finding trials using a joint model of response and toxicity. *Journal of biopharmaceutical statistics* 2009; 20(1): 125–144.

111. Zhang Y, Kutner M, Chen Z. Adaptive Bayesian phase I clinical trial designs for estimating the maximum tolerated doses for two drugs while fully utilizing all toxicity information. *Biometrical Journal* 2021; 63(7): 1476–1492.

112. Sverdlov O, Wong WK, Ryeznik Y. Adaptive clinical trial designs for phase I cancer studies. *Statistics Surveys* 2014; 8: 2–44.

113. Thall PF, Inoue LY, Martin TG. Adaptive decision making in a lymphocyte infusion trial. *Biometrics* 2002; 58(3): 560–568.

114. Heyd JM, Carlin BP. Adaptive design improvements in the continual reassessment method for phase I studies. *Statistics in medicine* 1999; 18(11): 1307–1321.

115. Dragalin V, Fedorov V. Adaptive designs for dose-finding based on efficacy–toxicity response. *Journal of Statistical Planning and Inference* 2006; 136(6): 1800–1823.

116. Dragalin V, Fedorov V, Wu Y. Adaptive designs for selecting drug combinations based on efficacy–toxicity response. *Journal of Statistical Planning and Inference* 2008; 138(2): 352–373.

117. Potter DM. Adaptive dose finding for phase I clinical trials of drugs used for chemotherapy of cancer. *Statistics in Medicine* 2002; 21(13): 1805–1823.

118. Chu Y, Pan H, Yuan Y. Adaptive dose modification for phase I clinical trials. *Statistics in medicine* 2016; 35(20): 3497–3508.

119. Thall PF, Cook JD, Estey EH. Adaptive dose selection using efficacy-toxicity trade-offs: illustrations and practical considerations. *Journal of Biopharmaceutical Statistics* 2006; 16(5): 623–638.

120. Yang Y, Fang HB, Roy A, Tan M. Adaptive oncology phase I trial design of drug combinations with drug-drug interaction modeling. *Statistics and Its Interface* 2018; 11(1): 109–127.

121. He W, Liu J, Binkowitz B, Quan H. A model-based approach in the estimation of the maximum tolerated dose in phase I cancer clinical trials. *Statistics in medicine* 2006; 25(12): 2027–2042.

122. Yada S, Hamada C. Adaptive phase I/II clinical trials for drug combination assessment in oncology using the outcomes of each cycle. *Pharmaceutical Statistics* 2017; 16(6): 433–444.

123. Zhang J, Braun TM, Taylor JM. Adaptive prior variance calibration in the Bayesian continual reassessment method. *Statistics in medicine* 2013; 32(13): 2221–2234.

124. Thall PF, Nguyen HQ. Adaptive randomization to improve utility-based dose-finding with bivariate ordinal outcomes. *Journal of biopharmaceutical statistics* 2012; 22(4): 785–801.

125. Zhou Y, Zhao Y, Cicconetti G, et al. AIDE: Adaptive intrapatient dose escalation designs to accelerate Phase I clinical trials. *Pharmaceutical statistics* 2023; 22(2): 300–311.

126. He W, Liu J. Algorithm-Based Designs. *Methods and Applications of Statistics in Clinical Trials: Concepts, Principles, Trials, and Design* 2014: 40–52.

127. Jiang Z, Mi G, Lin J, Lorenzato C, Ji Y. A multi-arm two-stage (MATS) design for proof-of-concept and dose optimization in early-phase oncology trials. *Contemporary Clinical Trials* 2023; 132: 107278.

128. Hirakawa A. An adaptive dose-finding approach for correlated bivariate binary and continuous outcomes in phase I oncology trials. *Statistics in medicine* 2012; 31(6): 516–532.

129. Chiuzan C, Garrett-Mayer E, Nishimura MI. An adaptive dose-finding design based on both safety and immunologic responses in cancer clinical trials. *Statistics in biopharmaceutical research* 2018; 10(3): 185–195.

130. Zhang W, Sargent DJ, Mandrekar S. An adaptive dose-finding design incorporating both toxicity and efficacy. *Statistics in medicine* 2006; 25(14): 2365–2383.

131. Sato H, Hirakawa A, Hamada C. An adaptive dose-finding method using a change-point model for molecularly targeted agents in phase I trials. *Statistics in medicine* 2016; 35(23): 4093–4109.

132. Daimon T, Zohar S. An adaptive model switching approach for phase I dose-finding trials. *Pharmaceutical Statistics* 2013; 12(4): 225–232.

133. Du Y, Yin J, Sargent DJ, Mandrekar SJ. An adaptive multi-stage phase I dose-finding design incorporating continuous efficacy and toxicity data from multiple treatment cycles. *Journal of biopharmaceutical statistics* 2019; 29(2): 271–286.

134. Mandrekar SJ, Cui Y, Sargent DJ. An adaptive phase I design for identifying a biologically optimal dose for dual agent drug combinations. *Statistics in medicine* 2007; 26(11): 2317–2330.

135. Lin R, Thall PF, Yuan Y. An adaptive trial design to optimize dose-schedule regimes with delayed outcomes. *Biometrics* 2020; 76(1): 304–315.

136. Mezzetti M, Muliere P, Bulla P. An application of reinforced urn processes to determining maximum tolerated dose. *Statistics & probability letters* 2007; 77(7): 740–747.

137. Colin P, Delattre M, Minini P, Micallef S. An escalation for bivariate binary endpoints controlling the risk of Overtoxicity (EBE-CRO): managing efficacy and toxicity in early oncology clinical trials. *Journal of Biopharmaceutical Statistics* 2017; 27(6): 1054–1072.

138. Whitehead J, Zhou Y, Stevens J, Blakey G. An evaluation of a Bayesian method of dose escalation based on bivariate binary responses. *Journal of Biopharmaceutical Statistics* 2004; 14(4): 969–983.

139. Ahn C. An evaluation of phase I cancer clinical trial designs. *Statistics in medicine* 1998; 17(14): 1537–1549.

140. Storer BE. An evaluation of phase I clinical trial designs in the continuous dose–response setting. *Statistics in medicine* 2001; 20(16): 2399–2408.

141. Leung DHY, Wang YG. An extension of the continual reassessment method using decision theory. *Statistics in medicine* 2002; 21(1): 51–63.

142. Møller S. An extension of the continual reassessment methods using a preliminary up-and-down design in a dose finding study in cancer patients, in order to investigate a greater range of doses. *Statistics in medicine* 1995; 14(9): 911–922.

143. Mozgunov P, Jaki T. An information theoretic phase I–II design for molecularly targeted agents that does not require an assumption of monotonicity. *Journal of the Royal Statistical Society Series C: Applied Statistics* 2019; 68(2): 347–367.

144. Pan H, Mu R, Hsu CW, Zhou S. An R package for continuous and ordinal outcomes in Phase I dose-finding trials. *Communications for Statistical Applications and Methods* 2022; 29(4): 421–439.

145. O'Quigley J. Another look at two phase I clinical trial designs. *Statistics in medicine* 1999; 18(20): 2683–2690.

146. Yada S, Hamada C. Application of Bayesian hierarchical models for phase I/II clinical trials in oncology. *Pharmaceutical Statistics* 2017; 16(2): 114–121.

147. Yada S, Hamada C. Application of gamma process to two-agent combinations with delayed toxicity. *Communications in Statistics-Simulation and Computation* 2021; 50(1): 153–163.

148. Garrett-Mayer E. *Application of New Designs in Phase I Trials*ch. 6: 65-73; John Wiley & Sons, Ltd . 2014

149. Wages NA, Nelson B, Kharofa J, Meier T. Application of the patient-reported outcomes continual reassessment method to a phase I study of radiotherapy in endometrial cancer. *The International Journal of Biostatistics* 2023; 19(1): 163–176.

150. Jin H, Du W, Yin G. Approximate Bayesian computation design for phase I clinical trials. *Statistical methods in medical research* 2022; 31(12): 2310–2322.

151. Bartroff J, Lai TL. Approximate dynamic programming and its applications to the design of phase I cancer trials. *Statistical Science* 2010; 25(2): 245–257.

152. Hirakawa A, Sato H. Authors' Reply. *Statistics in Medicine* 2016; 35(3): 479-480. doi: https://doi.org/10.1002/sim.6751

153. Yada S. Bayesian adaptive design of early-phase clinical trials for precision medicine based on cancer biomarkers. *The International Journal of Biostatistics* 2022; 18(1): 109–125.

154. Yeung WY, Whitehead J, Reigner B, Beyer U, Diack C, Jaki T. Bayesian adaptive dose-escalation procedures for binary and continuous responses utilizing a gain function. *Pharmaceutical statistics* 2015; 14(6): 479–487.

155. Pan H, Cheng C, Yuan Y. Bayesian adaptive linearization method for phase I drug combination trials with dimension reduction. *Pharmaceutical statistics* 2020; 19(5): 561–582.

156. Lin R, Yin G, Shi H. Bayesian adaptive model selection design for optimal biological dose finding in phase I/II clinical trials. *Biostatistics* 2023; 24(2): 277–294.

157. Ji Y, Feng L, Liu P, et al. Bayesian continual reassessment method for dose-finding trials infusing T cells with limited sample size. *Journal of biopharmaceutical statistics* 2012; 22(6): 1206–1219.

158. Liu S, Yin G, Yuan Y. Bayesian data augmentation dose finding with continual reassessment method and delayed toxicity. *The annals of applied statistics* 2013; 7(4): 1837.

159. Whitehead J, Williamson D. Bayesian decision procedures based on logistic regression models for dose-finding studies. *Journal of Biopharmaceutical Statistics* 1998; 8(3): 445–467.

160. Zhou Y, Whitehead J, Bonvini E, Stevens JW. Bayesian decision procedures for binary and continuous bivariate dose-escalation studies. *Pharmaceutical Statistics: The Journal of Applied Statistics in the Pharmaceutical Industry* 2006; 5(2): 125–133.

161. Whitehead J, Zhou Y, Stevens J, Blakey G, Price J, Leadbetter J. Bayesian decision procedures for dose-escalation based on evidence of undesirable events and therapeutic benefit. *Statistics in Medicine* 2006; 25(1): 37–53.

162. Yuan Y, Yin G. Bayesian dose finding by jointly modelling toxicity and efficacy as time-to-event outcomes. *Journal of the Royal Statistical Society Series C: Applied Statistics* 2009; 58(5): 719–736.

163. Huo L, Yuan Y, Yin G. Bayesian dose finding for combined drugs with discrete and continuous doses. *Bayesian analysis (Online)* 2012; 7(4): 1035.

164. Gasparini M, Bailey S, Neuenschwander B. Bayesian dose finding in oncology for drug combinations by copula regression. *Journal of the Royal Statistical Society Series C: Applied Statistics* 2010; 59(3): 543–544.

165. Yin G, Yuan Y. Bayesian dose finding in oncology for drug combinations by copula regression. *Journal of the Royal Statistical Society Series C: Applied Statistics* 2009; 58(2): 211–224.

166. Yin G, Yuan Y. Authors' Response. *Journal of the Royal Statistical Society Series C: Applied Statistics* 2010; 59(3): 544-546. doi: 10.1111/j.1467-9876.2010.00710$_2.x$

167. Ji Y, Li Y, Yin G. Bayesian dose finding in phase I clinical trials based on a new statistical framework. *Statistica Sinica* 2007: 531–547.

168. Zhang J, Braun TM. A phase I Bayesian adaptive design to simultaneously optimize dose and schedule assignments both between and within patients. *Journal of the American Statistical Association* 2013; 108(503): 892–901.

169. Guo B, Li Y. Bayesian dose-finding designs for combination of molecularly targeted agents assuming partial stochastic ordering. *Statistics in medicine* 2015; 34(5): 859–875.

170. Yin G, Li Y, Ji Y. Bayesian dose-finding in phase I/II clinical trials using toxicity and efficacy odds ratios. *Biometrics* 2006; 62(3): 777–787.

171. Braun TM. A simulation-free approach to assessing the performance of the continual reassessment method. *Statistics in medicine* 2020; 39(30): 4651–4666.

172. Takeda K, Morita S. Bayesian dose-finding phase I trial design incorporating historical data from a preceding trial. *Pharmaceutical statistics* 2018; 17(4): 372–382.

173. Takeda K, Komatsu K, Morita S. Bayesian dose-finding phase I trial design incorporating pharmacokinetic assessment in the field of oncology. *Pharmaceutical Statistics* 2018; 17(6): 725–733.

174. Banerjee S, Bose T, Patil VM, Bhattacharjee A, Prabhash K. Bayesian Effective Biological Dose Determination in Immunotherapy Response Trial. *Annals of Data Science* 2023; 10(1): 209–223.

175. Yuan Y, Yin G. Bayesian hybrid dose-finding design in phase I oncology clinical trials. *Statistics in medicine* 2011; 30(17): 2098–2108.

176. Gatsonis C, Greenhouse JB. Bayesian methods for phase I clinical trials. *Statistics in medicine* 1992; 11(10): 1377–1389.

177. Asakawa T, Hirakawa A, Hamada C. Bayesian model averaging continual reassessment method for bivariate binary efficacy and toxicity outcomes in phase I oncology trials. *Journal of Biopharmaceutical Statistics* 2014; 24(2): 310–325.

178. Yin G, Yuan Y. Bayesian model averaging continual reassessment method in phase I clinical trials. *Journal of the American Statistical Association* 2009; 104(487): 954–968.

179. Fernandes LL, Taylor JM, Murray S. Adaptive phase I clinical trial design using Markov models for conditional probability of toxicity. *Journal of biopharmaceutical statistics* 2016; 26(3): 475–498.

180. Thall PF. Bayesian models and decision algorithms for complex early phase clinical trials. *Statistical science: a review journal of the Institute of Mathematical Statistics* 2010; 25(2): 227.

181. Haines LM, Perevozskaya I, Rosenberger WF. Bayesian optimal designs for phase I clinical trials. *Biometrics* 2003; 59(3): 591–600.

182. Altzerinakou MA, Paoletti X. An adaptive design for the identification of the optimal dose using joint modeling of continuous repeated biomarker measurements and time-to-toxicity in phase I/II clinical trials in oncology. *Statistical methods in medical research* 2020; 29(2): 508–521.

183. Lin R. Bayesian optimal interval design with multiple toxicity constraints. *Biometrics* 2018; 74(4): 1320–1330.

184. Gerard E, Zohar S, Thai HT, Lorenzato C, Riviere MK, Ursino M. Bayesian dose regimen assessment in early phase oncology incorporating pharmacokinetics and pharmacodynamics. *Biometrics* 2022; 78(1): 300–312.

185. Takahashi A, Suzuki T. Bayesian optimization design for dose-finding based on toxicity and efficacy outcomes in phase I/II clinical trials. *Pharmaceutical Statistics* 2021; 20(3): 422–439.

186. Takahashi A, Suzuki T. Bayesian optimization design for finding a maximum tolerated dose combination in phase I clinical trials. *The International Journal of Biostatistics* 2022; 18(1): 39–56.

187. Yuan Y, Yin G. Bayesian phase I/II adaptively randomized oncology trials with combined drugs. *The annals of applied statistics* 2011; 5(2A): 924.

188. Guo B, Yuan Y. Bayesian phase I/II biomarker-based dose finding for precision medicine with molecularly targeted agents. *Journal of the American Statistical Association* 2017; 112(518): 508–520.

189. Whitehead J, Thygesen H, Whitehead A. Bayesian procedures for phase I/II clinical trials investigating the safety and efficacy of drug combinations. *Statistics in Medicine* 2011; 30(16): 1952–1970.

190. Li M, Liu R, Lin J, Bunn V, Zhao H. Bayesian semi-parametric design (BSD) for adaptive dose-finding with multiple strata. *Journal of biopharmaceutical statistics* 2020; 30(5): 806–820.

191. Xu J, Yin G, Ohlssen D, Bretz F. Bayesian two-stage dose finding for cytostatic agents via model adaptation. *Journal of the Royal Statistical Society Series C: Applied Statistics* 2016; 65(3): 465–482.

192. Domenicano I, Ventz S, Cellamare M, Mak RH, Trippa L. Bayesian uncertainty-directed dose finding designs. *Journal of the Royal Statistical Society Series C: Applied Statistics* 2019; 68(5): 1393–1410.

193. Sweeting M, Mander A, Sabin T. Bcrm: Bayesian continual reassessment method designs for phase I dose-finding trials. *Journal of Statistical Software* 2013; 54: 1–26.

194. Guo B, Zang Y. BIPSE: A biomarker-based phase I/II design for immunotherapy trials with progression-free survival endpoint. *Statistics in medicine* 2022; 41(7): 1205–1224.

195. Ivanova A, Wang K. Bivariate isotonic design for dose-finding with ordered groups. *Statistics in Medicine* 2006; 25(12): 2018–2026.

196. Lee J, Thall PF, Ji Y, Müller P. Bayesian dose-finding in two treatment cycles based on the joint utility of efficacy and toxicity. *Journal of the American Statistical Association* 2015; 110(510): 711–722.

197. Takeda K, Taguri M, Morita S. BOIN-ET: Bayesian optimal interval design for dose finding based on both efficacy and toxicity outcomes. *Pharmaceutical statistics* 2018; 17(4): 383–395.

198. Lin R, Yin G. Bootstrap aggregating continual reassessment method for dose finding in drug-combination trials. *The Annals of Applied Statistics* 2016; 10(4): 2349–2376.

199. Chen X, Zhang J, Jiang Q, Yan F. Borrowing historical information to improve phase I clinical trials using meta-analytic-predictive priors. *Journal of Biopharmaceutical Statistics* 2022; 32(1): 34–52.

200. Zheng H, Hampson LV, Jaki T. Bridging across patient subgroups in phase I oncology trials that incorporate animal data. *Statistical Methods in Medical Research* 2021; 30(4): 1057–1071.

201. Liu S, Pan H, Xia J, Huang Q, Yuan Y. Bridging continual reassessment method for phase I clinical trials in different ethnic populations. *Statistics in Medicine* 2015; 34(10): 1681–1694.

202. O'Quigley J, Iasonos A. Bridging solutions in dose-finding problems. *Statistics in biopharmaceutical research* 2014; 6(2): 185–197.

203. Lee SM, Cheung YK. Calibration of prior variance in the Bayesian continual reassessment method. *Statistics in medicine* 2011; 30(17): 2081–2089.

204. Babb J, Rogatko A, Zacks S. Cancer phase I clinical trials: efficient dose escalation with overdose control. *Statistics in medicine* 1998; 17(10): 1103–1120.

205. Jimenez JL, Tighiouart M, Gasparini M. Cancer phase I trial design using drug combinations when a fraction of dose limiting toxicities is attributable to one or more agents. *Biometrical Journal* 2019; 61(2): 319–332.

206. Jin H, Yin G. CFO: Calibration-free odds design for phase I/II clinical trials. *Statistical Methods in Medical Research* 2022; 31(6): 1051–1066.

207. Altzerinakou MA, Paoletti X. Change-point joint model for identification of plateau of activity in early phase trials. *Statistics in Medicine* 2021; 40(9): 2113–2138.

208. Jia X, Lee SM, Cheung YK. Characterization of the likelihood continual reassessment method. *Biometrika* 2014; 101(3): 599–612.

209. Gerard E, Zohar S, Lorenzato C, Ursino M, Riviere Mk. Bayesian modeling of a bivariate toxicity outcome for early phase oncology trials evaluating dose regimens. *Statistics in Medicine* 2021; 40(23): 5096–5114.

210. Cheung YKK. Coherence in Phase I Clinical Trials. *Methods and Applications of Statistics in Clinical Trials: Concepts, Principles, Trials, and Design* 2014: 230–239.

211. Cheung YK. Coherence principles in dose-finding studies. *Biometrika* 2005; 92(4): 863–873.

212. Wages NA, Iasonos A, O'Quigley J, Conaway MR. Coherence principles in interval-based dose finding. *Pharmaceutical statistics* 2020; 19(2): 137–144.

213. Alam MI, Coad DS, Bogacka B. Combined criteria for dose optimisation in early phase clinical trials. *Statistics in Medicine* 2019; 38(21): 4172–4188.

214. Jiménez JL, Tighiouart M. Combining cytotoxic agents with continuous dose levels in seamless phase I-II clinical trials. *Journal of the Royal Statistical Society Series C: Applied Statistics* 2022; 71(5): 1996–2013.

215. Norris DC. Comment on Wages et al, Coherence principles in interval-based dose finding. Pharmaceutical Statistics 2019, doi:10.1002/pst.1974. *Pharmaceutical Statistics* 2020; 19(5): 741-742. doi: https://doi.org/10.1002/pst.2016

216. Yuan Y, Zhao Y. Commentary on "Improving the performance of Bayesian logistic regression model with overdose control in oncology dose-finding studies". *Statistics in Medicine* 2022; 41(27): 5484–5490.

217. Li X, Liu K, Tian H. Comments on" Critical aspects of the Bayesian approach to phase I cancer trials". *Statistics in medicine* 2020; 39(27): 4100.

218. Riviere MK, Zohar S. Comments on 'A comparative study of adaptive dose-finding designs for phase I oncology trials of combination therapies'. *Statistics in Medicine* 2016; 35(3): 475–478.

219. Wages NA, Conaway MR, O'Quigley J. Comments on 'A dose-finding approach based on shrunken predictive probability for combinations of two agents in phase I trials' by Akihiro Hirakawa, Chikuma Hamada, and Shigeyuki Matsui. *Statistics in Medicine* 2014; 33(12): 2156-2158. doi: https://doi.org/10.1002/sim.5934

220. Yin G, Lin R. Comments on 'Competing designs for drug combination in phase I dose-finding clinical trials' by M-K. Riviere, F. Dubois, and S. Zohar. *Statistics in Medicine* 2015; 34(1): 13-17. doi: https://doi.org/10.1002/sim.6338

221. Wages NA. Comments on 'Competing designs for drug combination in phase I dose-finding clinical trials' by M-K. Riviere, F. Dubois, S. Zohar. *Statistics in Medicine* 2015; 34(1): 18-22. doi: https://doi.org/10.1002/sim.6336

222. Lin R, Yin G. Bayesian optimal interval design for dose finding in drug-combination trials. *Statistical methods in medical research* 2017; 26(5): 2155–2167.

223. Riviere MK, Dubois F, Zohar S. Competing designs for drug combination in phase I dose-finding clinical trials. *Statistics in medicine* 2015; 34(1): 1–12.

224. Rosenberger WF, Haines LM. Competing designs for phase I clinical trials: a review. *Statistics in medicine* 2002; 21(18): 2757–2770.

225. Biswas A, López-Fidalgo J. Compound designs for dose-finding in the presence of nondesignable covariates. *Pharmaceutical statistics* 2013; 12(2): 92–101.

226. Shen LZ, O'QUIGLEY J. Consistency of continual reassessment method under model misspecification. *Biometrika* 1996; 83(2): 395–405.

227. Saha PT, Fine JP, Ivanova A. Consistency of the CRM when the dose-toxicity curve is not monotone and its application to the POCRM. *Statistics in Medicine* 2021; 40(8): 2073–2082.

228. O'Quigley J, Conaway M. Continual reassessment and related dose-finding designs. *Statistical science: a review journal of the Institute of Mathematical Statistics* 2010; 25(2): 202.

229. O'Quigley J. Continual reassessment designs with early termination. *Biostatistics* 2002; 3(1): 87–99.

230. O'Quigley J, Paoletti X. Continual reassessment method for ordered groups. *Biometrics* 2003; 59(2): 430–440.

231. Wages NA, Conaway MR, O'Quigley J. Continual reassessment method for partial ordering. *Biometrics* 2011; 67(4): 1555–1563.

232. Onar A, Kocak M, Boyett JM. Continual reassessment method vs. traditional empirically based design: modifications motivated by Phase I trials in pediatric oncology by the Pediatric Brain Tumor Consortium. *Journal of biopharmaceutical statistics* 2009; 19(3): 437–455.

233. Lee SM, Cheng B, Cheung YK. Continual reassessment method with multiple toxicity constraints. *Biostatistics* 2011; 12(2): 386–398.

234. Li X, Ivanova A, Tian H, Lim P, Liu K. Continual reassessment method with regularization in phase I clinical trials. *Journal of biopharmaceutical statistics* 2020; 30(6): 964–978.

235. O'Quigley J, Shen LZ. Continual reassessment method: a likelihood approach. *Biometrics* 1996: 673–684.

236. O'Quigley J, Pepe M, Fisher L. Continual reassessment method: a practical design for phase 1 clinical trials in cancer. *Biometrics* 1990: 33–48.

237. Kramar A, Lebecq A, Candalh E. Continual reassessment methods in phase I trials of the combination of two drugs in oncology. *Statistics in medicine* 1999; 18(14): 1849–1864.

238. Neuenschwander B, Branson M, Gsponer T. Critical aspects of the Bayesian approach to phase I cancer trials. *Statistics in medicine* 2008; 27(13): 2420–2439.

239. Ivanova A, Flournoy N, Chung Y. Cumulative cohort design for dose-finding. *Journal of Statistical Planning and Inference* 2007; 137(7): 2316–2327.

240. O'Quigley J. Curve-free and model-based continual reassessment method designs. *Biometrics* 2002; 58(1): 245–249.

241. Hatayama T, Yasui S. CUSUMIN: A cumulative sum interval design for cancer phase I dose finding studies. *Pharmaceutical Statistics* 2022; 21(6): 1324–1341.

242. Zhang Y, Zang Y. CWL: A conditional weighted likelihood method to account for the delayed joint toxicity–efficacy outcomes for phase I/II clinical trials. *Statistical Methods in Medical Research* 2021; 30(3): 892–903.

243. Fan SK, Wang YG. Decision-theoretic designs for dose-finding clinical trials with multiple outcomes. *Statistics in medicine* 2006; 25(10): 1699–1714.

244. Stylianou M. Derivation of exact distributions following an up-and-down design. *Communications in Statistics-Theory and Methods* 2009; 38(16-17): 3190–3197.

245. Paoletti X, O'Quigley J, Maccario J. Design efficiency in dose finding studies. *Computational statistics & data analysis* 2004; 45(2): 197–214.

246. Fan SK, Venook AP, Lu Y. Design issues in dose-finding phase I trials for combinations of two agents. *Journal of Biopharmaceutical Statistics* 2009; 19(3): 509–523.

247. Wang C, Chen TT, Tyan I. Designs foe phase I cancer clinical trials with differentiation of graded toxicity. *Communications in Statistics-Theory and Methods* 2000; 29(5-6): 975–987.

248. Fan SK, Wang YG. Designs for phase I clinical trials with multiple courses of subjects at different doses. *Biometrics* 2007; 63(3): 856–864.

249. Conaway MR, Wages NA. Designs for phase I trials in ordered groups. *Statistics in medicine* 2017; 36(2): 254–265.

250. Conaway MR, Dunbar S, Peddada SD. Designs for single-or multiple-agent phase I trials. *Biometrics* 2004; 60(3): 661–669.

251. Liu S, Yuan Y. Bayesian optimal interval designs for phase I clinical trials. *Journal of the Royal Statistical Society: Series C: Applied Statistics* 2015: 507–523.

252. Mason AJ, Gonzalez-Maffe J, Quinn K, et al. Developing a Bayesian adaptive design for a phase I clinical trial: a case study for a novel HIV treatment. *Statistics in medicine* 2017; 36(5): 754–771.

253. Ursino M, Biard L, Chevret S. DICE: A Bayesian model for early dose finding in phase I trials with multiple treatment courses. *Biometrical Journal* 2022; 64(8): 1486–1497.

254. Iasonos A, Wages NA, Conaway MR, Cheung K, Yuan Y, O'Quigley J. Dimension of model parameter space and operating characteristics in adaptive dose-finding studies. *Statistics in medicine* 2016; 35(21): 3760–3775.

255. Hardwick J, Meyer MC, Stout QF. Directed walk designs for dose-response problems with competing failure modes. *Biometrics* 2003; 59(2): 229–236.

256. Hunsberger S, Rubinstein LV, Dancey J, Korn EL. Dose escalation trial designs based on a molecularly targeted endpoint. *Statistics in medicine* 2005; 24(14): 2171–2181.

257. Fedorov VV, Wu Y. Dose finding designs for continuous responses and binary utility. *Journal of biopharmaceutical statistics* 2007; 17(6): 1085–1096.

258. Ivanova A, Kim SH. Dose finding for continuous and ordinal outcomes with a monotone objective function: a unified approach. *Biometrics* 2009; 65(1): 307–315.

259. Yan F, Zhang L, Zhou Y, Pan H, Liu S, Yuan Y. BOIN: an R package for designing single-agent and drug-combination dose-finding trials using Bayesian optimal interval designs. *Journal of Statistical Software* 2020; 94: 1–32.

260. Stylianou M, Flournoy N. Dose finding using the biased coin up-and-down design and isotonic regression. *Biometrics* 2002; 58(1): 171–177.

261. Ivanova A, Xiao C. Dose finding when the target dose is on a plateau of a dose–response curve: comparison of fully sequential designs. *Pharmaceutical statistics* 2013; 12(5): 309–314.

262. Wang Y, Ivanova A. Dose finding with continuous outcome in phase I oncology trials. *Pharmaceutical statistics* 2015; 14(2): 102–107.

263. Tighiouart M, Piantadosi S, Rogatko A. Dose finding with drug combinations in cancer phase I clinical trials using conditional escalation with overdose control. *Statistics in medicine* 2014; 33(22): 3815–3829.

264. Tighiouart M, Rogatko A. Dose finding with escalation with overdose control (EWOC) in cancer clinical trials. *Statistical Science* 2010; 25(2): 217–226.

265. Paoletti X, Doussau A, Ezzalfani M, Rizzo E, Thiébaut R. Dose finding with longitudinal data: simpler models, richer outcomes. *Statistics in medicine* 2015; 34(22): 2983–2998.

266. Kakurai Y, Kaneko S, Hamada C, Hirakawa A. Dose individualization and variable selection by using the Bayesian lasso in early phase dose finding trials. *Journal of the Royal Statistical Society Series C: Applied Statistics* 2019; 68(2): 445–460.

267. Xu C, Zhuo B, Rasmussen HE. Dose intra-subject escalation to an event (DIETE): A new method for phase 1 dose-finding utilizing systematic intra-subject dose escalation with application to T-cell engagers. *Pharmaceutical Statistics* 2021; 20(6): 1200–1215.

268. Shu J, O'Quigley J. Dose-escalation designs in oncology: ADEPT and the CRM. *Statistics in Medicine* 2008; 27(26): 5345–5353.

269. Mauguen A, Le Deley M, Zohar S. Dose-finding approach for dose escalation with overdose control considering incomplete observations. *Statistics in medicine* 2011; 30(13): 1584–1594.

270. Thall PF, Cook JD. Dose-finding based on efficacy–toxicity trade-offs. *Biometrics* 2004; 60(3): 684–693.

271. Thall PF, Sung HG, Choudhury A. Dose-finding based on feasibility and toxicity in T-cell infusion trials. *Biometrics* 2001; 57(3): 914–921.

272. Bekele BN, Thall PF. Dose-finding based on multiple toxicities in a soft tissue sarcoma trial. *Journal of the American Statistical Association* 2004; 99(465): 26–35.

273. Andrillon A, Chevret S, Lee SM, Biard L. Dose-finding design and benchmark for a right censored endpoint. *Journal of biopharmaceutical statistics* 2020; 30(6): 948–963.

274. Seegers V, Chevret S, Resche-Rigon M. Dose-finding design driven by efficacy in onco-hematology phase I/II trials. *Statistics in Medicine* 2011; 30(13): 1574–1583.

275. Yin J, Yuan Y. Checkerboard: a Bayesian efficacy and toxicity interval design for phase I/II dose-finding trials. *Journal of Biopharmaceutical Statistics* 2020; 30(6): 1006–1025.

276. Lee SM, Ursino M, Cheung YK, Zohar S. Dose-finding designs for cumulative toxicities using multiple constraints. *Biostatistics* 2019; 20(1): 17–29.

277. O'Quigley J, Hughes MD, Fenton T. Dose-finding designs for HIV studies. *Biometrics* 2001; 57(4): 1018–1029.

278. Chiuzan C, Shtaynberger J, Manji GA, et al. Dose-finding designs for trials of molecularly targeted agents and immunotherapies. *Journal of biopharmaceutical statistics* 2017; 27(3): 477–494.

279. Ezzalfani M, Zohar S, Qin R, Mandrekar SJ, Deley MCL. Dose-finding designs using a novel quasi-continuous endpoint for multiple toxicities. *Statistics in medicine* 2013; 32(16): 2728–2746.

280. Oron AP, Azriel D, Hoff PD. Dose-finding designs: the role of convergence properties. *The international journal of biostatistics* 2011; 7(1): 0000102202155746791298.

281. Ursino M, Zohar S, Lentz F, et al. Dose-finding methods for phase I clinical trials using pharmacokinetics in small populations. *Biometrical Journal* 2017; 59(4): 804–825.

282. Thall PF, Millikan RE, Mueller P, Lee SJ. Dose-finding with two agents in phase I oncology trials. *Biometrics* 2003; 59(3): 487–496.

283. Li Y, Bekele BN, Ji Y, Cook JD. Dose–schedule finding in phase I/II clinical trials using a Bayesian isotonic transformation. *Statistics in medicine* 2008; 27(24): 4895–4913.

284. Zhang T, Yang Z, Yin G. Dynamic ordering design for dose finding in drug-combination trials. *Pharmaceutical Statistics* 2021; 20(2): 348–361.

285. Kojima M. Early completion of phase I cancer clinical trials with Bayesian optimal interval design. *Statistics in Medicine* 2021; 40(14): 3215–3226.

286. Khan NM, Alam MI. Early stopping in seamless phase I/II clinical trials. *Pharmaceutical Statistics* 2021; 20(2): 390–412.

287. Hirakawa A, Sato H, Gosho M. Effect of design specifications in dose-finding trials for combination therapies in oncology. *Pharmaceutical Statistics* 2016; 15(6): 531–540.

288. Liu Q, Geng J, Fleischer F, Deng Q. Efficacy-Driven Dose Finding with Toxicity Control in Phase I Oncology Studies. *Statistics in Biosciences* 2022: 1–19.

289. Sweeting MJ, Mander AP. Escalation strategies for combination therapy Phase I trials. *Pharmaceutical statistics* 2012; 11(3): 258–266.

290. Shi Y, Yin G. Escalation with overdose control for phase I drug-combination trials. *Statistics in medicine* 2013; 32(25): 4400–4412.

291. Tighiouart M, Cook-Wiens G, Rogatko A. Escalation with Overdose Control Using Ordinal Toxicity Grades for Cancer Phase I Clinical Trials. *Journal of Probability and Statistics* 2012; 2012(1): 317634. doi: https://doi.org/10.1155/2012/317634

292. Ivanova A. Escalation, group and A+ B designs for dose-finding trials. *Statistics in Medicine* 2006; 25(21): 3668–3678.

293. O'Quigley J. Estimating the probability of toxicity at the recommended dose following a phase I clinical trial in cancer. *Biometrics* 1992: 853–862.

294. Stylianou M, Proschan M, Flournoy N. Estimating the probability of toxicity at the target dose following an up-and-down design. *Statistics in medicine* 2003; 22(4): 535–543.

295. Morita S, Thall PF, Müller P. Evaluating the impact of prior assumptions in Bayesian biostatistics. *Statistics in biosciences* 2010; 2: 1–17.

296. Wang S, Sayour E, Lee JH. Evaluation of phase I clinical trial designs for combinational agents along with guidance based on simulation studies. *Journal of Applied Statistics* 2023; 50(9): 2055–2078.

297. O'Quigley J, Conaway M. Extended model-based designs for more complex dose-finding studies. *Statistics in Medicine* 2011; 30(17): 2062–2069.

298. Zhou H, Murray TA, Pan H, Yuan Y. Comparative review of novel model-assisted designs for phase I clinical trials. *Statistics in Medicine* 2018; 37(14): 2208–2222.

299. Tighiouart M, Rogatko A, Babb JS. Flexible Bayesian methods for cancer phase I clinical trials. Dose escalation with overdose control. *Statistics in medicine* 2005; 24(14): 2183–2196.

300. Zhong W, Carlin BP, Koopmeiners JS. Flexible link continual reassessment methods for trivariate binary outcome phase I/II trials. *Journal of Statistical Theory and Practice* 2013; 7: 442–455.

301. Potthoff RF, George SL. Flexible phase I clinical trials: allowing for nonbinary toxicity response and removal of other common limitations. *Statistics in biopharmaceutical research* 2009; 1(3): 213–228.

302. Wages NA, Slingluff Jr CL. Flexible Phase I–II Design for Partially Ordered Regimens with Application to Therapeutic Cancer Vaccines. *Statistics in biosciences* 2020; 12(2): 104–123.

303. Hashizume K, Tshuchida J, Sozu T. Flexible use of copula-type model for dose-finding in drug combination clinical trials. *Biometrics* 2022; 78(4): 1651–1661.

304. Yin G, Zheng S, Xu J. Fractional dose-finding methods with late-onset toxicity in phase I clinical trials. *Journal of Biopharmaceutical Statistics* 2013; 23(4): 856–870.

305. Mu R, Yuan Y, Xu J, Mandrekar SJ, Yin J. gBOIN: a unified model-assisted phase I trial design accounting for toxicity grades, and binary or continuous end points. *Journal of the Royal Statistical Society Series C: Applied Statistics* 2019; 68(2): 289–308.

306. Takeda K, Morita S, Taguri M. gBOIN-ET: The generalized Bayesian optimal interval design for optimal dose-finding accounting for ordinal graded efficacy and toxicity in early clinical trials. *Biometrical Journal* 2022; 64(7): 1178–1191.

307. Gasparini M. General classes of multiple binary regression models in dose finding problems for combination therapies. *Journal of the Royal Statistical Society Series C: Applied Statistics* 2013; 62(1): 115–133.

308. Yan D, Tait C, Wages NA, Kindwall-Keller T, Dressler EV. Generalization of the time-to-event continual reassessment method to bivariate outcomes. *Journal of biopharmaceutical statistics* 2019; 29(4): 635–647.

309. Braun TM. Generalizing the TITE-CRM to adapt for early-and late-onset toxicities. *Statistics in medicine* 2006; 25(12): 2071–2083.

310. Gezmu M, Flournoy N. Group up-and-down designs for dose-finding. *Journal of statistical planning and inference* 2006; 136(6): 1749–1764.

311. Legedza AT, Ibrahim JG. Heterogeneity in phase I clinical trials: prior elicitation and computation using the continual reassessment method. *Statistics in Medicine* 2001; 20(6): 867–882.

312. Cunanan KM, Koopmeiners JS. Hierarchical models for sharing information across populations in phase I dose-escalation studies. *Statistical methods in medical research* 2018; 27(11): 3447–3459.

313. Carter L, O'Brien C, Dean E, Cook N. How to Design Phase I Trials in Oncology. *Methods and Biostatistics in Oncology: Understanding Clinical Research as an Applied Tool* 2018: 165–187.

314. Ghosh D, Xie H, Zhang L, Chen F, Mohanty S, Li X. Hybrid continuous reassessment method with overdose control for safer dose escalation. *Journal of Biopharmaceutical Statistics* 2023; 33(5): 586–595.

315. Wages NA. Identifying a maximum tolerated contour in two-dimensional dose finding. *Statistics in medicine* 2017; 36(2): 242–253.

316. Zohar S, O'Quigley J. Identifying the most successful dose (MSD) in dose-finding studies in cancer. *Pharmaceutical Statistics: The Journal of Applied Statistics in the Pharmaceutical Industry* 2006; 5(3): 187–199.

317. Ulas E, Karaman F, Koc T. Impact of different model structure and prior distribution in continual reassessment method. *Communications in Statistics-Simulation and Computation* 2020; 49(1): 95–109.

318. Petroni GR, Wages NA, Paux G, Dubois F. Implementation of adaptive methods in early-phase clinical trials. *Statistics in medicine* 2017; 36(2): 215–224.

319. Yan D, Wages NA, Dressler EV. Improved adaptive randomization strategies for a seamless phase I/II dose-finding design. *Journal of Biopharmaceutical Statistics* 2019; 29(2): 333–347.

320. Piantadosi S, Liu G. Improved designs for dose escalation studies using pharmacokinetic measurements. *Statistics in medicine* 1996; 15(15): 1605–1618.

321. Ivanova A, Montazer-Haghighi A, Mohanty SG, D. Durham S. Improved up-and-down designs for phase I trials. *Statistics in medicine* 2003; 22(1): 69–82.

322. Ji L, Lewinger JP, Krailo M, et al. Improvements to the Escalation with Overdose Control design and a comparison with the restricted Continual Reassessment Method. *Pharmaceutical statistics* 2019; 18(6): 659–670.

323. Zeng Z, Chen C, Zhou H, Wang M. Improving Dose-Finding for New Agents as Monotherapy and Add-On Therapy. *Statistics in Biopharmaceutical Research* 2021; 13(4): 461–467.

324. Mozgunov P, Jaki T. Improving safety of the continual reassessment method via a modified allocation rule. *Statistics in medicine* 2020; 39(7): 906–922.

325. Ivanova A, Flournoy N. Comparison of isotonic designs for dose-finding. *Statistics in Biopharmaceutical Research* 2009; 1(1): 101–107.

326. Wheeler GM. Incoherent dose-escalation in phase I trials using the escalation with overdose control approach. *Statistical Papers* 2018; 59(2): 801–811.

327. Tighiouart M, Cook-Wiens G, Rogatko A. Incorporating a patient dichotomous characteristic in cancer phase I clinical trials using escalation with overdose control. *Journal of Probability and Statistics* 2012; 2012(1): 567819.

328. Darssan D, Thompson MH, Pettitt AN. Incorporating adverse event relatedness into dose-finding clinical trial designs. *Statistics in Medicine* 2014; 33(7): 1146–1161.

329. Zhao Y, Liu R, Takeda K. Incorporating historical information to improve dose optimization design with toxicity and efficacy endpoints: iBOIN-ET. *Pharmaceutical Statistics* 2023; 22(3): 440–460.

330. Zhou Y, Lee JJ, Wang S, Bailey S, Yuan Y. Incorporating historical information to improve phase I clinical trials. *Pharmaceutical statistics* 2021; 20(6): 1017–1034.

331. Bartroff J, Leung Lai T. Incorporating individual and collective ethics into phase I cancer trial designs. *Biometrics* 2011; 67(2): 596–603.

332. Lee SM, Lu X, Cheng B. Incorporating patient-reported outcomes in dose-finding clinical trials. *Statistics in medicine* 2020; 39(3): 310–325.

333. Das I, Mukhopadhyay S, Xu H. Individualized Dosing for Multiple Ordered Groups of Patients. *Journal of Statistical Theory and Practice* 2013; 7: 95–106.

334. Iasonos A, O'Quigley J. Interplay of priors and skeletons in two-stage continual reassessment method. *Statistics in medicine* 2012; 31(30): 4321–4336.

335. Natarajan L, O'Quigley J. Interval estimates of the probability of toxicity at the maximum tolerated dose for small samples. *Statistics in medicine* 2003; 22(11): 1829–1836.

336. Wages NA, Braun TM, Conaway MR. Isotonic design for phase I cancer clinical trials with late-onset toxicities. *Journal of Biopharmaceutical Statistics* 2023; 33(3): 357–370.

337. Alam MI, Sultana J. Isotonic Design for Phase I Clinical Trials: Can We Improve Further? Isotonic design for phase I clinical trials. *Austrian Journal of Statistics* 2019; 48(5): 34–44.

338. Ezzalfani M, Burzykowski T, Paoletti X. Joint modelling of a binary and a continuous outcome measured at two cycles to determine the optimal dose. *Journal of the Royal Statistical Society Series C: Applied Statistics* 2019; 68(2): 369–384.

339. Murray TA. Logistic retainment interval dose exploration design for Phase I clinical trials of cytotoxic agents. *Pharmaceutical statistics* 2021; 20(4): 850–863.

340. Hadjinicola G, Goldstein L. Markov chain modelling of bioassay toxicity procedures. *Statistics in medicine* 1993; 12(7): 661–674.

341. O'Quigley J, Chevret S. Methods for dose finding studies in cancer clinical trials: a review and results of a Monte Carlo study. *Statistics in medicine* 1991; 10(11): 1647–1664.

342. Dehbi HM, O'Quigley J, Iasonos A. Controlled amplification in oncology dose-finding trials. *Contemporary clinical trials* 2023; 125: 107021.

343. Mandrekar SJ, Qin R, Sargent DJ. Model-based phase I designs incorporating toxicity and efficacy for single and dual agent drug combinations: methods and challenges. *Statistics in medicine* 2010; 29(10): 1077–1083.

344. Wheeler GM, Sweeting MJ, Mander AP, Lee SM, Cheung YKK. Modelling semi-attributable toxicity in dual-agent phase I trials with non-concurrent drug administration. *Statistics in medicine* 2017; 36(2): 225–241.

345. Bekele BN, Ji Y, Shen Y, Thall PF. Monitoring late-onset toxicities in phase I trials using predicted risks. *Biostatistics* 2008; 9(3): 442–457.

346. Braun TM. Motivating sample sizes in adaptive phase I trials via Bayesian posterior credible intervals. *Biometrics* 2018; 74(3): 1065–1071.

347. Jin L, Pang G, Alemayehu D. Multiarmed bandit designs for phase I dose-finding clinical trials with multiple toxicity types. *Statistics in Biopharmaceutical Research* 2023; 15(1): 164–177.

348. Lam CK, Lin R, Yin G. Non-parametric overdose control for dose finding in drug combination trials. *Journal of the Royal Statistical Society Series C: Applied Statistics* 2019; 68(4): 1111–1130.

349. Lin R, Yin G. Nonparametric overdose control with late-onset toxicity in phase I clinical trials. *Biostatistics* 2017; 18(1): 180–194.

350. Huang B, Bycott P, Talukder E. Novel dose-finding designs and considerations on practical implementations in oncology clinical trials. *Journal of biopharmaceutical statistics* 2017; 27(1): 44–55.

351. Tighiouart M, Rogatko A. Number of patients per cohort and sample size considerations using dose escalation with overdose control. *Journal of Probability and Statistics* 2012; 2012(1): 692725.

352. Aziz M, Kaufmann E, Riviere MK. On multi-armed bandit designs for dose-finding trials. *Journal of Machine Learning Research* 2021; 22(14): 1–38.

353. Cheng B, Lee SM. On the consistency of the continual reassessment method with multiple toxicity constraints. *Journal of Statistical Planning and Inference* 2015; 164: 1–9.

354. Zocholl D, Wiesenfarth M, Rauch G, Kopp-Schneider A. On the feasibility of pediatric dose-finding trials in small samples with information from a preceding trial in adults. *Journal of Biopharmaceutical Statistics* 2022; 32(5): 652–670.

355. Lin R, Yuan Y. On the relative efficiency of model-assisted designs: a conditional approach. *Journal of biopharmaceutical statistics* 2019; 29(4): 648–662.

356. Cheung YK. On the use of nonparametric curves in phase I trials with low toxicity tolerance. *Biometrics* 2002; 58(1): 237–240.

357. Zacks S, Rogatko A, Babb J. Optimal Bayesian-feasible dose escalation for cancer phase I trials. *Statistics & probability letters* 1998; 38(3): 215–220.

358. Guo B, Liu S. Optimal benchmark for evaluating drug-combination dose-finding clinical trials. *Statistics in Biosciences* 2018; 10: 184–201.

359. Zohar S, O'Quigley J. Optimal designs for estimating the most successful dose. *Statistics in medicine* 2006; 25(24): 4311–4320.

360. Matsuura K, Sakamaki K, Honda J, Sozu T. Optimal dose escalation methods using deep reinforcement learning in phase I oncology trials. *Journal of biopharmaceutical statistics* 2023; 33(5): 639–652.

361. Fedorov V, Wu Y, Zhang R. Optimal dose-finding designs with correlated continuous and discrete responses. *Statistics in medicine* 2012; 31(3): 217–234.

362. Gerke O, Siedentop H. Optimal phase I dose-escalation trial designs in oncology—A simulation study. *Statistics in Medicine* 2008; 27(26): 5329–5344.

363. Azriel D. Optimal sequential designs in phase I studies. *Computational Statistics & Data Analysis* 2014; 71: 288–297.

364. Lee J, Thall PF, Rezvani K. Optimizing natural killer cell doses for heterogeneous cancer patients on the basis of multiple event times. *Journal of the Royal Statistical Society Series C: Applied Statistics* 2019; 68(2): 461–474.

365. Dehbi HM, O'Quigley J, Iasonos A. Controlled backfill in oncology dose-finding trials. *Contemporary Clinical Trials* 2021; 111: 106605.

366. Thall PF, Szabo A, Nguyen HQ, Amlie-Lefond CM, Zaidat OO. Optimizing the concentration and bolus of a drug delivered by continuous infusion. *Biometrics* 2011; 67(4): 1638–1646.

367. Li Y, Yuan Y. PA-CRM: A continuous reassessment method for pediatric phase I oncology trials with concurrent adult trials. *Biometrics* 2020; 76(4): 1364–1373.

368. Thall PF, Nguyen HQ, Zinner RG. Parametric dose standardization for optimizing two-agent combinations in a phase I–II trial with ordinal outcomes. *Journal of the Royal Statistical Society Series C: Applied Statistics* 2017; 66(1): 201–224.

369. Braun TM, Yuan Z, Thall PF. Determining a maximum-tolerated schedule of a cytotoxic agent. *Biometrics* 2005; 61(2): 335–343.

370. Babb JS, Rogatko A. Patient specific dosing in a cancer phase I clinical trial. *Statistics in medicine* 2001; 20(14): 2079–2090.

371. Thall PF, Nguyen HQ, Estey EH. Patient-specific dose finding based on bivariate outcomes and covariates. *Biometrics* 2008; 64(4): 1126–1136.

372. Moni MM, Alam MI. Patient-specific dose finding in phase I clinical trials. *Journal of Applied Statistics* 2018; 45(14): 2607–2618.

373. Labrenz J, Edelmann D, Heitmann JS, Salih HR, Kopp-Schneider A, Schlenk RF. Performance of phase-I dose finding designs with and without a run-in intra-patient dose escalation stage. *Pharmaceutical statistics* 2023; 22(2): 236–247.

374. Horton BJ, Wages NA, Conaway MR. Performance of toxicity probability interval based designs in contrast to the continual reassessment method. *Statistics in medicine* 2017; 36(2): 291–300.

375. Schipper MJ, Taylor JM, TenHaken R, Matuzak MM, Kong FM, Lawrence TS. Personalized dose selection in radiation therapy using statistical models for toxicity and efficacy with dose and biomarkers as covariates. *Statistics in medicine* 2014; 33(30): 5330–5339.

376. Alam MI, Bogacka B, Coad DS. Pharmacokinetically guided optimum adaptive dose selection in early phase clinical trials. *Computational Statistics & Data Analysis* 2017; 111: 183–202.

377. Zohar S, Chevret S. Phase I (or phase II) dose-ranging clinical trials: proposal of a two-stage Bayesian design. *Journal of Biopharmaceutical Statistics* 2003; 13(1): 87–101.

378. Ivanova A, Flournoy N. *Phase I Clinical Trials*ch. 1: 1-14; John Wiley & Sons, Ltd . 2007

379. Devlin SM, Iasonos A, O'Quigley J. Phase I clinical trials in adoptive T-cell therapies. *Journal of the Royal Statistical Society Series C: Applied Statistics* 2021; 70(4): 815–834.

380. Wages NA, O'Quigley J, Conaway MR. Phase I design for completely or partially ordered treatment schedules. *Statistics in medicine* 2014; 33(4): 569–579.

381. Iasonos A, O'Quigley J. Phase I designs that allow for uncertainty in the attribution of adverse events. *Journal of the Royal Statistical Society Series C: Applied Statistics* 2017; 66(5): 1015–1030.

382. Potter DM. Phase I studies of chemotherapeutic agents in cancer patients: a review of the designs. *Journal of Biopharmaceutical statistics* 2006; 16(5): 579–604.

383. Jin IH, Huo L, Yin G, Yuan Y. Phase I trial design for drug combinations with Bayesian model averaging. *Pharmaceutical statistics* 2015; 14(2): 108–119.

384. Chevret S. *Phase I Trials*ch. 56: 682-691; John Wiley & Sons, Ltd . 2014

385. Ivanova A. *Phase I Trials in Oncology*ch. 60: 719-724; John Wiley & Sons, Ltd . 2014

386. Wages NA, Conaway MR. phase I/II adaptive design for drug combination oncology trials. *Statistics in medicine* 2014; 33(12): 1990–2003.

387. Zohar S. *Phase I/II Clinical Trials*ch. 54: 658-666; John Wiley & Sons, Ltd . 2014

388. Riviere MK, Yuan Y, Jourdan JH, Dubois F, Zohar S. Phase I/II dose-finding design for molecularly targeted agent: plateau determination using adaptive randomization. *Statistical methods in medical research* 2018; 27(2): 466–479.

389. Muenz DG, Taylor JM, Braun TM. Phase I–II trial design for biologic agents using conditional auto-regressive models for toxicity and efficacy. *Journal of the Royal Statistical Society Series C: Applied Statistics* 2019; 68(2): 331–345.

390. Barnett H, Boix O, Kontos D, Jaki T. Dose finding studies for therapies with late-onset toxicities: A comparison study of designs. *Statistics in Medicine* 2022; 41(30): 5767–5788.

391. Daimon T, Zohar S, O'Quigley J. Posterior maximization and averaging for Bayesian working model choice in the continual reassessment method. *Statistics in Medicine* 2011; 30(13): 1563–1573.

392. Wages NA, Ivanova A, Marchenko O. Practical designs for phase I combination studies in oncology. *Journal of biopharmaceutical statistics* 2016; 26(1): 150–166.

393. Mozgunov P, Jaki T, Gounaris I, Goddemeier T, Victor A, Grinberg M. Practical implementation of the partial ordering continual reassessment method in a Phase I combination-schedule dose-finding trial. *Statistics in Medicine* 2022; 41(30): 5789–5809.

394. Faries D. Practical modifications of the continual reassessment method for phase I cancer clinical trials. *Journal of biopharmaceutical statistics* 1994; 4(2): 147–164.

395. Polley MYC. Practical modifications to the time-to-event continual reassessment method for phase I cancer trials with fast patient accrual and late-onset toxicities. *Statistics in medicine* 2011; 30(17): 2130–2143.

396. Lee J, Thall PF, Msaouel P. Precision Bayesian phase I-II dose-finding based on utilities tailored to prognostic subgroups. *Statistics in medicine* 2021; 40(24): 5199–5217.

397. Lin X, Ji Y. Probability intervals of toxicity and efficacy design for dose-finding clinical trials in oncology. *Statistical Methods in Medical Research* 2021; 30(3): 843–856.

398. Xu Z, Lin X. Probability-of-decision interval 3+3 (POD-i3+3) design for phase I dose finding trials with late-onset toxicity. *Statistical Methods in Medical Research* 2022; 31(3): 534–548.

399. Meter EMV, Garrett-Mayer E, Bandyopadhyay D. Proportional odds model for dose-finding clinical trial designs with ordinal toxicity grading. *Statistics in medicine* 2011; 30(17): 2070–2080.

400. Paul RK, Rosenberger WF, Flournoy N. Quantile estimation following non-parametric phase I clinical trials with ordinal response. *Statistics in Medicine* 2004; 23(16): 2483–2495.

401. Mozgunov P, Jaki T, Paoletti X. Randomized dose-escalation designs for drug combination cancer trials with immunotherapy. *Journal of Biopharmaceutical Statistics* 2019; 29(2): 359–377.

402. Xue X, Foster MC, Ivanova A. Rapid enrollment design for finding the optimal dose in immunotherapy trials with ordered groups. *Journal of biopharmaceutical statistics* 2019; 29(4): 625–634.

403. Zohar S, Chevret S. Recent developments in adaptive designs for phase I/II dose-finding studies. *Journal of biopharmaceutical statistics* 2007; 17(6): 1071–1083.

404. Zhang H, Chiang AY, Wang J. Rejoinder: Improving the performance of Bayesian logistic regression model with overdose control in oncology dose-finding studies. *Statistics in Medicine* 2022; 41(27): 5497–5500.

405. Riviere MK, Dubois F, Zohar S. Response to comments on 'Competing designs for drug combination in phase I dose-finding clinical trials' by G. Yin, R. Lin and N. Wages. *Statistics in Medicine* 2015; 34(1): 23–26.

406. Hirakawa A, Matsui S. Response to letter to the editor by Dr Wages et al.. *Statistics in Medicine* 2014; 33(12): 2159–2160.

407. Bekele BN, Li Y, Ji Y. Risk-group-specific dose finding based on an average toxicity score. *Biometrics* 2010; 66(2): 541–548.

408. Yuan Y, Yin G. Robust EM continual reassessment method in oncology dose finding. *Journal of the American Statistical Association* 2011; 106(495): 818–831.

409. Guo W, Ji Y, Li D. R-TPI: rolling toxicity probability interval design to shorten the duration and maintain safety of phase I trials. *Journal of Biopharmaceutical Statistics* 2019; 29(3): 411–424.

410. Ji Y, Wang SJ. Safety Concerns of the 3+3 Design: A Comparison to the mTPI Design. *Topics in Applied Statistics: 2012 Symposium of the International Chinese Statistical Association* 2013: 125–135.

411. Zhang Y, Guo B, Cao S, Zhang C, Zang Y. SCI: A Bayesian adaptive phase I/II dose-finding design accounting for semi-competing risks outcomes for immunotherapy trials. *Pharmaceutical statistics* 2022; 21(5): 960–973.

412. Wages NA, Tait C. Seamless phase I/II adaptive design for oncology trials of molecularly targeted agents. *Journal of biopharmaceutical statistics* 2015; 25(5): 903–920.

413. Biard L, Lee SM, Cheng B. Seamless phase I/II design for novel anticancer agents with competing disease progression. *Statistics in medicine* 2021; 40(21): 4568–4581.

414. Jia X, Ivanova A, Lee SM. Selection of the initial design for the two-stage continual reassessment method. *Journal of biopharmaceutical statistics* 2017; 27(3): 495–506.

415. Clertant M, O'Quigley J. Semiparametric dose finding methods. *Journal of the Royal Statistical Society Series B: Statistical Methodology* 2017; 79(5): 1487–1508.

416. Clertant M, Wages NA, O'Quigley J. Semiparametric dose finding methods for partially ordered drug combinations. *Statistica Sinica* 2022; 32: 1983.

417. Clertant M, O'Quigley J. Semiparametric dose finding methods: special cases. *Journal of the Royal Statistical Society Series C: Applied Statistics* 2019; 68(2): 271–288.

418. Yuan Y, Yin G. Sequential continual reassessment method for two-dimensional dose finding. *Statistics in medicine* 2008; 27(27): 5664–5678.

419. Roth K. Sequential designs for dose escalation studies in oncology. *Communications in Statistics-Simulation and Computation* 2012; 41(7): 1131–1141.

420. Liu G, Rosenberger WF, Haines LM. Sequential designs for logistic phase I clinical trials. *Journal of Biopharmaceutical Statistics* 2006; 16(5): 605–621.

421. Cheung YK, Chappell R. Sequential designs for phase I clinical trials with late-onset toxicities. *Biometrics* 2000; 56(4): 1177–1182.

422. Cheung YK. Sequential implementation of stepwise procedures for identifying the maximum tolerated dose. *Journal of the American Statistical Association* 2007; 102(480): 1448–1461.

423. Iasonos A, O'Quigley J. Sequential monitoring of Phase I dose expansion cohorts. *Statistics in medicine* 2017; 36(2): 204–214.

424. Chen X, Zhang J, Jiang L, Yan F. Shotgun-2: A Bayesian phase I/II basket trial design to identify indication-specific optimal biological doses. *Statistical Methods in Medical Research* 2023; 32(3): 443–464.

425. Cheung YK. Simple benchmark for complex dose finding studies. *Biometrics* 2014; 70(2): 389–397.

426. Zhu X, Tian J. Simulation Analysis of Phase I Trial Designs in Oncology. *American Journal of Mathematical and Management Sciences* 2010; 30(1-2): 67–81.

427. Hather GJ, Mackey H. Some notable properties of the standard oncology phase I design. *Journal of Biopharmaceutical Statistics* 2009; 19(3): 543–555.

428. Goodman SN, Zahurak ML, Piantadosi S. Some practical improvements in the continual reassessment method for phase I studies. *Statistics in medicine* 1995; 14(11): 1149–1161.

429. Wages NA, Conaway MR. Specifications of a continual reassessment method design for phase I trials of combined drugs. *Pharmaceutical statistics* 2013; 12(4): 217–224.

430. Guo B, Li D, Yuan Y. SPIRIT: A seamless phase I/II randomized design for immunotherapy trials. *Pharmaceutical statistics* 2018; 17(5): 527–540.

431. Guan S. Statistical designs for early phases of cancer clinical trials. *Journal of biopharmaceutical statistics* 2012; 22(6): 1109–1126.

432. Liu J, He W, Quan H. Statistical properties of a modified accelerated design for phase I cancer clinical trials. *Communications in Statistics—Theory and Methods* 2007; 36(15): 2741–2759.

433. Lin Y, Shih WJ. Statistical properties of the traditional algorithm-based designs for phase I cancer clinical trials. *Biostatistics* 2001; 2(2): 203–215.

434. Lin R, Yin G. STEIN: A simple toxicity and efficacy interval design for seamless phase I/II clinical trials. *Statistics in medicine* 2017; 36(26): 4106–4120.

435. Cheung YK, Elkind MS. Stochastic approximation with virtual observations for dose-finding on discrete levels. *Biometrika* 2010; 97(1): 109–121.

436. Devlin SM, Iasonos A, O'Quigley J. Stopping rules for phase I clinical trials with dose expansion cohorts. *Statistical methods in medical research* 2022; 31(2): 334–347.

437. Curtis A, Smith B, Chapple AG. Subgroup-specific dose finding for phase I-II trials using Bayesian clustering. *Statistics in medicine* 2022; 41(16): 3164–3179.

438. Chapple AG, Thall PF. Subgroup-specific dose finding in phase I clinical trials based on time to toxicity allowing adaptive subgroup combination. *Pharmaceutical statistics* 2018; 17(6): 734–749.

439. Andrillon A, Chevret S, Lee SM, Biard L. Surv-CRM-12: A Bayesian phase I/II survival CRM for right-censored toxicity endpoints with competing disease progression. *Statistics in medicine* 2022; 41(29): 5753–5766.

440. Guo W, Zhong B. Target toxicity design for phase I dose-finding. *Statistical Theory and Related Fields* 2021; 5(2): 149–161.

441. Guo W, Ni Y, Ji Y. Teams: toxicity-and efficacy-based dose-insertion design with adaptive model selection for phase I/II dose-escalation trials in oncology. *Statistics in biosciences* 2015; 7: 432–459.

442. Li P, Liu R, Lin J, Ji Y. TEPI-2 and UBI: designs for optimal immuno-oncology and cell therapy dose finding with toxicity and efficacy. *Journal of biopharmaceutical statistics* 2020; 30(6): 979–992.

443. Stylianou M, Follmann DA. The accelerated biased coin up-and-down design in phase I trials. *Journal of biopharmaceutical statistics* 2004; 14(1): 249–260.

444. Jia N, Braun TM. The adaptive accelerated biased coin design for phase I clinical trials. *Journal of Applied Statistics* 2011; 38(12): 2911–2924.

445. Yuan S, Zhou T, Lin Y, Ji Y. The Ci3+3 design for dual-agent combination dose-finding clinical trials. *Journal of Biopharmaceutical Statistics* 2021; 31(6): 745–764.

446. Ishizuka N, Ohashi Y. The continual reassessment method and its applications: a Bayesian methodology for phase I cancer clinical trials. *Statistics in Medicine* 2001; 20(17-18): 2661–2681.

447. Yuan Z, Chappell R, Bailey H. The continual reassessment method for multiple toxicity grades: a Bayesian quasi-likelihood approach. *Biometrics* 2007; 63(1): 173–179.

448. Chevret S. The continual reassessment method in cancer phase I clinical trials: a simulation study. *Statistics in medicine* 1993; 12(12): 1093–1108.

449. Zohar S, Chevret S. The continual reassessment method: comparison of Bayesian stopping rules for dose-ranging studies. *Statistics in medicine* 2001; 20(19): 2827–2843.

450. Liu M, Wang SJ, Ji Y. The i3+3 design for phase I clinical trials. *Journal of biopharmaceutical statistics* 2020; 30(2): 294–304.

451. Lin X, Ji Y. The Joint i3+3 (Ji3+3) design for phase I/II adoptive cell therapy clinical trials. *Journal of Biopharmaceutical Statistics* 2020; 30(6): 993–1005.

452. Koopmeiners JS, Wey A. The randomized CRM: an approach to overcoming the long-memory property of the CRM. *Journal of biopharmaceutical statistics* 2017; 27(6): 1028–1042.

453. Ivanova A, Wang Y, Foster MC. The rapid enrollment design for phase I clinical trials. *Statistics in medicine* 2016; 35(15): 2516–2524.

454. Clertant M, O'Quigley J. The role of minimal sets in dose finding studies. *Journal of Nonparametric Statistics* 2018; 30(4): 1016–1031.

455. Azriel D, Mandel M, Rinott Y. The treatment versus experimentation dilemma in dose finding studies. *Journal of Statistical Planning and Inference* 2011; 141(8): 2759–2768.

456. O'Quigley J. Theoretical study of the continual reassessment method. *Journal of Statistical Planning and Inference* 2006; 136(6): 1765–1780.

457. Huang B, Chappell R. Three-dose–cohort designs in cancer phase I trials. *Statistics in Medicine* 2008; 27(12): 2070–2093.

458. Huang B, Kuan PF. Time-to-event continual reassessment method incorporating treatment cycle information with application to an oncology phase I trial. *Biometrical Journal* 2014; 56(6): 933–946.

459. Lin R, Yuan Y. Time-to-event model-assisted designs for dose-finding trials with delayed toxicity. *Biostatistics* 2020; 21(4): 807–824.

460. Zhou Y, Lin R, Lee JJ, et al. TITE-BOIN12: A Bayesian phase I/II trial design to find the optimal biological dose with late-onset toxicity and efficacy. *Statistics in medicine* 2022; 41(11): 1918–1931.

461. Takeda K, Morita S, Taguri M. TITE-BOIN-ET: time-to-event Bayesian optimal interval design to accelerate dose-finding based on both efficacy and toxicity outcomes. *Pharmaceutical statistics* 2020; 19(3): 335–349.

462. Takeda K, Xia Q, Liu S, Rong A. TITE-gBOIN: Time-to-event Bayesian optimal interval design to accelerate dose-finding accounting for toxicity grades. *Pharmaceutical Statistics* 2022; 21(2): 496–506.

463. Wheeler GM, Sweeting MJ, Mander AP. Toxicity-dependent feasibility bounds for the escalation with overdose control approach in phase I cancer trials. *Statistics in medicine* 2017; 36(16): 2499–2513.

464. Messer K, Natarajan L, Ball ED, Lane TA. Toxicity-evaluation designs for phase I/II cancer immunotherapy trials. *Statistics in medicine* 2010; 29(7-8): 712–720.

465. Li Y, Wang M, Cheung YK. Treatment and dose prioritization in early phase platform trials of targeted cancer therapies. *Journal of the Royal Statistical Society Series C: Applied Statistics* 2019; 68(2): 475–491.

466. Han Y, Liu H, Cao S, Zhang C, Zang Y. TSNP: A two-stage nonparametric phase I/II clinical trial design for immunotherapy. *Pharmaceutical statistics* 2021; 20(2): 282–296.

467. Wang K, Ivanova A. Two-dimensional dose finding in discrete dose space. *Biometrics* 2005; 61(1): 217–222.

468. O'Quigley J, Shen LZ, Gamst A. Two-sample continual reassessment method. *Journal of biopharmaceutical statistics* 1999; 9(1): 17–44.

469. Shimamura F, Hamada C, Matsui S, Hirakawa A. Two-stage approach based on zone and dose findings for two-agent combination phase I/II trials. *Journal of biopharmaceutical statistics* 2018; 28(6): 1025–1037.

470. Dragalin V, Fedorov VV, Wu Y. Two-stage design for dose-finding that accounts for both efficacy and safety. *Statistics in Medicine* 2008; 27(25): 5156–5176.

471. Tighiouart M. Two-stage design for phase I–II cancer clinical trials using continuous dose combinations of cytotoxic agents. *Journal of the Royal Statistical Society Series C: Applied Statistics* 2019; 68(1): 235–250.

472. Yin G, Zheng S, Xu J. Two-stage dose finding for cytostatic agents in phase I oncology trials. *Statistics in Medicine* 2013; 32(4): 644–660.

473. McGovern A, Chapple AG, Ma C. Two-stage subgroup-specific time-to-event (2S-Sub-TITE): An adaptive two-stage time-to-toxicity design for subgroup-specific dose finding in phase I oncology trials. *Pharmaceutical Statistics* 2022; 21(6): 1138–1148.

474. Petit C, Samson A, Morita S, et al. Unified approach for extrapolation and bridging of adult information in early-phase dose-finding paediatric studies. *Statistical methods in medical research* 2018; 27(6): 1860–1877.

475. Lin R, Yin G. Uniformly most powerful Bayesian interval design for phase I dose-finding trials. *Pharmaceutical statistics* 2018; 17(6): 710–724.

476. Chu PL, Lin Y, Shih WJ. Unifying CRM and EWOC designs for phase I cancer clinical trials. *Journal of statistical planning and inference* 2009; 139(3): 1146–1163.

477. Ahn C, Kang SH, Xie Y. *Optimal Biological Dose for Molecularly-Targeted Therapies*: 1-8; John Wiley & Sons, Ltd . 2007

478. Kpamegan E, Flournoy N. Up-and-down designs for selecting the dose with maximum success probability. *Sequential Analysis* 2008; 27(1): 78–96.

479. Jin IH, Liu S, Thall PF, Yuan Y. Using data augmentation to facilitate conduct of phase I–II clinical trials with delayed outcomes. *Journal of the American Statistical Association* 2014; 109(506): 525–536.

480. Thall PF, Nguyen HQ, Braun TM, Qazilbash MH. Using joint utilities of the times to response and toxicity to adaptively optimize schedule–dose regimes. *Biometrics* 2013; 69(3): 673–682.

481. Wages NA, Conaway MR, O'Quigley J. Using the time-to-event continual reassessment method in the presence of partial orders. *Statistics in medicine* 2013; 32(1): 131–141.

482. Gordon N, Willson J. Using toxicity grades in the design and analysis of cancer phase I clinical trials. *Statistics in Medicine* 1992; 11(16): 2063–2075.

483. Houede N, Thall PF, Nguyen H, Paoletti X, Kramar A. Utility-based optimization of combination therapy using ordinal toxicity and efficacy in phase I/II trials. *Biometrics* 2010; 66(2): 532–540.

484. Shi H, Cao J, Yuan Y, Lin R. uTPI: A utility-based toxicity probability interval design for phase I/II dose-finding trials. *Statistics in Medicine* 2021; 40(11): 2626–2649.

485. Ananthakrishnan R, Green S, Li D, LaValley M. 2D (2 dimensional) TEQR design for determining the optimal dose for safety and efficacy. *Contemporary clinical trials communications* 2019; 16: 100461.

486. Quintana M, Li DH, Albertson TM, Connor JT. A Bayesian adaptive phase 1 design to determine the optimal dose and schedule of an adoptive T-cell therapy in a mixed patient population. *Contemporary clinical trials* 2016; 48: 153–165.

487. Koopmeiners JS, Modiano J. A Bayesian adaptive phase I–II clinical trial for evaluating efficacy and toxicity with delayed outcomes. *Clinical Trials* 2014; 11(1): 38–48.

488. Schipper MJ, Yuan Y, Taylor JM, Ten Haken RK, Tsien C, Lawrence TS. A Bayesian dose-finding design for outcomes evaluated with uncertainty. *Clinical Trials* 2021; 18(3): 279–285.

489. Gönen M. A Bayesian evaluation of enrolling additional patients at the maximum tolerated dose in phase I trials. *Contemporary clinical trials* 2005; 26(2): 131–140.

490. Van Meter EM, Garrett-Mayer E, Bandyopadhyay D. Dose-finding clinical trial design for ordinal toxicity grades using the continuation ratio model: an extension of the continual reassessment method. *Clinical trials* 2012; 9(3): 303–313.

491. Li Z, Xu Z, Zhang A, Qi G, Li Z. A clinical phase I dose-finding design with adaptive shrinking boundaries for drug combination trials. *BMC Medical Research Methodology* 2023; 23(1): 57.

492. Abbas R, Rossoni C, Jaki T, Paoletti X, Mozgunov P. A comparison of phase I dose-finding designs in clinical trials with monotonicity assumption violation. *Clinical Trials* 2020; 17(5): 522–534.

493. Iasonos A, Wilton AS, Riedel ER, Seshan VE, Spriggs DR. A comprehensive comparison of the continual reassessment method to the standard 3+3 dose escalation scheme in Phase I dose-finding studies. *Clinical Trials* 2008; 5(5): 465–477.

494. Xu J, Zhang D, Mu R. A dose-finding design for phase I clinical trials based on Bayesian stochastic approximation. *BMC Medical Research Methodology* 2022; 22(1): 258.

495. Chiuzan C, Garrett-Mayer E, Yeatts SD. A likelihood-based approach for computing the operating characteristics of the 3+3 phase I clinical trial design with extensions to other A+ B designs. *Clinical Trials* 2015; 12(1): 24–33.

496. Ji Y, Liu P, Li Y, Nebiyou Bekele B. A modified toxicity probability interval method for dose-finding trials. *Clinical trials* 2010; 7(6): 653–663.

497. Boonstra PS, Braun TM, Chase EC. A modular framework for early-phase seamless oncology trials. *Clinical Trials* 2021; 18(3): 303–313.

498. Doussau A, Thiébaut R, Paoletti X. Dose-finding design using mixed-effect proportional odds model for longitudinal graded toxicity data in phase I oncology clinical trials. *Statistics in medicine* 2013; 32(30): 5430–5447.

499. Tang N, Wang S, Ye G. A nonparametric Bayesian continual reassessment method in single-agent dose-finding studies. *BMC medical research methodology* 2018; 18: 1–13.

500. Zhou H, Chen C, Sun L, Zeng Z. A novel framework of Bayesian optimal interval design for phase I trials with late-onset toxicities. *Contemporary Clinical Trials* 2021; 105: 106404.

501. Chen Z, Krailo MD, Azen SP, Tighiouart M. A novel toxicity scoring system treating toxicity response as a quasi-continuous variable in Phase I clinical trials. *Contemporary clinical trials* 2010; 31(5): 473–482.

502. Wages NA, Slingluff Jr CL, Petroni GR. A phase I/II adaptive design to determine the optimal treatment regimen from a set of combination immunotherapies in high-risk melanoma. *Contemporary Clinical Trials* 2015; 41: 172–179.

503. Pan H, Xie F, Liu P, Xia J, Ji Y. A phase I/II seamless dose escalation/expansion with adaptive randomization scheme (SEARS). *Clinical Trials* 2014; 11(1): 49–59.

504. Chapple AG, Wojcik JJ, McDaniel LS. A regression based phase I clinical trial for late-onset toxicities without clinician elicitation. *Contemporary clinical trials communications* 2019; 14: 100327.

505. Barrett JS, Jayaraman B, Patel D, Skolnik JM. A SAS-based solution to evaluate study design efficiency of phase I pediatric oncology trials via discrete event simulation. *Computer methods and programs in biomedicine* 2008; 90(3): 240–250.

506. Onar-Thomas A, Xiong Z. A simulation-based comparison of the traditional method, Rolling-6 design and a frequentist version of the continual reassessment method with special attention to trial duration in pediatric Phase I oncology trials. *Contemporary clinical trials* 2010; 31(3): 259–270.

507. Li C, Sun H, Cheng C, Tang L, Pan H. A software tool for both the maximum tolerated dose and the optimal biological dose finding trials in early phase designs. *Contemporary clinical trials communications* 2022; 30: 100990.

508. Sun Z, Braun TM. A two-dimensional biased coin design for dual-agent dose-finding trials. *Clinical Trials* 2015; 12(6): 596–607.

509. Su Z. A two-stage algorithm for designing phase I cancer clinical trials for two new molecular entities. *Contemporary clinical trials* 2010; 31(1): 105–107.

510. Mu R, Xu G, Liu G, Pan H. A two-stage Bayesian adaptive design for minimum effective dose (MinED)-based dosing-finding trials. *Contemporary Clinical Trials* 2021; 108: 106504.

511. Wages NA, Varhegyi N. A web application for evaluating phase I methods using a non-parametric optimal benchmark. *Clinical Trials* 2017; 14(5): 553–557.

512. Sposto R, Groshen S. A wide-spectrum paired comparison of the properties of the Rolling 6 and 3+3 Phase I study designs. *Contemporary clinical trials* 2011; 32(5): 694–703.

513. Wages NA, Reed DR, Keng MK, Conaway MR, Petroni GR. Adapting isotonic dose-finding to a dynamic set of drug combinations with application to a phase I leukemia trial. *Clinical Trials* 2021; 18(3): 314–323.

514. Kojima M. Adaptive design for identifying maximum tolerated dose early to accelerate dose-finding trial. *BMC Medical Research Methodology* 2022; 22(1): 97.

515. Zang Y, Lee JJ, Yuan Y. Adaptive designs for identifying optimal biological dose for molecularly targeted agents. *Clinical Trials* 2014; 11(3): 319–327.

516. Hu B, Bekele BN, Ji Y. Adaptive dose insertion in early phase clinical trials. *Clinical Trials* 2013; 10(2): 216–224.

517. Wages NA, Fadul CE. Adaptive dose-finding based on safety and feasibility in early-phase clinical trials of adoptive cell immunotherapy. *Clinical Trials* 2020; 17(2): 157–165.

518. Mu R, Hu Z, Xu G, Pan H. An adaptive gBOIN design with shrinkage boundaries for phase I dose-finding trials. *BMC Medical Research Methodology* 2021; 21: 1–12.

519. Yang S, Wang SJ, Ji Y. An integrated dose-finding tool for phase I trials in oncology. *Contemporary clinical trials* 2015; 45: 426–434.

520. Jiang H, Liu Y, Su Z. An optimization algorithm for designing phase I cancer clinical trials. *Contemporary clinical trials* 2008; 29(2): 102–108.

521. Ananthakrishnan R, Lin R, He C, Chen Y, Li D, LaValley M. An overview of the BOIN design and its current extensions for novel early-phase oncology trials. *Contemporary Clinical Trials Communications* 2022; 28: 100943.

522. Yada S, Hamada C. Analyses of drug combinations using missing data shortens trial periods in phase I/II oncology trials. *Contemporary Clinical Trials Communications* 2017; 7: 73–80.

523. Barnett H, Boix O, Kontos D, Jaki T. Backfilling cohorts in phase I dose-escalation studies. *Clinical Trials* 2023; 20(3): 261–268.

524. Broglio KR, Sandalic L, Albertson T, Berry SM. Bayesian dose escalation in oncology with sharing of information between patient populations. *Contemporary clinical trials* 2015; 44: 56–63.

525. Zhao D, Zhu J, Wang L. Bayesian interval-based oncology dose-finding design with repeated quasi-continuous toxicity model. *Contemporary Clinical Trials* 2021; 102: 106265.

526. Takahashi A, Suzuki T. Bayesian optimization for estimating the maximum tolerated dose in phase I clinical trials. *Contemporary Clinical Trials Communications* 2021; 21: 100753.

527. Ivanova A, Oron AP. Up-and-Down and Escalation Designs. *Methods and Applications of Statistics in Clinical Trials: Concepts, Principles, Trials, and Design* 2014: 353–361.

528. Braun TM, Alonzo TA. Beyond the 3+3 method: expanded algorithms for dose-escalation in phase I oncology trials of two agents. *Clinical trials* 2011; 8(3): 247–259.

529. Cheung YK. Commentary on'Small-sample behavior of novel phase I cancer trial designs'. *Clinical Trials* 2013; 10(1): 86.

530. Ji L, Alonzo TA. Comparison of design methods for a safety run-in phase of a phase II clinical trial. *Clinical Trials* 2023; 20(2): 181–191.

531. Chapple AG, Thall PF. Comparison of Phase I-II designs with parametric or semi-parametric models using two different risk-benefit trade-off criteria. *Contemporary clinical trials* 2020; 97: 106099.

532. Braun TM, Mercier F. Extending the Continual Reassessment Method to accommodate step-up dosing in Phase I trials. *Statistics in Medicine* 2022; 41(20): 3975–3990.

533. Zhang H, Chiang AY, Wang J. Improving the performance of Bayesian logistic regression model with overdose control in oncology dose-finding studies. *Statistics in Medicine* 2022; 41(27): 5463–5483.

534. Bayar MA, Ivanova A, Le Teuff G. CRM2DIM: A SAS macro for implementing the dual-agent Bayesian continual reassessment method. *Computer methods and programs in biomedicine* 2019; 176: 211–223.

535. Liu R, Lin J, Li P. Design considerations for phase I/II dose finding clinical trials in Immuno-oncology and cell therapy. *Contemporary clinical trials* 2020; 96: 106083.

536. Pallmann P, Wan F, Mander AP, et al. Designing and evaluating dose-escalation studies made easy: the MoDEsT web app. *Clinical Trials* 2020; 17(2): 147–156.

537. Braun TM, Levine JE, Ferrara JL. Determining a maximum tolerated cumulative dose: dose reassignment within the TITE-CRM. *Controlled clinical trials* 2003; 24(6): 669–681.

538. Riviere MK, Jourdan JH, Zohar S. dfcomb: An R-package for phase I/II trials of drug combinations. *Computer Methods and Programs in Biomedicine* 2016; 125: 117–133.

539. Toumazi A, Comets E, Alberti C, et al. dfpk: an R-package for Bayesian dose-finding designs using pharmacokinetics (PK) for phase I clinical trials. *Computer Methods and Programs in Biomedicine* 2018; 157: 163–177.

540. Carlin BP, Zhong W, Koopmeiners JS. Discussion of 'Small-sample behavior of novel phase I cancer trial designs' by Assaf P Oron and Peter D Hoff. *Clinical Trials* 2013; 10(1): 81-85. doi: 10.1177/1740774512469313

541. Chen Z, Yuan Y, Li Z, et al. Dose escalation with over-dose and under-dose controls in phase I/II clinical trials. *Contemporary clinical trials* 2015; 43: 133–141.

542. Chen Z, Tighiouart M, Kowalski J. Dose escalation with overdose control using a quasi-continuous toxicity score in cancer Phase I clinical trials. *Contemporary Clinical Trials* 2012; 33(5): 949–958.

543. Bové DS, Yeung WY, Palermo G, Jaki T. Model-based dose escalation designs in R with crmpPack. *Journal of Statistical Software* 2019; 89: 1–22.

544. Wages NA, Conaway MR, O'Quigley J. Dose-finding design for multi-drug combinations. *Clinical Trials* 2011; 8(4): 380–389.

545. Doussau A, Asselain B, Le Deley M, et al. Dose-finding designs in pediatric phase I clinical trials: comparison by simulations in a realistic timeline framework. *Contemporary clinical trials* 2012; 33(4): 657–665.

546. Ji Y, Li Y, Nebiyou Bekele B. Dose-finding in phase I clinical trials based on toxicity probability intervals. *Clinical Trials* 2007; 4(3): 235–244.

547. Hoering A, Mitchell A, LeBlanc M, Crowley J. Early phase trial design for assessing several dose levels for toxicity and efficacy for targeted agents. *Clinical Trials* 2013; 10(3): 422–429.

548. Thall PF, Herrick RC, Nguyen HQ, Venier JJ, Norris JC. Effective sample size for computing prior hyperparameters in Bayesian phase I–II dose-finding. *Clinical Trials* 2014; 11(6): 657–666.

549. Cunanan KM, Koopmeiners JS. Efficacy/toxicity dose-finding using hierarchical modeling for multiple populations. *Contemporary Clinical Trials* 2018; 71: 162–172.

550. Wages NA, Millard TA, Dillon PM, Brenin CM, Petroni GR. Efficient dose-finding for drug combination studies involving a shift in study populations. *Contemporary Clinical Trials Communications* 2020; 17: 100519.

551. Chen Z, Cui Y, Owonikoko TK, et al. Escalation with overdose control using all toxicities and time to event toxicity data in cancer phase I clinical trials. *Contemporary clinical trials* 2014; 37(2): 322–332.

552. Zhu Y, Hwang WT, Li Y. Evaluating the effects of design parameters on the performances of phase I trial designs. *Contemporary clinical trials communications* 2019; 15: 100379.

553. Cunanan K, Koopmeiners JS. Evaluating the performance of copula models in phase I-II clinical trials under model misspecification. *BMC Medical Research Methodology* 2014; 14: 1–11.

554. Wages NA, Bagley E. Evaluation of irrational dose assignment definitions using the continual reassessment method. *Clinical Trials* 2019; 16(6): 665–672.

555. Micallef S, Sostelly A, Zhu J, Baverel PG, Mercier F. Exposure driven dose escalation design with overdose control: Concept and first real life experience in an oncology phase I trial. *Contemporary Clinical Trials Communications* 2022; 26: 100901.

556. Ananthakrishnan R, Green S, Li D, LaValley M. Extensions of the mTPI and TEQR designs to include non-monotone efficacy in addition to toxicity for optimal dose determination for early phase immunotherapy oncology trials. *Contemporary clinical trials communications* 2018; 10: 62–76.

557. Li S, Xie XJ, Heitjan DF. Flexible, rule-based dose escalation: The cohort-sequence design. *Contemporary clinical trials communications* 2020; 17: 100541.

558. Yin G, Yang Z. Fractional design: an alternative paradigm for late-onset toxicities in oncology dose-finding studies. *Contemporary clinical trials communications* 2020; 19: 100650.

559. Duan Y, Wang SJ, Ji Y. Hi3+3: A model-assisted dose-finding design borrowing historical data. *Contemporary Clinical Trials* 2021; 109: 106437.

560. Wheeler GM, Mander AP, Bedding A, et al. How to design a dose-finding study using the continual reassessment method. *BMC medical research methodology* 2019; 19: 1–15.

561. Brock K, Billingham L, Copland M, Siddique S, Sirovica M, Yap C. Implementing the EffTox dose-finding design in the Matchpoint trial. *BMC medical research methodology* 2017; 17: 1–15.

562. Iasonos A, Zohar S, O'Quigley J. Incorporating lower grade toxicity information into dose finding designs. *Clinical Trials* 2011; 8(4): 370–379.

563. Iasonos A, O'Quigley J. Integrating the escalation and dose expansion studies into a unified Phase I clinical trial. *Contemporary clinical trials* 2016; 50: 124–134.

564. Chen Z, Zheng Y, Wang Z, Kutner M, Curran WJ, Kowalski J. Interactive calculator for operating characteristics of phase I cancer clinical trials using standard 3+3 designs. *Contemporary Clinical Trials Communications* 2018; 12: 145–153.

565. Park Y. Interval design to identify the optimal biological dose for immunotherapy. *Contemporary Clinical Trials Communications* 2022; 30: 101005.

566. Yuan Z, Chappellb R. Isotonic designs for phase I cancer clinical trials with multiple risk groups. *Clinical Trials* 2004; 1(6): 499–508.

567. Leung DHY, Wang YG. Isotonic designs for phase I trials. *Controlled clinical trials* 2001; 22(2): 126–138.

568. Conaway M. Isotonic designs for phase I trials in partially ordered groups. *Clinical Trials* 2017; 14(5): 491–498.

569. Pan H, Lin R, Zhou Y, Yuan Y. Keyboard design for phase I drug-combination trials. *Contemporary Clinical Trials* 2020; 92: 105972.

570. Legedza AT, Ibrahim JG. Longitudinal design for phase I clinical trials using the continual reassessment method. *Controlled clinical trials* 2000; 21(6): 574–588.

571. Lee SM, Cheung YK. Model calibration in the continual reassessment method. *Clinical Trials* 2009; 6(3): 227–238.

572. Muenz DG, Braun TM, Taylor JM. Modeling adverse event counts in phase I clinical trials of a cytotoxic agent. *Clinical Trials* 2018; 15(4): 386–397.

573. Qiu Y, Zhao Y, Liu H, Cao S, Zhang C, Zang Y. Modified isotonic regression based phase I/II clinical trial design identifying optimal biological dose. *Contemporary clinical trials* 2023; 127: 107139.

574. Kramar A, Houédé N, Paoletti X. np1: a computer program for dose escalation strategies in phase I clinical trials. *Computer methods and programs in biomedicine* 2007; 88(1): 8–17.

575. Lee SY, Munafo A, Girard P, Goteti K. Optimization of dose selection using multiple surrogates of toxicity as a continuous variable in phase I cancer trial. *Contemporary clinical trials* 2022; 113: 106657.

576. Wages NA, Conaway MR, O'Quigley J. Performance of two-stage continual reassessment method relative to an optimal benchmark. *Clinical Trials* 2013; 10(6): 862–875.

577. Braun TM, Thall PF. Optimizing Schedule of Administration in Phase I Clinical Trials. *Wiley Encyclopedia of Clinical Trials* 2007: 1–7.

578. Wages NA, Varhegyi N. pocrm: an R-package for phase I trials of combinations of agents. *Computer Methods and Programs in Biomedicine* 2013; 112(1): 211–218.

579. Ewings S, Saunders G, Jaki T, Mozgunov P. Practical recommendations for implementing a Bayesian adaptive phase I design during a pandemic. *BMC Medical Research Methodology* 2022; 22(1): 25.

580. Werkhoven vE, Hinsley S, Frangou E, et al. Practicalities in running early-phase trials using the time-to-event continual reassessment method (TiTE-CRM) for interventions with long toxicity periods using two radiotherapy oncology trials as examples. *BMC Medical Research Methodology* 2020; 20: 1–10.

581. Hirakawa A, Tanaka Y, Kaneko S. Pragmatic dose-escalation methods incorporating relative dose intensity assessment for molecularly targeted agents in phase I trials. *Contemporary Clinical Trials Communications* 2019; 16: 100489.

582. Drubay D, Collette L, Paoletti X. Proportional odds assumption for modeling longitudinal ordinal multiple toxicity outcomes in dose finding studies of targeted agents: A pooled analysis of 54 studies. *Contemporary Clinical Trials Communications* 2020; 17: 100529.

583. O'Connell NS, Wages NA, Garrett-Mayer E. Quasi-partial order continual reassessment method: Applying toxicity scores to cancer dose-finding drug combination trials. *Contemporary Clinical Trials* 2023; 125: 107050.

584. Chen Z, Krailo MD, Sun J, Azen SP. Range and trend of expected toxicity level (ETL) in standard A+ B designs: a report from the Children's Oncology Group. *Contemporary Clinical Trials* 2009; 30(2): 123–128.

585. Yin J, Paoletti X, Sargent DJ, Mandrekar SJ. Repeated measures dose-finding design with time-trend detection in the presence of correlated toxicity data. *Clinical Trials* 2017; 14(6): 611–620.

586. Wages NA, Conaway MR. Revisiting isotonic phase I design in the era of model-assisted dose-finding. *Clinical Trials* 2018; 15(5): 524–529.

587. Zhu J, Bové DS, Liao Z, Beyer U, Yung G, Sarkar S. Rolling continual reassessment method with overdose control: An efficient and safe dose escalation design. *Contemporary Clinical Trials* 2021; 107: 106436.

588. Kuen Cheung Y. Sample size formulae for the Bayesian continual reassessment method. *Clinical Trials* 2013; 10(6): 852–861.

589. Zohar S, O'Quigley J. Sensitivity of dose-finding studies to observation errors. *Contemporary clinical trials* 2009; 30(6): 523–530.

590. Mao X, Cheung YK. Sequential designs for individualized dosing in phase I cancer clinical trials. *Contemporary clinical trials* 2017; 63: 51–58.

591. Rossoni C, Bardet A, Geoerger B, Paoletti X. Sequential or combined designs for phase I/II clinical trials? A simulation study. *Clinical Trials* 2019; 16(6): 635–644.

592. Horton BJ, Wages NA, Conaway MR. Shift models for dose-finding in partially ordered groups. *Clinical Trials* 2019; 16(1): 32–40.

593. Jiang L, Li R, Yan F, Yap TA, Yuan Y. Shotgun: A Bayesian seamless phase I-II design to accelerate the development of targeted therapies and immunotherapy. *Contemporary Clinical Trials* 2021; 104: 106338.

594. Liu CA, Braun TM. Parametric non-mixture cure models for schedule finding of therapeutic agents. *Journal of the Royal Statistical Society Series C: Applied Statistics* 2009; 58(2): 225–236.

595. Oron AP, Hoff PD. Small-sample behavior of novel phase I cancer trial designs. *Clinical Trials* 2013; 10(1): 63–80.

596. Oron AP, Hoff PD. Small-sample behavior of novel phase I designs: Rejoinder. *Clinical Trials* 2013; 10(1): 88–92.

597. Zohar S, Latouche A, Taconnet M, Chevret S. Software to compute and conduct sequential Bayesian phase I or II dose-ranging clinical trials with stopping rules. *Computer methods and programs in biomedicine* 2003; 72(2): 117–125.

598. Mi G, Bian Y, Wang X, Zhang W. SPA: Single patient acceleration in oncology dose-escalation trials. *Contemporary Clinical Trials* 2021; 105: 106378.

599. Ananthakrishnan R, Green S, Chang M, Doros G, Massaro J, LaValley M. Systematic comparison of the statistical operating characteristics of various Phase I oncology designs. *Contemporary clinical trials communications* 2017; 5: 34–48.

600. Braun TM. The bivariate continual reassessment method: extending the CRM to phase I trials of two competing outcomes. *Controlled clinical trials* 2002; 23(3): 240–256.

601. Garrett-Mayer E. The continual reassessment method for dose-finding studies: a tutorial. *Clinical trials* 2006; 3(1): 57–71.

602. Siegel J. The importance of loss functions: A note on the evolution of the Toxicity Probability Interval design. *Contemporary Clinical Trials Communications* 2021; 22: 100694.

603. Zhao L, Lee J, Mody R, Braun TM. The superiority of the time-to-event continual reassessment method to the rolling six design in pediatric oncology phase I trials. *Clinical Trials* 2011; 8(4): 361–369.

604. Blanchard MS, Longmate JA. Toxicity equivalence range design (TEQR): a practical phase I design. *Contemporary clinical trials* 2011; 32(1): 114–121.

605. Salter A, O'Quigley J, Cutter GR, Aban IB. Two-group time-to-event continual reassessment method using likelihood estimation. *Contemporary clinical trials* 2015; 45: 340–345.

606. Liu S, Cai C, Ning J. Up-and-down designs for phase I clinical trials. *Contemporary clinical trials* 2013; 36(1): 218–227.

607. Takahashi A, Suzuki T. A Curve Free Method based on Bayesian Optimization for Oncology Phase I Clinical Trials. *Proceedings of the 2nd International Conference on Statistics: Theory and Applications (ICSTA'20)* 2020(106).

608. Andrillon A, Biard L, Lee SM. Incorporating patient-reported outcomes in dose-finding clinical trials with continuous patient enrollment. *Journal of biopharmaceutical statistics* 2023: 1–12.

609. Bagley EM, Wages NA. Dose-finding based on feasibility and late-onset toxicity in adoptive cell therapy trials. *Journal of Biopharmaceutical Statistics* 2024; 34(2): 151–163.

610. Clertant M, Iasonos A, O'Quigley J. Evaluation of early phase dose finding algorithms in heterogeneous populations. *Journal of Statistical Planning and Inference* 2024; 231: 106080.

611. Duan Y, Yuan S, Ji Y, Mueller P. A Unified Decision Framework for Phase I Dose-Finding Designs. *Statistics in Biosciences* 2024; 16(1): 69–85.

612. Guo B, Yuan Y. DROID: dose-ranging approach to optimizing dose in oncology drug development. *Biometrics* 2023; 79(4): 2907–2919.

613. Hashizume K, Tsuchida J, Sozu T. Copula-based model for incorporating single-agent historical data into dual-agent phase I cancer trials. *Statistics in Biopharmaceutical Research* 2024; 16(1): 71–88.

614. Jiménez JL, Zheng H. A Bayesian adaptive design for dual-agent phase I–II oncology trials integrating efficacy data across stages. *Biometrical Journal* 2023; 65(7): 2200288.

615. Jin H, Yin G. Time-to-event calibration-free odds design: a robust efficient design for phase I trials with late-onset outcomes. *Pharmaceutical Statistics* 2023; 22(5): 773–783.

616. Kojima M. Data-dependent early completion of dose-finding trials for drug-combination. *Statistical Methods in Medical Research* 2023; 32(4): 820–828.

617. Lin LH, Han Y, Zhang R, Guo B. Biomarker-based precision dose finding for immunotherapy combined with radiotherapy. *Biometrical Journal* 2023; 65(7): 2200246.

618. Ling H, Shi H, Yuan N, Ji Y, Lin X. qTPI: A quasi-toxicity probability interval design for phase I trials with multiple-grade toxicities. *Statistical Methods in Medical Research* 2023; 32(7): 1389–1402.

619. Saha PT, Fine JP, Ivanova A. CRM and partial order CRM with adaptive rescaling for dose-finding in immunotherapy trials with a continuous outcome. *Statistics in Medicine* 2023; 42(14): 2409–2419.

620. Takeda K, Yamaguchi Y, Taguri M, Morita S. TITE-gBOIN-ET: Time-to-event generalized Bayesian optimal interval design to accelerate dose-finding accounting for ordinal graded efficacy and toxicity outcomes. *Biometrical Journal* 2023; 65(7): 2200265.

621. Takeda K, Zhu J, Li R, Yamaguchi Y. A Bayesian optimal interval design for dose optimization with a randomization scheme based on pharmacokinetics outcomes in oncology. *Pharmaceutical Statistics* 2023; 22(6): 1104–1115.

622. Thall PF, Zang Y, Yuan Y. Generalized phase I-II designs to increase long term therapeutic success rate. *Pharmaceutical statistics* 2023; 22(4): 692–706.

623. Wang Z, Zhang J, Xia T, He R, Yan F. A Bayesian phase I–II clinical trial design to find the biological optimal dose on drug combination. *Journal of Biopharmaceutical Statistics* 2024; 34(4): 582–595.

624. Yamaguchi Y, Takeda K, Yoshida S, Maruo K. Optimal biological dose selection in dose-finding trials with model-assisted designs based on efficacy and toxicity: a simulation study. *Journal of Biopharmaceutical Statistics* 2024; 34(3): 379–393.

625. Zhang J, Chen X, Li B, Yan F. A comparative study of adaptive trial designs for dose optimization. *Pharmaceutical Statistics* 2023; 22(5): 797–814.

626. Günhan BK, Weber S, Seroutou A, Friede T. Phase I dose-escalation oncology trials with sequential multiple schedules. *BMC Medical Research Methodology* 2021; 21: 1–14.

627. Yeung WY, Reigner B, Beyer U, et al. Bayesian adaptive dose-escalation designs for simultaneously estimating the optimal and maximum safe dose based on safety and efficacy. *Pharmaceutical statistics* 2017; 16(6): 396–413.

628. Hoering A, LeBlanc M, Crowley J. Seamless phase I-II trial design for assessing toxicity and efficacy for targeted agents. *Clinical Cancer Research* 2011; 17(4): 640–646.

629. Li DH, Whitmore JB, Guo W, Ji Y. Toxicity and efficacy probability interval design for phase I adoptive cell therapy dose-finding clinical trials. *Clinical Cancer Research* 2017; 23(1): 13–20.

630. Zhou T, Guo W, Ji Y. Pod-tpi: Probability-of-decision toxicity probability interval design to accelerate phase I trials. *Statistics in Biosciences* 2020; 12(2): 124–145.

631. Doussau A, Thiebaut R, Geoerger B, et al. A new approach to integrate toxicity grade and repeated treatment cycles in the analysis and reporting of phase I dose-finding trials. *Annals of Oncology* 2015; 26(2): 422–428.

632. Braun TM, Thall PF, Nguyen H, De Lima M. Simultaneously optimizing dose and schedule of a new cytotoxic agent. *Clinical Trials* 2007; 4(2): 113–124.

633. Simon R, Rubinstein L, Arbuck SG, Christian MC, Freidlin B, Collins J. Accelerated titration designs for phase I clinical trials in oncology. *Journal of the National Cancer Institute* 1997; 89(15): 1138–1147.

634. O'Quigley J, Iasonos A, Bornkamp B. *Handbook of methods for designing, monitoring, and analyzing dose-finding trials*. CRC press . 2017.

635. Liu M, Ji Y, Lin J. The Total i3+3 (Ti3+3) Design for Assessing Multiple Types and Grades of Toxicity in Phase I Trials. *The New England Journal of Statistics in Data Science* 2022; 2(1): 72–85.

636. Lin R, Zhou Y, Yan F, Li D, Yuan Y. BOIN12: Bayesian optimal interval phase I/II trial design for utility-based dose finding in immunotherapy and targeted therapies. *JCO precision oncology* 2020; 4: 1393–1402.

637. Yuan Y, Lin R, Li D, Nie L, Warren KE. Time-to-event Bayesian optimal interval design to accelerate phase I trials. *Clinical Cancer Research* 2018; 24(20): 4921–4930.

638. Zhang W, Wang X, Yang P. A new design of the continual reassessment method. *Communications in Statistics-Simulation and Computation* 2021; 50(7): 2015–2024.

639. Diniz MA, Kim S, Tighiouart M. A Bayesian adaptive design in cancer phase I trials using dose combinations with ordinal toxicity grades. *Stats* 2020; 3(3): 221–238.

640. O'Quigley J, Zohar S. Retrospective robustness of the continual reassessment method. *Journal of biopharmaceutical statistics* 2010; 20(5): 1013–1025.

641. O'Quigley J. Retrospective analysis of sequential dose-finding designs. *Biometrics* 2005; 61(3): 749–756.

642. Qiu Y, Li M. A randomized Bayesian phase I-II dose optimization design for combination cancer therapies with progression-free survival end point. *BMC Medical Research Methodology* 2025; 25(1): 214.

643. Ji Y, Zhang Y, Ji A, Kummar S. The generalized 3+3 (G3) design for Phase I trials: A design for clinicians. *Contemporary Clinical Trials* 2025: 108077.

644. Daniells L, Jaki T, Dayimu A, et al. Seamless monotherapy-combination phase I dose-escalation model-based design. *Clinical Trials* 2025; 22(4): 430–441.

645. Zhao Y, Liu R, Lin J, Yuan Y. BARD: A seamless two-stage dose optimization design integrating backfill and adaptive randomization. *Clinical Trials* 2025; 22(4): 393–404.

646. Wong WK, Ryeznik Y, Sverdlov O, et al. Nature-inspired metaheuristics for optimizing dose-finding and computationally challenging clinical trial designs. *Clinical Trials* 2025; 22(4): 422–429.

647. Bagley EM, Wages NA. Dose-finding designs for cell therapy cancer clinical trials evaluating drug-combinations. *Contemporary Clinical Trials* 2025; 153: 107894.

648. Liu J, Yuan S, Deng Q, Ji Y. The modified combo i3+3 design for novel-novel combination dose-finding trials in oncology. *Contemporary Clinical Trials* 2025; 152: 107857.

649. Chen X, Zhang J, Li B, Yan F. Determining doses for backfill cohorts based on patient-reported outcome. *BMC Medical Research Methodology* 2024; 24(1): 270.

650. Patel A, Brock K, Slade D, et al. Implementing the time-to-event continual reassessment method in the presence of partial orders in a phase I head and neck cancer trial. *BMC Medical Research Methodology* 2024; 24(1): 11.

651. Liao JJ, Asatiani E, Liu Q, Hou K. Three steps toward dose optimization for oncology dose finding. *Contemporary Clinical Trials Communications* 2024; 40: 101329.

652. Pin L, Villar SS, Dehbi HM. Implementing and assessing Bayesian response-adaptive randomisation for backfilling in dose-finding trials. *Contemporary Clinical Trials* 2024; 142: 107567.

653. Biard L, Andrillon A, Silva R, Lee S. Dose optimization for cancer treatments with considerations for late-onset toxicities. *Clinical trials* 2024; 21(3): 322–330.

654. Chiuzan C, Dehbi HM. The 3+3 design in dose-finding studies with small sample sizes: Pitfalls and possible remedies. *Clinical Trials* 2024; 21(3): 350–357.

655. Thall PF, Garrett-Mayer E, Wages NA, Halabi S, Cheung YK. Current issues in dose-finding designs: A response to the US Food and Drug Adminstration's Oncology Center of Excellence Project Optimus. *Clinical Trials* 2024; 21(3): 267–272.

656. Yuan Y, Zhou H, Liu S. Statistical and practical considerations in planning and conduct of dose-optimization trials. *Clinical Trials* 2024; 21(3): 273–286.

657. Zang Y, Guo B, Qiu Y, Liu H, Opyrchal M, Lu X. Adaptive phase I–II clinical trial designs identifying optimal biological doses for targeted agents and immunotherapies. *Clinical Trials* 2024; 21(3): 298–307.

658. Msaouel P, Lee J, Thall PF. Risk–benefit trade-offs and precision utilities in phase I-II clinical trials. *Clinical Trials* 2024; 21(3): 287–297.

659. Celum C, Horton BJ, Conaway M. The quasi-CRM shift method for partially ordered groups. *Contemporary Clinical Trials* 2024; 136: 107400.

660. Widmer LA, Weber S, Xu Y, Weber HJ. Towards Efficient Time-to-Event Dose-Escalation Guidance of Multi-Cycle Cancer Therapies. *Statistics in medicine* 2025; 44(20-22): e70229.

661. Zhao S, Thall PF, Yuan Y, Lee J, Msaouel P, Zang Y. Precision generalized phase I-II designs. *Biometrics* 2025; 81(3): ujaf043.

662. Qiu Y, Lu M, Han Y, et al. A model-free phase I/II dose optimization design for immunotherapy trials. *Statistical Methods in Medical Research* 2025: 09622802251340246.

663. Porter S, Eaton A, Murray TA. Dose selection criteria to identify the optimal dose based on ranked efficacy-toxicity outcomes without reliance on clinical utilities. *Statistical methods in medical research* 2025: 09622802251327691.

664. Falke CE, Koopmeiners JS. Accounting for Carryover Toxicity in Phase I Clinical Trials with Intra-Patient Dose Escalation. *Statistics in Medicine* 2025; 44(10-12): e70059.

665. Chen K, Takeda K, Yuan Y. COMIC: A Bayesian Dose Optimization Design for Drug Combination in Multiple Indications With Application to CAR-T Therapies. *Statistics in Medicine* 2025; 44(10-12): e70107.

666. Li M, Guo Z, Qiu Y. UNITED: A Unified Transparent and Efficient Phase I/II Trial Design for Dose Optimization Accounting for Ordinal Graded, Continuous and Mixed Toxicity and Efficacy Endpoints. *Statistics in Medicine* 2025; 44(10-12): e70098.

667. Hatayama T, Yasui S. CUSUMIN Combination: A Cumulative Sum Interval Design for Phase I Cancer Drug-Combination Trials. *Pharmaceutical Statistics* 2025; 24(3): e70007.

668. Yan Y, Lin R, Guan T, Shi H, Lin X. A Generalized Phase I/II Dose Optimization Trial Design With Multi-Categorical and Multi-Graded Outcomes. *Statistics in Medicine* 2025; 44(7): e70049.

669. Guo B, Liu S. A Phase I Dose-Finding Design Incorporating Intra-Patient Dose Escalation. *Pharmaceutical statistics* 2025; 24(2): e2461.

670. Zhang Z. A Likelihood Perspective on Dose-Finding Study Designs in Oncology. *Pharmaceutical Statistics* 2025; 24(2): e2445.

671. Zhang J, Takeda K, Takeuchi M, Komatsu K, Zhu J, Yamaguchi Y. A Model-Based Trial Design With a Randomization Scheme Considering Pharmacokinetics Exposure for Dose Optimization in Oncology. *Pharmaceutical Statistics* 2025; 24(2): e2454.

672. Qiu Y, Li M. A Bayesian Dynamic Model-Based Adaptive Design for Oncology Dose Optimization in Phase I/II Clinical Trials. *Pharmaceutical Statistics* 2025; 24(2): e2451.

673. Sun H, Tu J. PKBOIN-12: A Bayesian Optimal Interval Phase I/II Design Incorporating Pharmacokinetics Outcomes to Find the Optimal Biological Dose. *Pharmaceutical Statistics* 2025; 24(2): e2444.

674. Liu M, Lin J, Mi G, Lorenzato C, Chen X, Ji Y. PoD-BIN: A probability of decision Bayesian interval design for time-to-event dose-finding trials with multiple toxicity grades. *The Annals of Applied Statistics* 2025; 19(1): 147–168.

675. Takeda K, Zhu J, Hirakawa A. BF-BOIN-ET: a backfill Bayesian optimal interval design using efficacy and toxicity outcomes for dose optimization. *Pharmaceutical Statistics* 2025; 24(2): e2470.

676. Ollier A, Mozgunov P. On Inclusion of Covariates in Model Based Dose Finding Clinical Trial Designs. *Statistics in Medicine* 2025; 44(3-4): e10337.

677. Wang J, Tiwari R. Optimal dose selection in phase I/II dose finding trial with contextual bandits: a case study and practical recommendations. *Journal of Biopharmaceutical Statistics* 2025: 1–27.

678. Lu M, Yuan Y, Liu S. A Bayesian pharmacokinetics integrated phase I–II design to optimize dose-schedule regimes. *Biostatistics* 2025; 26(1): kxae034.

679. Barnett H, Boix O, Kontos D, Jaki T. Joint TITE-CRM: A Design for Dose Finding Studies for Therapies with Late-Onset Safety and Activity Outcomes. *Statistics in Biopharmaceutical Research* 2025; 17(1): 149–160.

680. Guo X, Kent S, Maity A, Zhong W. Optimization of EWOC principle in BLRM design for phase 1 oncology trials. *Journal of Biopharmaceutical Statistics* 2025; 35(3): 407–423.

681. Wages NA, Lin R. Isotonic phase I cancer clinical trial design utilizing patient-reported outcomes. *Statistics in Biopharmaceutical Research* 2025; 17(1): 36–45.

682. Sadachi R, Sato H, Fujiwara T, Hirakawa A. Enhancement of Bayesian optimal interval design by accounting for overdose and underdose errors trade-offs. *Journal of Biopharmaceutical Statistics* 2025; 35(1): 1–20.

683. Zhang Z, Li Y. Generalized Likelihood Ratios for Designing Dose Optimization Studies of Targeted Therapies. *Statistics in Biopharmaceutical Research* 2025; 17(1): 8–16.

684. Qiu Y, Li M. A robust Bayesian dose optimization design with backfill and randomization for phase I/II clinical trials. *Statistical Methods in Medical Research* 2025; 34(11): 2219–2234.

685. Chen X, Mozgunov P, Baird RD, Jaki T. Using circulating tumor DNA as a novel biomarker of efficacy for dose-finding designs in oncology. *Statistical Methods in Medical Research* 2025: 09622802251350457.

686. Wang J, Li Z, Yung G, Fridlyand J, Li C, Zhu J. Evaluations of backfill strategies in dose optimization through simulation studies for phase I trials in oncology. *Statistics in Biopharmaceutical Research* 2025: 1–11.

687. Siegel JM. Quality principles in Phase I dose escalation design. *Journal of Biopharmaceutical Statistics* 2025: 1–9.

688. Fu C, Zhou S, Lee JJ. Posterior Predictive Design for Phase I Clinical Trials. *Journal of the American Statistical Association* 2025: 1–12.

689. Park J, Park M, Lee JW. A comparative study of dose-finding models with late-onset toxicities. *Communications in Statistics-Simulation and Computation* 2025: 1–12.

690. Kojima M. Early completion based on adjacent dose information for model-assisted designs to accelerate maximum tolerated dose finding. *The International Journal of Biostatistics* 2025.

691. Lu M, Zhang J, Yuan Y, Lin R. Comb-BOIN12: A Utility-Based Bayesian Optimal Interval Design for Dose Optimization in Cancer Drug-Combination Trials. *Statistics in Biopharmaceutical Research* 2025; 17(2): 266–276.

692. D'Angelo G, Gong M, Marshall J, Yuan Y, Li X. U-MET: Utility-Based Dose Optimization Approach for Multiple-Dose Randomized Trial Designs. *Statistics in Biopharmaceutical Research* 2025; 17(2): 211–221.

693. Silva RB, Cheng B, Carvajal RD, Lee SM. Dose Individualization for Phase I Cancer Trials With Broadened Eligibility. *Statistics in Medicine* 2024; 43(29): 5534–5547.

694. Porter S, Murray TA, Eaton A. Phase I/II Design for Selecting Subgroup-Specific Optimal Biological Doses for Prespecified Subgroups. *Statistics in Medicine* 2024; 43(28): 5401–5411.

695. Zhang J, Wages NA, Lin R. SFU: Surface-Free Utility-Based Design for Dose Optimization in Cancer Drug Combination Trials. *Statistics in Biosciences* 2024; 16(3): 854–881.

696. Park Y, Chang W. A Personalized Dose-Finding Algorithm Based on Adaptive Gaussian Process Regression. *Pharmaceutical Statistics* 2024; 23(6): 1181–1205.

697. Guo J, Lu M, Wan I, Wang Y, Han L, Zang Y. T3+3: 3+3 Design With Delayed Outcomes. *Pharmaceutical Statistics* 2024; 23(6): 959–970.

698. Celum C, Conaway M. A model-assisted design for partially or completely ordered groups. *Pharmaceutical Statistics* 2024; 23(6): 906–927.

699. Wang Y. Dual-agent dose-finding in Phase I clinical trial—An extension of rapid enrollment design. *Statistics in Medicine* 2024; 43(22): 4361–4371.

700. Fang J, Yin G. Fractional accumulative calibration-free odds (f-aCFO) design for delayed toxicity in phase I clinical trials. *Statistics in Medicine* 2024; 43(17): 3210–3226.

701. Yang CH, Cheng G, Lin R. On the relative conservativeness of Bayesian logistic regression method in oncology dose-finding studies. *Pharmaceutical statistics* 2024; 23(4): 585–594.

702. Xia Q, Takeda K, Yamaguchi Y, Zhang J. A generalized Bayesian optimal interval design for dose optimization in immunotherapy. *Pharmaceutical Statistics* 2024; 23(4): 480–494.

703. Kakizume T, Takeda K, Taguri M, Morita S. BOIN-ETC: A Bayesian optimal interval design considering efficacy and toxicity to identify the optimal dose combinations. *Statistical Methods in Medical Research* 2024; 33(4): 716–727.

704. Yang C, Li Y. An extended Bayesian semi-mechanistic dose-finding design for phase I oncology trials using pharmacokinetic and pharmacodynamic information. *Statistics in medicine* 2024; 43(4): 689–705.

705. Barnett H, George M, Skanji D, Saint-Hilary G, Jaki T, Mozgunov P. A comparison of model-free phase I dose escalation designs for dual-agent combination therapies. *Statistical Methods in Medical Research* 2024; 33(2): 203–226.

706. Zhao Y, Liu R, Lin J, Chi A, Davies S. DOD-BART: machine learning-based dose optimization design incorporating patient-level prognostic factors via Bayesian additive regression trees. *Journal of Biopharmaceutical Statistics* 2024: 1–16.

707. Sun H, Lin HY, Tu J, Ananthakrishnan R, Kim E. Statistical operating characteristics of current early phase dose finding designs with toxicity and efficacy in oncology. *Journal of Biopharmaceutical Statistics* 2024: 1–21.

708. Deng Q, Zhu L, Weiss B, Aanur P, Gao L. Strategies for successful dose optimization in oncology drug development: a practical guide. *Journal of Biopharmaceutical Statistics* 2024: 1–15.

709. Chen K, Wang L, Yuan Y. MT-Keyboard: A Bayesian Model-Assisted Interval Design to Account for Toxicity Grades and Types for Phase I Trials. *Statistics in biopharmaceutical research* 2024; 16(3): 305–314.

710. Wei X, Li X, Guo Z. Leveraging pharmacokinetic parameters as covariate in Bayesian logistic regression model to optimize dose selection in early phase oncology trial. *Journal of Biopharmaceutical Statistics* 2024: 1–22.

711. Alam MI, Bogacka B, Coad DS. A constrained optimum adaptive design for dose finding in early phase clinical trials. *Journal of Biopharmaceutical Statistics* 2024: 1–26.

712. Yuan S, Huang Z, Liu J, Ji Y. Pharmacometrics-Enabled DOse OPtimization (PEDOOP) for seamless phase I-II trials in oncology. *Journal of Biopharmaceutical Statistics* 2024: 1–20.

713. Zhou T, Ji Y. Statistical Frameworks for Oncology Dose-Finding Designs with Late-Onset Toxicities: A Review. *Statistical Science* 2024; 39(2): 243–261.

714. Liu R, Yuan Y, Sen S, et al. Design Strategy and Consideration for Oncology Dose-Optimization: An Industry Perspective. *Statistics in Biopharmaceutical Research* 2024; 16(3): 338–347.

715. Chen K, Zhao Y, Liu M, Lin J, Liu R. DOD-Combo: Bayesian dose finding design in combination trials with meta-analytic-predictive prior. *Journal of Biopharmaceutical Statistics* 2024: 1–18.

716. Dejardin D, Huang B, Yuan Y, Beyer U, Fridlyand J, Zhu J. Dose optimization for novel oncology agents: Design options and strategies. *Statistics in Biopharmaceutical Research* 2024; 16(3): 326–337.

717. Zhang L, Bayman EO, Zamba K. A modified Huber loss function for continual reassessment methods in clinical trials. *Sequential Analysis* 2024; 43(1): 28–48.

718. Tu J, Chen Z. Bayesian dose escalation with overdose and underdose control utilizing all toxicities in Phase I/II clinical trials. *Biometrical Journal* 2024; 66(1): 2200189.

719. Zhang J, Yan F, Wages NA, Lin R. Local continual reassessment methods for dose finding and optimization in drug-combination trials. *Statistical Methods in Medical Research* 2023; 32(10): 2049–2063.

720. Chien I, Hernandez JG, Turner RE. Safe exploration in dose finding clinical trials with heterogeneous participants. In: Springer. ; 2023: 51–59.

721. Yuan Y, Hess KR, Hilsenbeck SG, Gilbert MR. Bayesian optimal interval design: a simple and well-performing design for phase I oncology trials. *Clinical Cancer Research* 2016; 22(17): 4291–4301.

722. Liao JJ, Zhou F, Zhou H, Petruzzelli L, Hou K, Asatiani E. A hybrid design for dose-finding oncology clinical trials. *International Journal of Cancer* 2022; 151(9): 1602–1610.
